# Morphology controls nonlinear donor acceptor thermodynamics in divinylbenzene-based copolymers

**DOI:** 10.1016/j.isci.2026.117141

**Published:** 2026-08-13

**Authors:** Tayssir Hamieh

**Affiliations:** 1 Faculty of Science and Engineering, Maastricht University, P.O. Box 616, Maastricht 6200 MD, the Netherlands; 2 Institut de Science des Matériaux de Mulhouse, Université de Haute-Alsace, CNRS, IS2M UMR 7361, 68100 Mulhouse, France; 3 Laboratory of Materials, Catalysis, Environment and Analytical Methods (MCEMA), Faculty of Sciences, Lebanese University, Beirut P.O. Box 6573/14, Beirut, Lebanon

**Keywords:** Adsorption thermodynamics, Polymer interfaces, Surface morphology, Lewis acid-base interactions, Inverse gas chromatography, Specific surface area, Molecular footprint, Interfacial thermodynamics, Surface heterogeneity

## Abstract

Understanding how surface morphology governs molecular adsorption remains a fundamental challenge in interfacial science. Here, inverse gas chromatography at infinite dilution is combined with a generalized five-parameter Lewis acid-base model to investigate adsorption on divinylbenzene-based copolymers. Statistical model discrimination demonstrates that nonlinear donor-acceptor coupling and curvature are intrinsic features of polymer adsorption energetics. Independent thermo-geometric analysis further reveals that the adsorbed molecular footprint follows a common quadratic dependence on temperature and specific surface area, establishing a direct coupling between adsorption geometry and energetics. Quantitative correlations between geometric and energetic descriptors demonstrate that both originate from the same morphology-controlled interfacial field. These findings establish a unified thermodynamic framework in which specific surface area governs molecular packing and interaction strength, providing a predictive basis for understanding and engineering adsorption phenomena at heterogeneous polymer interfaces.

## Highlights

• Surface morphology controls adsorption energetics in DVB copolymers
• Nonlinear Lewis interactions outperform classical linear models
• Molecular footprints follow coupled thermo-geometric scaling laws
• Energetics and adsorption geometry define one thermodynamic framework

## Graphical abstract

**Figure:**
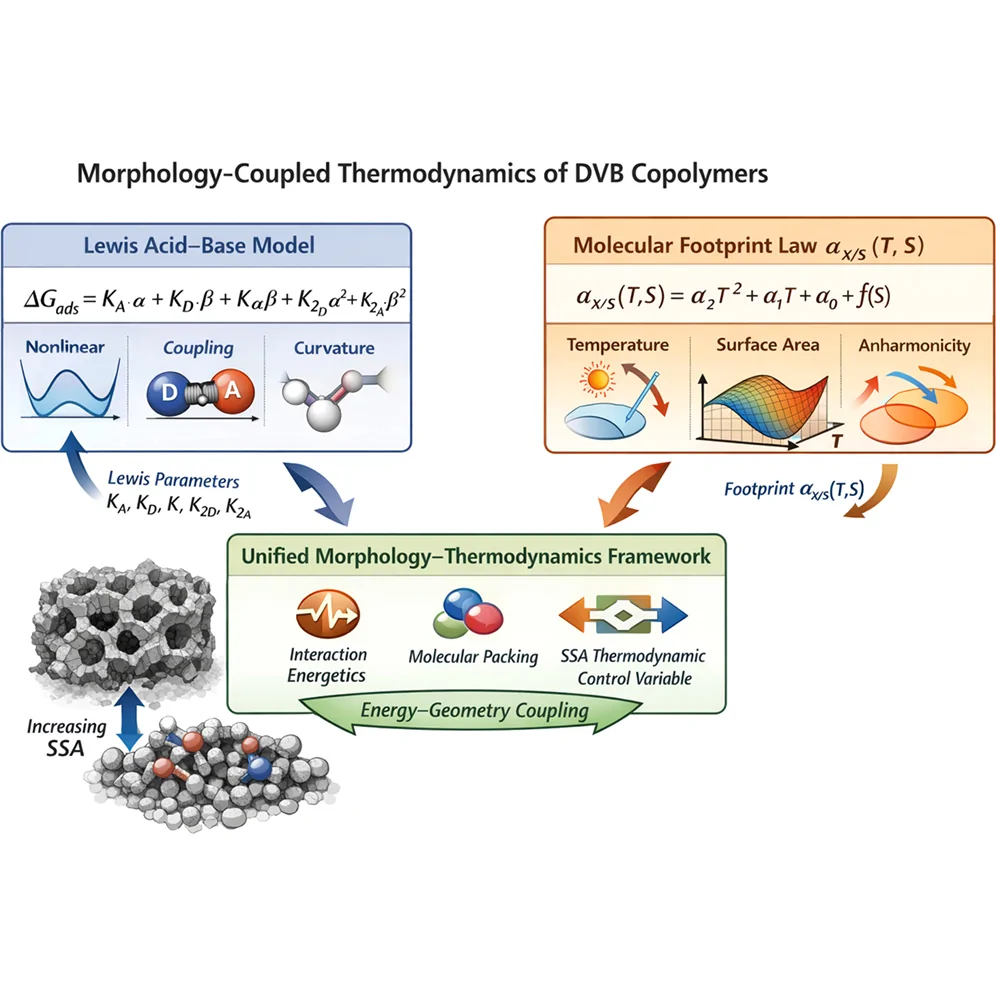

## Teaser

Thermodynamics; Materials science; Polymers

## Introduction

The thermodynamic description of adsorption on polymeric and highly cross-linked copolymer surfaces remains a central challenge in surface science. Unlike crystalline solids or idealized model surfaces, polymeric networks exhibit intrinsic structural disorder, energetic heterogeneity, and morphology-dependent accessibility that strongly influence intermolecular interactions.^1^^,^^2^^,^^3^^,^^4^^,^^5^^,^^6^^,^^7^^,^^8^^,^^9^^,^^10^^,^^11^^,^^12^^,^^13^^,^^14^^,^^15^^,^^16^^,^^17^ In rigid DVB-based copolymers, adsorption occurs within a complex interfacial landscape shaped by cross-link density, pore topology, and specific surface area (SSA). As a consequence, adsorption cannot be interpreted solely as a static energetic process but must be understood as a coupled energetic-geometric phenomenon.^17^^,^^18^^,^^19^^,^^20^^,^^21^

Inverse gas chromatography at infinite dilution (IGC-ID) has emerged as one of the most powerful experimental techniques for probing polymer surface thermodynamics at the molecular scale. By analyzing retention behavior of well-defined probe molecules, IGC provides access to fundamental quantities such as Gibbs-free energy of adsorption, London dispersive surface energy, and specific (acid-base) interaction parameters.^21^^,^^22^^,^^23^^,^^24^^,^^25^^,^^26^^,^^27^^,^^28^^,^^29^^,^^30^^,^^31^^,^^32^^,^^33^^,^^34^ Over several decades, this technique has enabled detailed characterization of polymeric materials ranging from elastomers and block copolymers to highly cross-linked resins. Nevertheless, most conventional IGC interpretations remain grounded in purely energetic formalisms, often assuming temperature-independent adsorption geometries and linear additivity of interaction contributions.^35^^,^^36^^,^^37^ For highly cross-linked polymer networks synthesized from divinylbenzene (DVB) and chlorodivinylbenzene (CDVB) monomers, such assumptions are incomplete. These materials combine high cross-link density with tunable porosity and significant surface heterogeneity. Previous investigations have shown that adsorption distance and dispersive interactions exhibit explicit temperature dependence and scale with SSA. This thermomechanical perspective established that adsorption geometry cannot be treated as a fixed parameter but instead responds to thermal excitation and morphological constraints. The bilinear dependencies observed between adsorption distance, temperature, and SSA demonstrated that morphology and thermodynamics are intrinsically coupled.

However, while adsorption distance provides insight into intermolecular separation, the lateral packing state of adsorbed molecules has not been systematically examined. The effective two-dimensional molecular footprint *a*_*X*/*S*_—representing the projected interfacial area occupied by an adsorbed molecule—contains direct information about configurational freedom, lateral repulsion, and entropic contributions. In classical IGC treatments, this footprint is often treated as a secondary geometric parameter derived from energetic quantities, without explicit consideration of its temperature and morphology dependence.^29^^,^^30^^,^^31^^,^^32^^,^^33^^,^^34^ Whether *a*_*X*/*S*_ follows a generalized quadratic thermo-geometric relationship and how it scales with SSA in cross-linked copolymers remain open questions.

A second unresolved issue concerns the adequacy of traditional two-parameter Lewis acid-base models. Linear donor-acceptor descriptions implicitly assume independent and homogeneous interaction sites. Yet DVB-based networks exhibit structural heterogeneity and confinement effects that may induce nonlinear coupling between acidic and basic interactions. Statistical discrimination between nested models has rarely been applied systematically to polymeric systems, leaving unanswered whether higher-order interaction terms are physically required or merely mathematical refinements.^38^^,^^39^^,^^40^

Previous studies^37^^,^^40^^,^^41^^,^^42^^,^^43^ introduced nonlinear Lewis acid-base models for adsorption energetics and independently reformulated the molecular surface area of adsorbed species as a temperature-dependent thermodynamic quantity. Despite these advances, the connection between adsorption energetics and adsorption geometry remained unresolved. The present work demonstrates that both observables obey coherent morphology-dependent scaling relationships governed by SSA, thereby establishing a unified thermo-geometric framework that quantitatively links interfacial energetics, molecular packing, and surface morphology.

In the present work, these two fundamental gaps were addressed through a unified thermo-geometric analysis of solvent adsorption on a family of DVB- and CDVB-based copolymers. Using temperature-resolved IGC measurements for nineteen carefully selected probe molecules spanning dispersive, polar aprotic, hydrogen-bonding, and aromatic classes, the evolution of the molecular interfacial area *a*_*X*/*S*_(*T*) was investigated across the copolymer series. It was demonstrated that the footprint obeys a generalized quadratic dependence on temperature and SSA, revealing intrinsic anharmonicity and explicit morphology-temperature coupling. The coefficients of this quadratic expansion themselves scale systematically with S, establishing $a_{X/S}\left(T,S\right)$ as a morphology-controlled thermodynamic state function.

Concurrently, the description of specific adsorption energetics was revisited using nested Lewis acid-base formulations ranging from classical two-parameter models to a five-parameter nonlinear representation. Statistical model selection based on R^2^, *RMSE*, Akaike Information Criterion (*AIC*), and Bayesian Information Criterion (*BIC*) reveals that nonlinear coupling and curvature terms are decisively required for all copolymers. Importantly, the same morphological hierarchy governing the molecular footprint also governs the Lewis interaction amplitudes, demonstrating that adsorption energetics and adsorption geometry respond to a common underlying structural field.

These findings elevate SSA from a simple structural descriptor to an interfacial thermodynamic control variable. In this framework, morphology simultaneously modulates intermolecular separation, donor-acceptor alignment, nonlinear interaction strength, and two-dimensional thermal expansion of the adsorbed layer. Adsorption on DVB-based copolymers therefore emerges as a coupled energy-geometry-entropy process rather than a linear superposition of surface energy components.

By integrating temperature-dependent molecular footprint analysis with statistically validated nonlinear Lewis energetics, this study establishes a unified morphology-thermodynamics framework for cross-linked copolymer interfaces. The resulting description provides deeper physical insight into how topology, temperature, and intermolecular interactions collectively determine adsorption behavior, and it offers a more rigorous foundation for predicting and tailoring surface properties of polymeric materials.

## Results

To elucidate the role of morphology in governing adsorption behavior, the temperature-resolved geometric characteristics of the adsorbed layer were examined and subsequently correlated with intermolecular energetics. The experimental findings reveal a coherent copolymer-dependent hierarchy that simultaneously influences molecular packing, thermal expansion, and donor-acceptor interaction strength. The temperature dependence of the molecular interfacial footprint was first analyzed.

### Adsorbed molecular footprints expand systematically with temperature across all DVB-based copolymers

The analysis begins with the temperature dependence of the molecular interfacial area *a*_*X*/*S*_(*T*), a geometric thermodynamic observable that directly reflects the lateral packing and configurational freedom of adsorbed molecules on DVB-based copolymer surfaces.

The molecular interfacial area *a*_*X*/*S*_(*T*) provides direct thermodynamic insight into the two-dimensional packing state of solvent molecules adsorbed at the copolymer surface. Unlike conventional surface energy parameters, which quantify interaction strength, the footprint *a*_*X*/*S*_ reflects the geometric and entropic projection of adsorption: it represents the effective lateral area occupied by a molecule in its adsorbed state under ID conditions. Consequently, the temperature evolution of *a*_*X*/*S*_(*T*) probes the thermal expansion behavior and configurational freedom of the interfacial layer.

**Figure 1:**
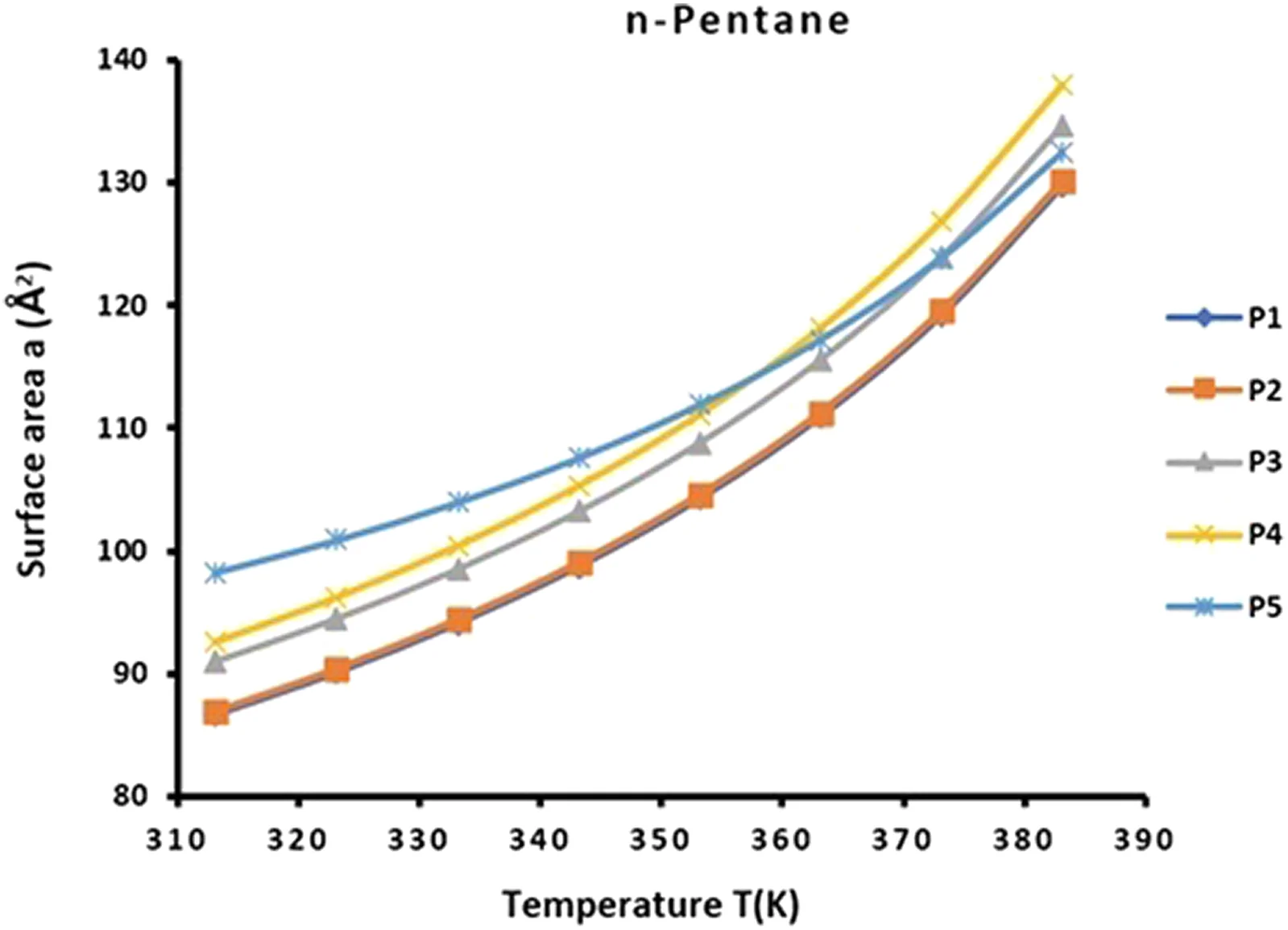
Temperature dependence of the molecular interfacial area, *a*_*X*/*S*_(*T*), for *n*-pentane adsorbed on the five DVB-based copolymers P1–P5

**Figure 2:**
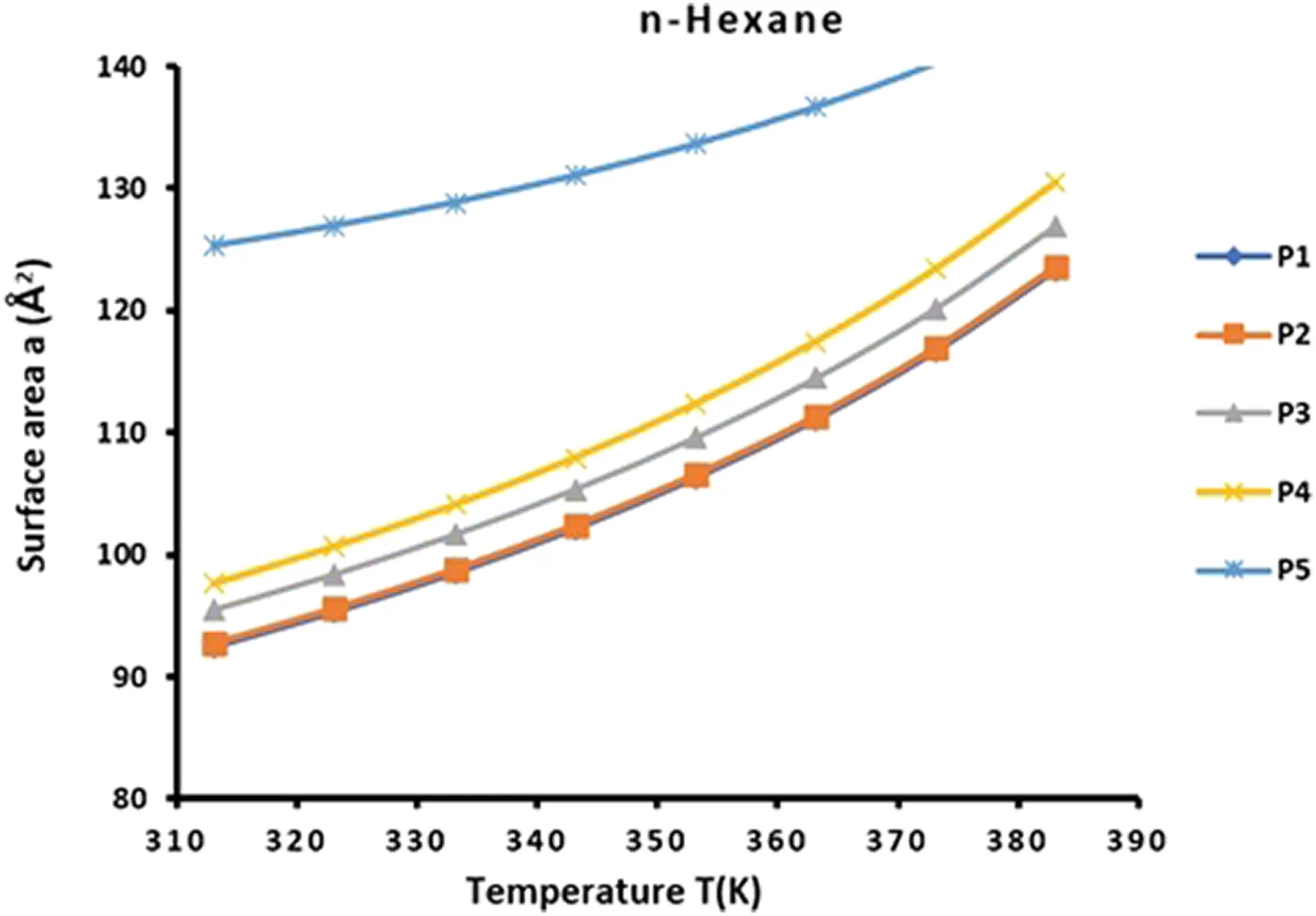
Temperature dependence of the molecular interfacial area, *a*_*X*/*S*_(*T*), for *n*-hexane adsorbed on the five DVB-based copolymers P1–P5

**Figure 3:**
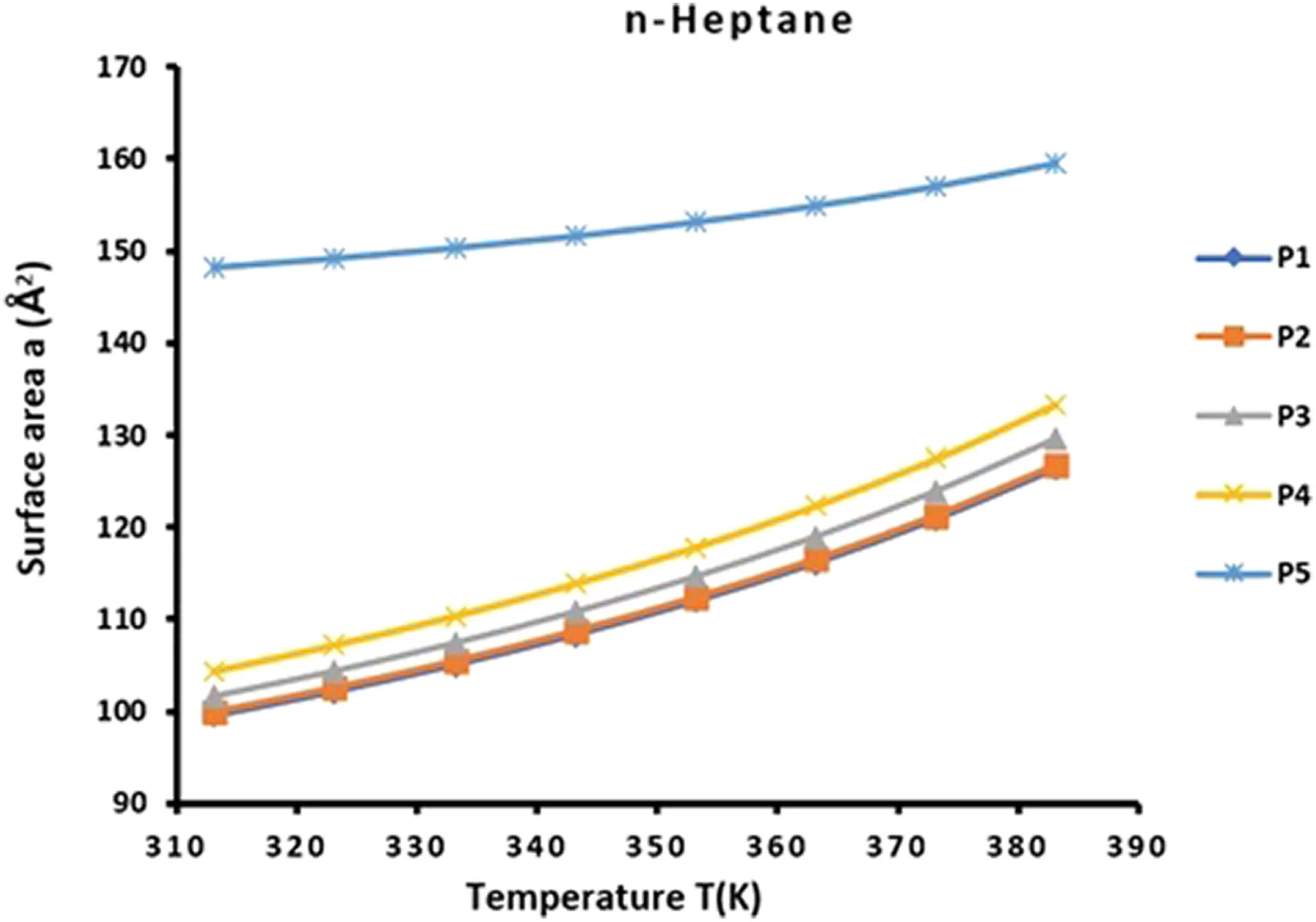
Temperature dependence of the molecular interfacial area, *a*_*X*/*S*_(*T*), for *n*-heptane adsorbed on the five DVB-based copolymers P1–P5

**Figure 4:**
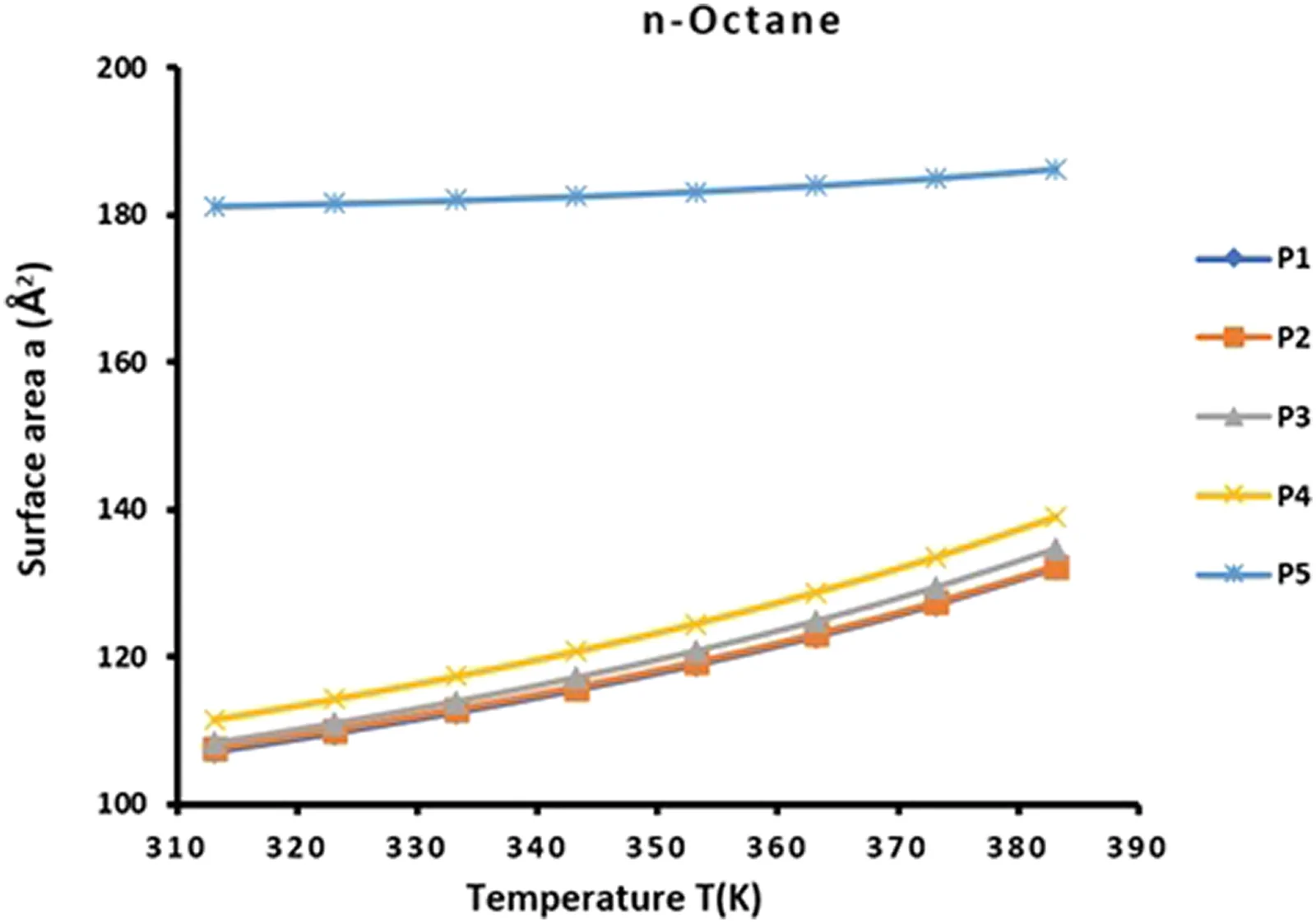
Temperature dependence of the molecular interfacial area, *a*_*X*/*S*_(*T*), for *n*-octane adsorbed on the five DVB-based copolymers P1–P5

**Figure 5:**
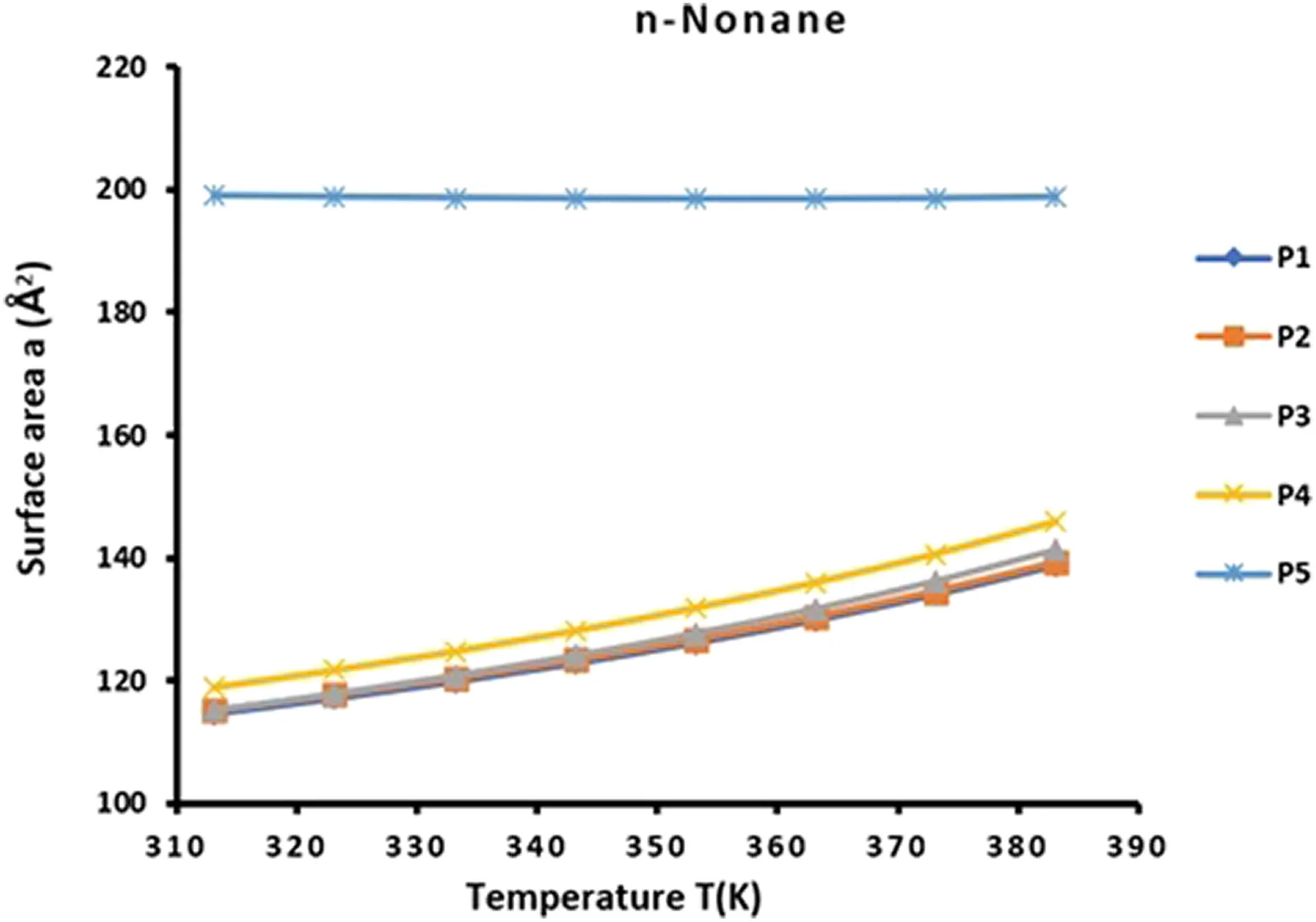
Temperature dependence of the molecular interfacial area, *a*_*X*/*S*_(*T*), for *n*-nonane adsorbed on the five DVB-based copolymers P1–P5

**Figure 6:**
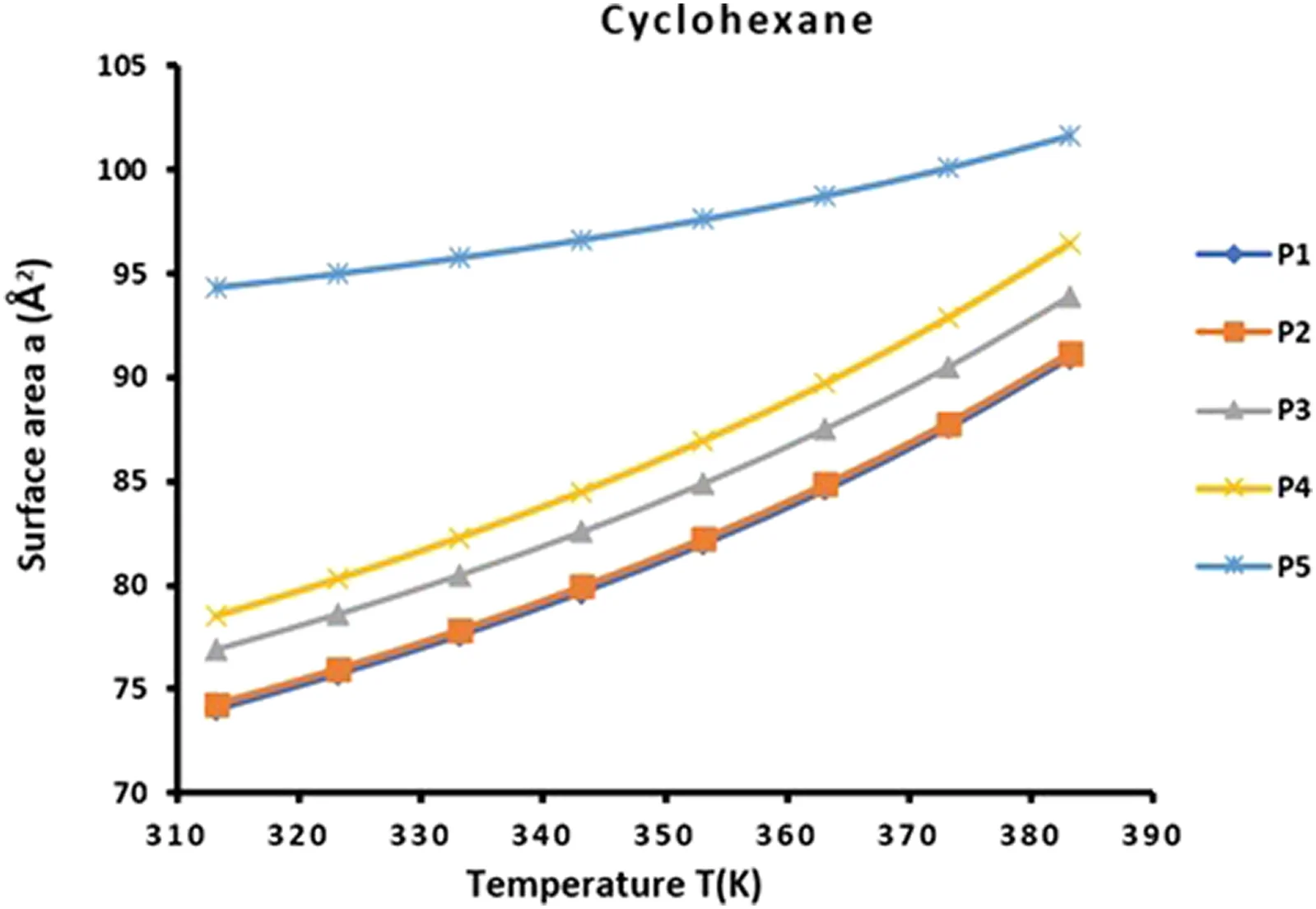
Temperature dependence of the molecular interfacial area, *a*_*X*/*S*_(*T*), for cyclohexane adsorbed on the five DVB-based copolymers P1–P5

**Figure 7:**
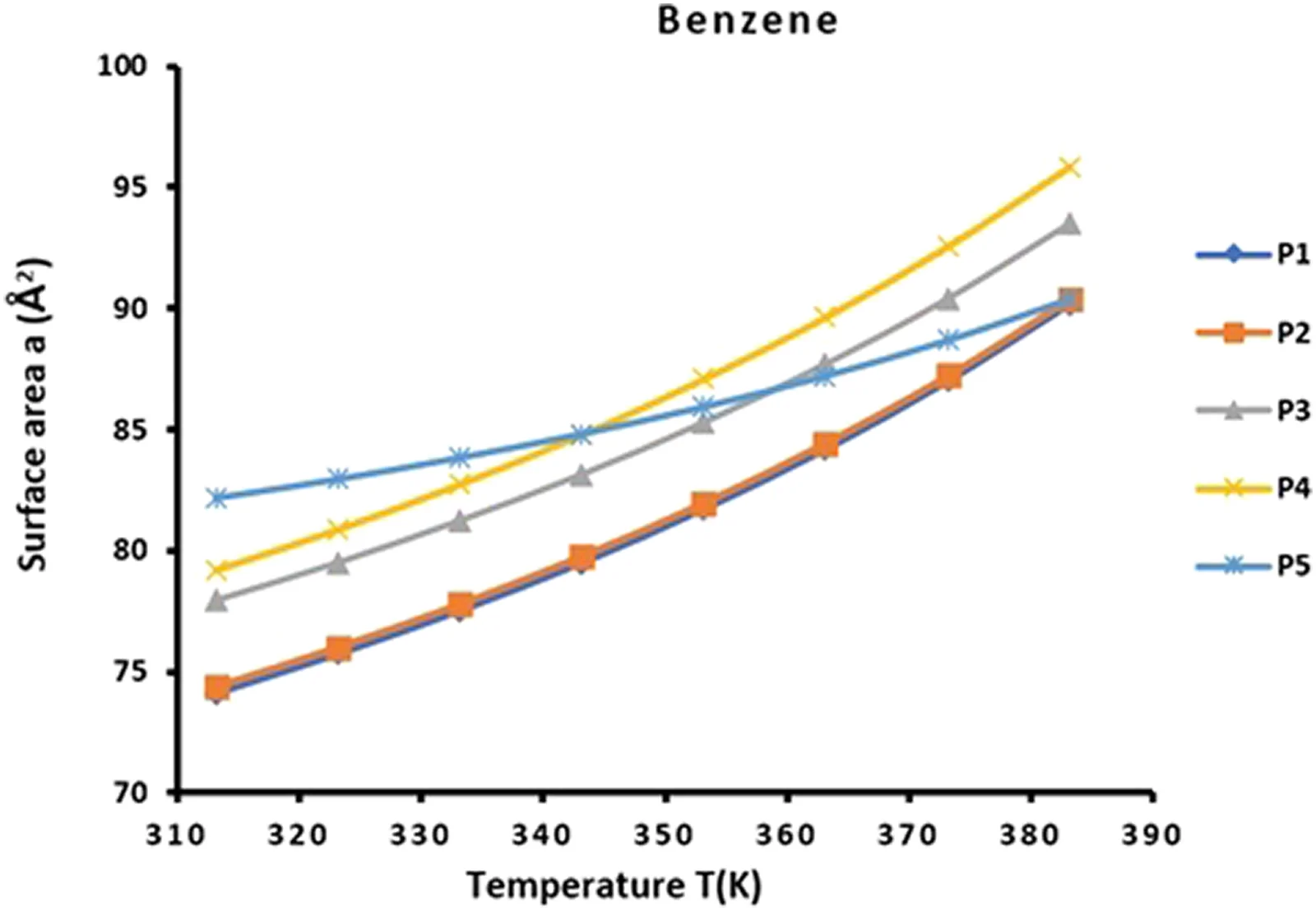
Temperature dependence of the molecular interfacial area, *a*_*X*/*S*_(*T*), for benzene adsorbed on the five DVB-based copolymers P1–P5

**Figure 8:**
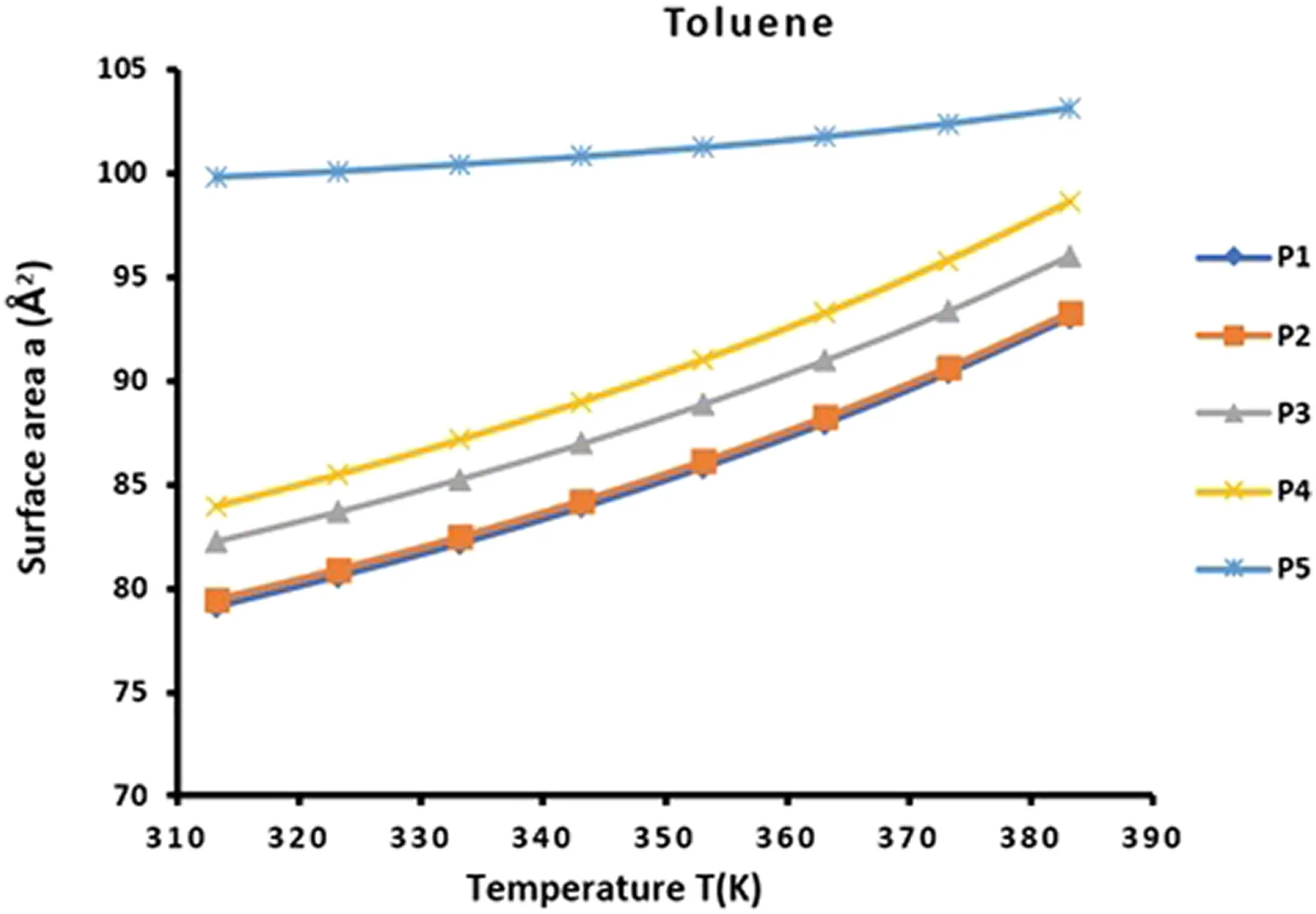
Temperature dependence of the molecular interfacial area, *a*_*X*/*S*_(*T*), for toluene adsorbed on the five DVB-based copolymers P1–P5

**Figure 9:**
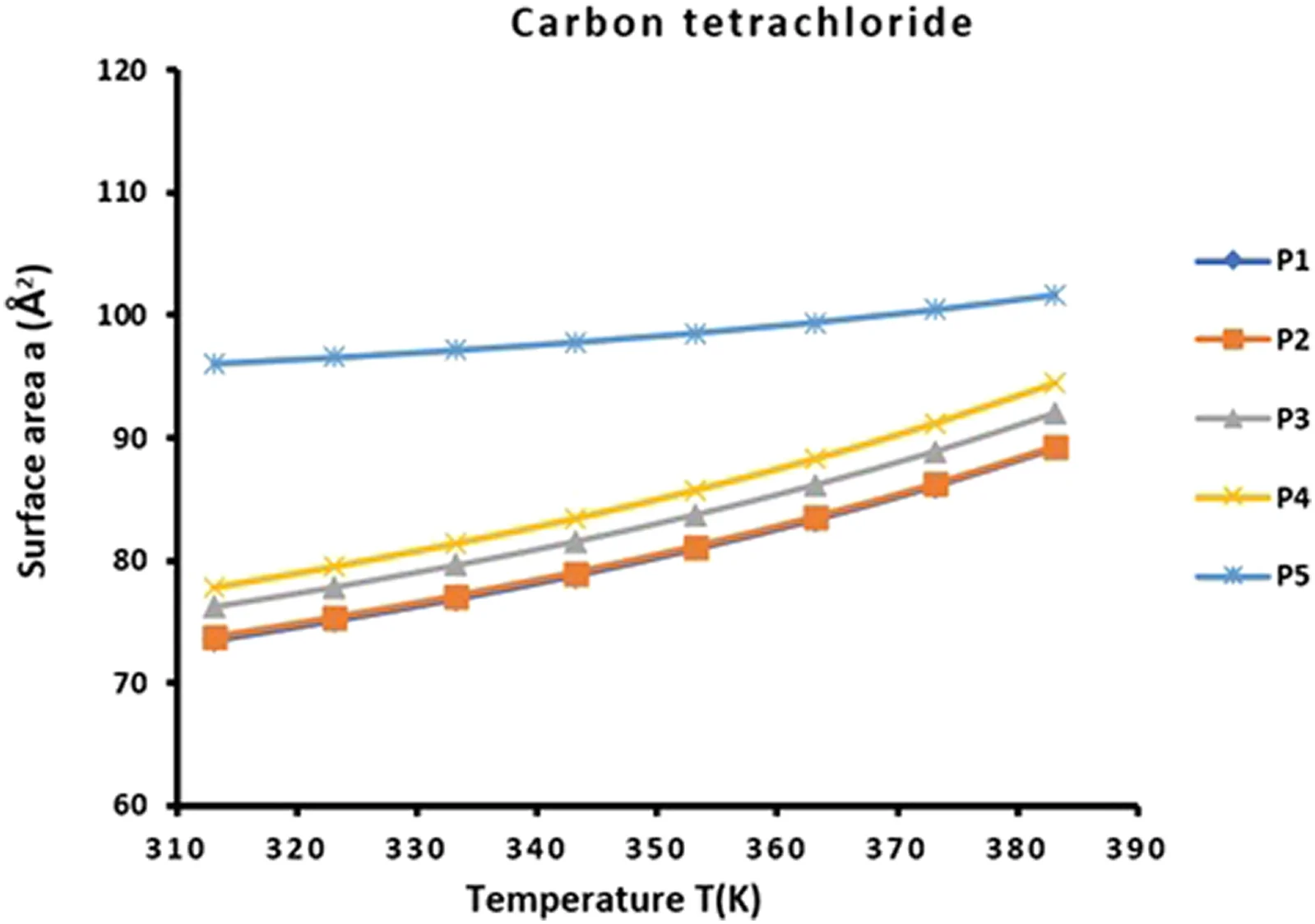
Temperature dependence of the molecular interfacial area, *a*_*X*/*S*_(*T*), for carbon tetrachloride adsorbed on the five DVB-based copolymers P1–P5

**Figure 10:**
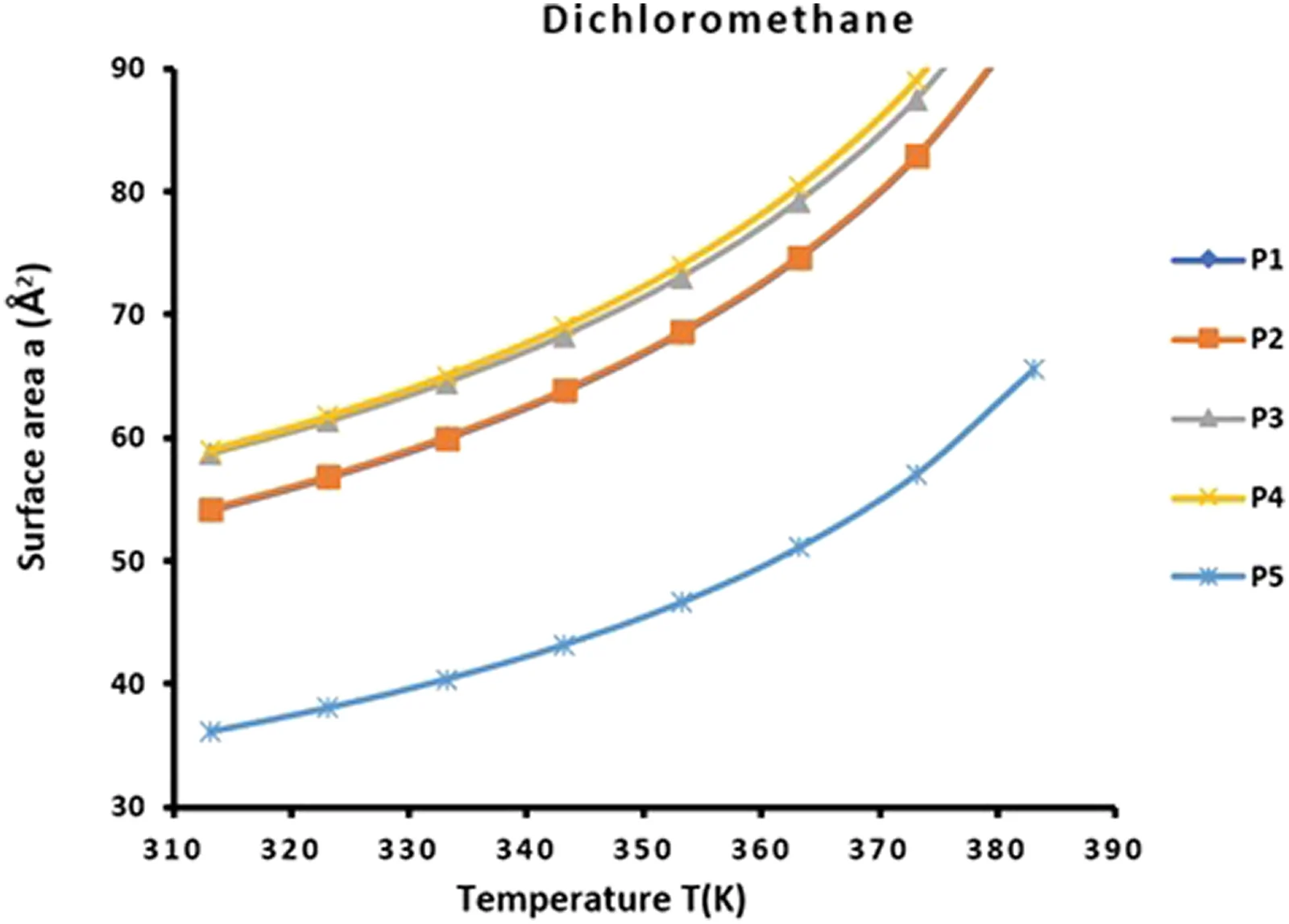
Temperature dependence of the molecular interfacial area, *a*_*X*/*S*_(*T*), for dichloromethane adsorbed on the five DVB-based copolymers P1–P5

**Figure 11:**
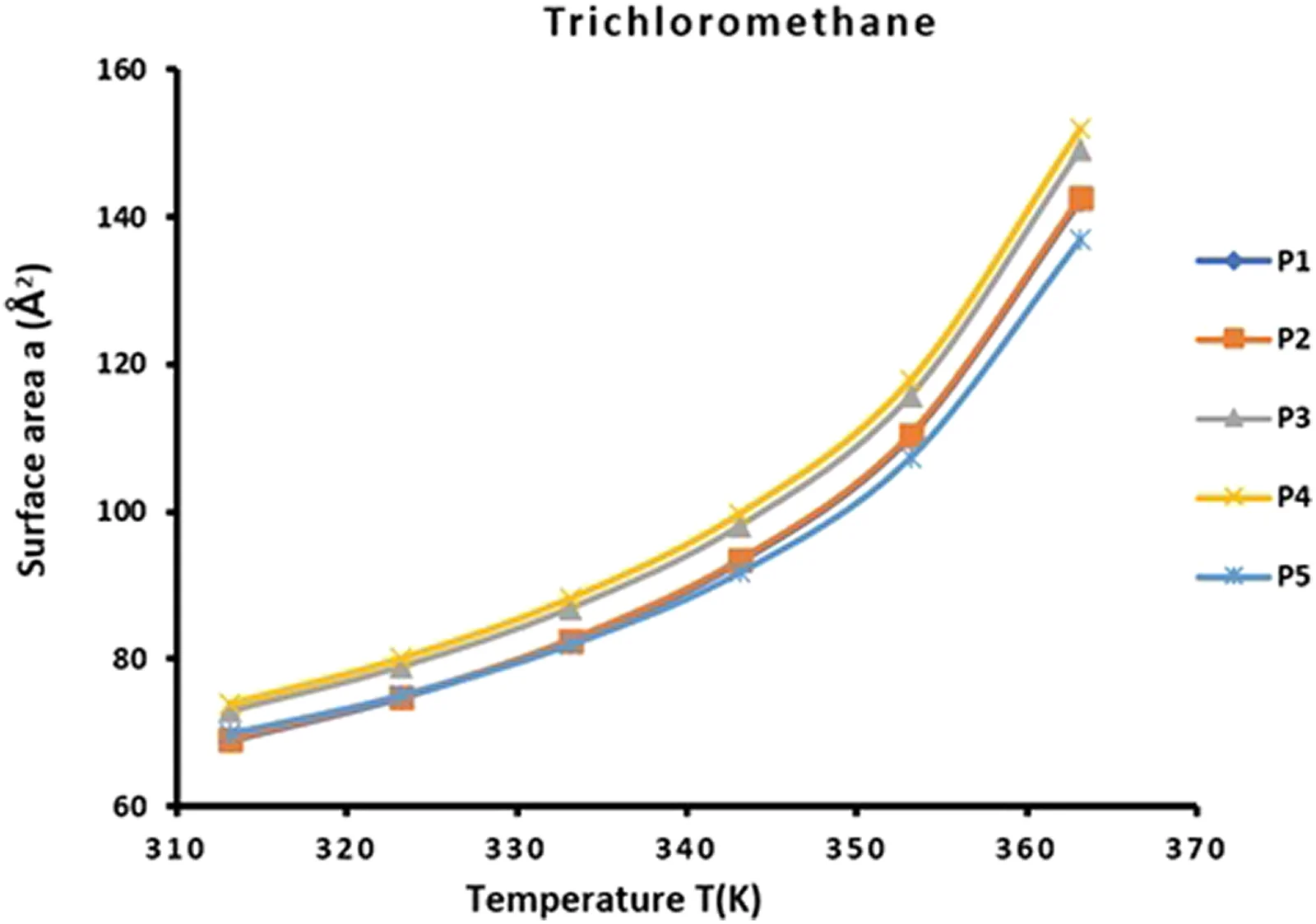
Temperature dependence of the molecular interfacial area, *a*_*X*/*S*_(*T*), for trichloromethane adsorbed on the five DVB-based copolymers P1–P5

**Figure 12:**
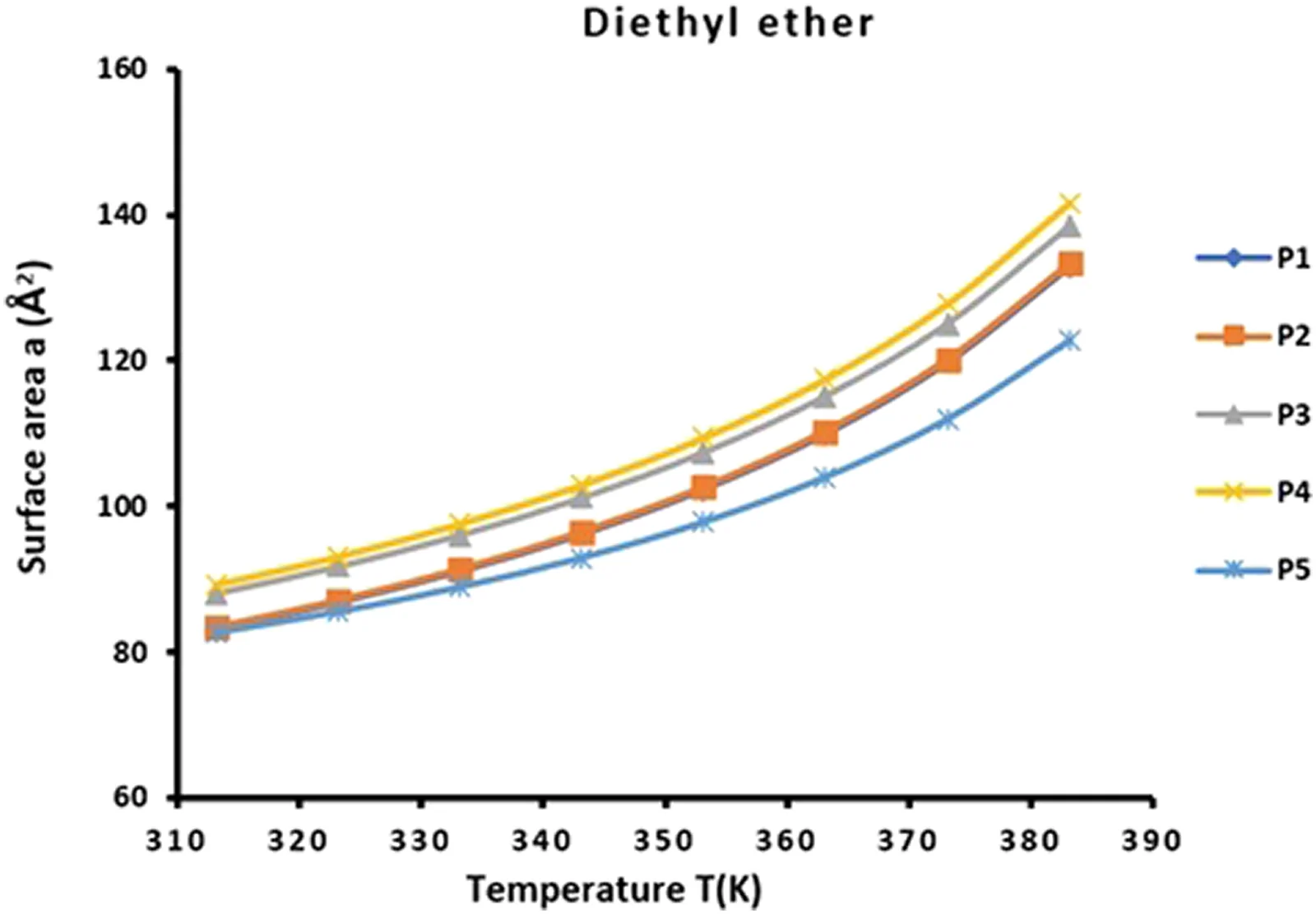
Temperature dependence of the molecular interfacial area, *a*_*X*/*S*_(*T*), for diethyl ether adsorbed on the five DVB-based copolymers P1–P5

**Figure 13:**
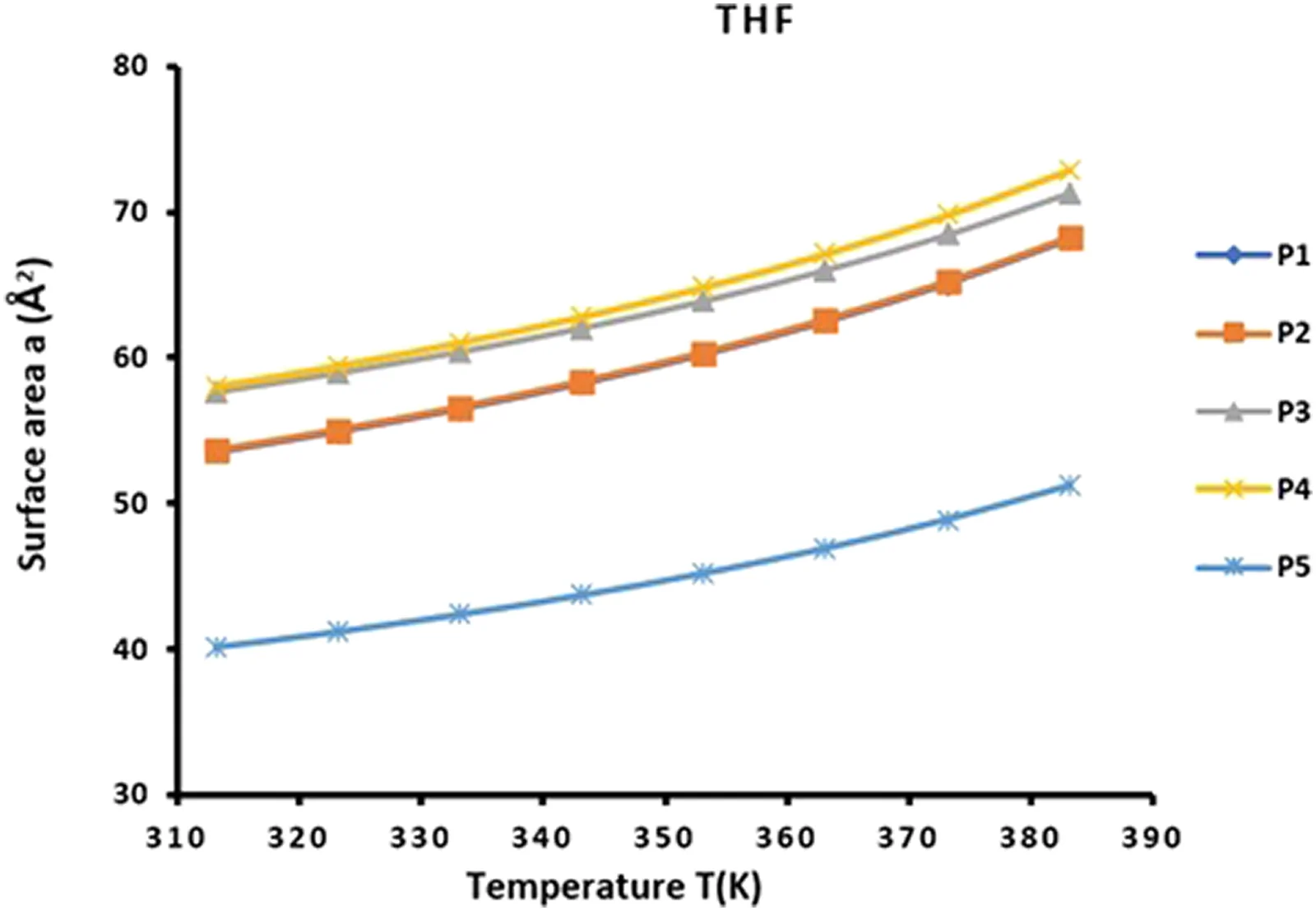
Temperature dependence of the molecular interfacial area, *a*_*X*/*S*_(*T*), for tetrahydrofuran adsorbed on the five DVB-based copolymers P1–P5

**Figure 14:**
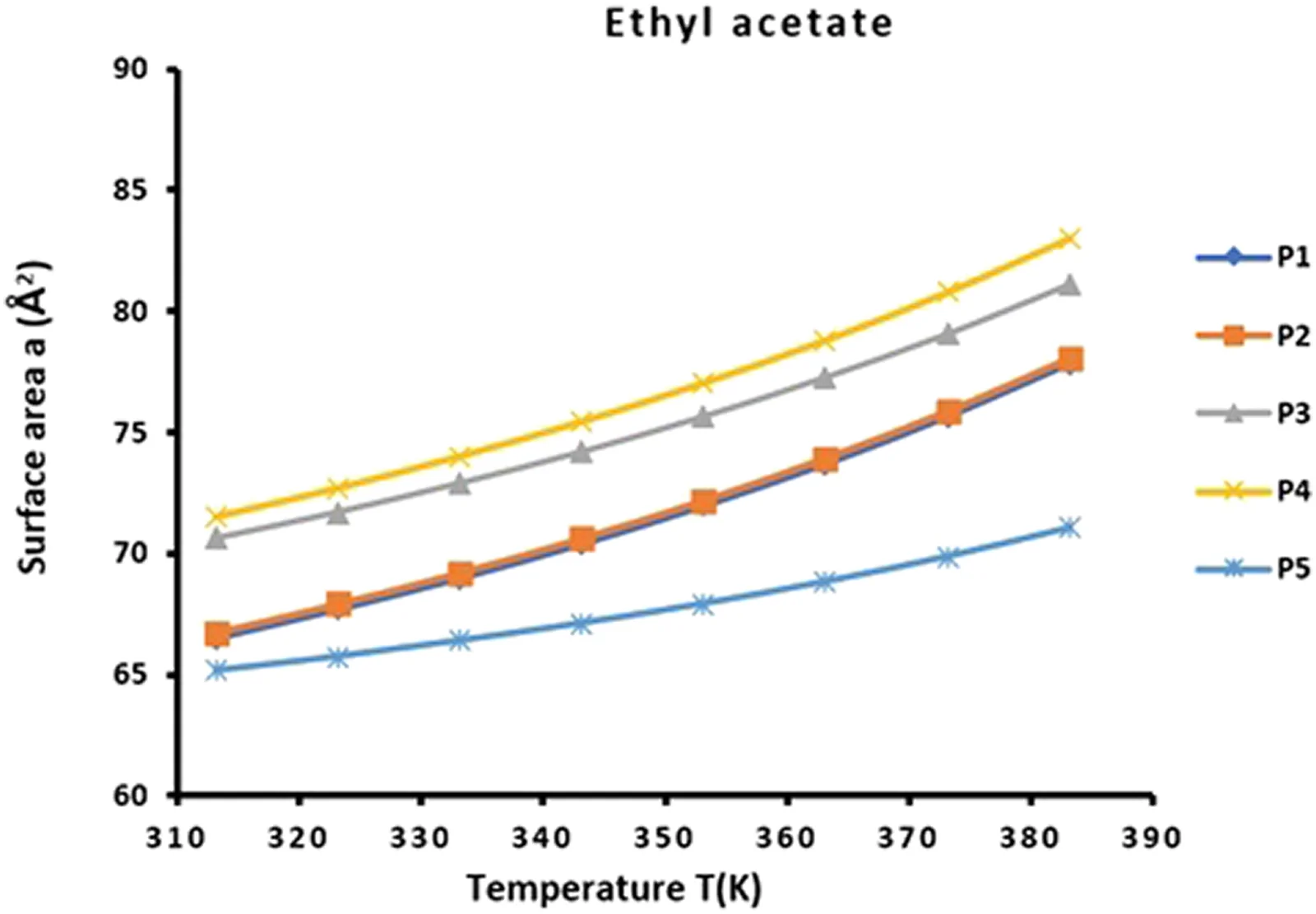
Temperature dependence of the molecular interfacial area, *a*_*X*/*S*_(*T*), for ethyl acetate adsorbed on the five DVB-based copolymers P1–P5

**Figure 15:**
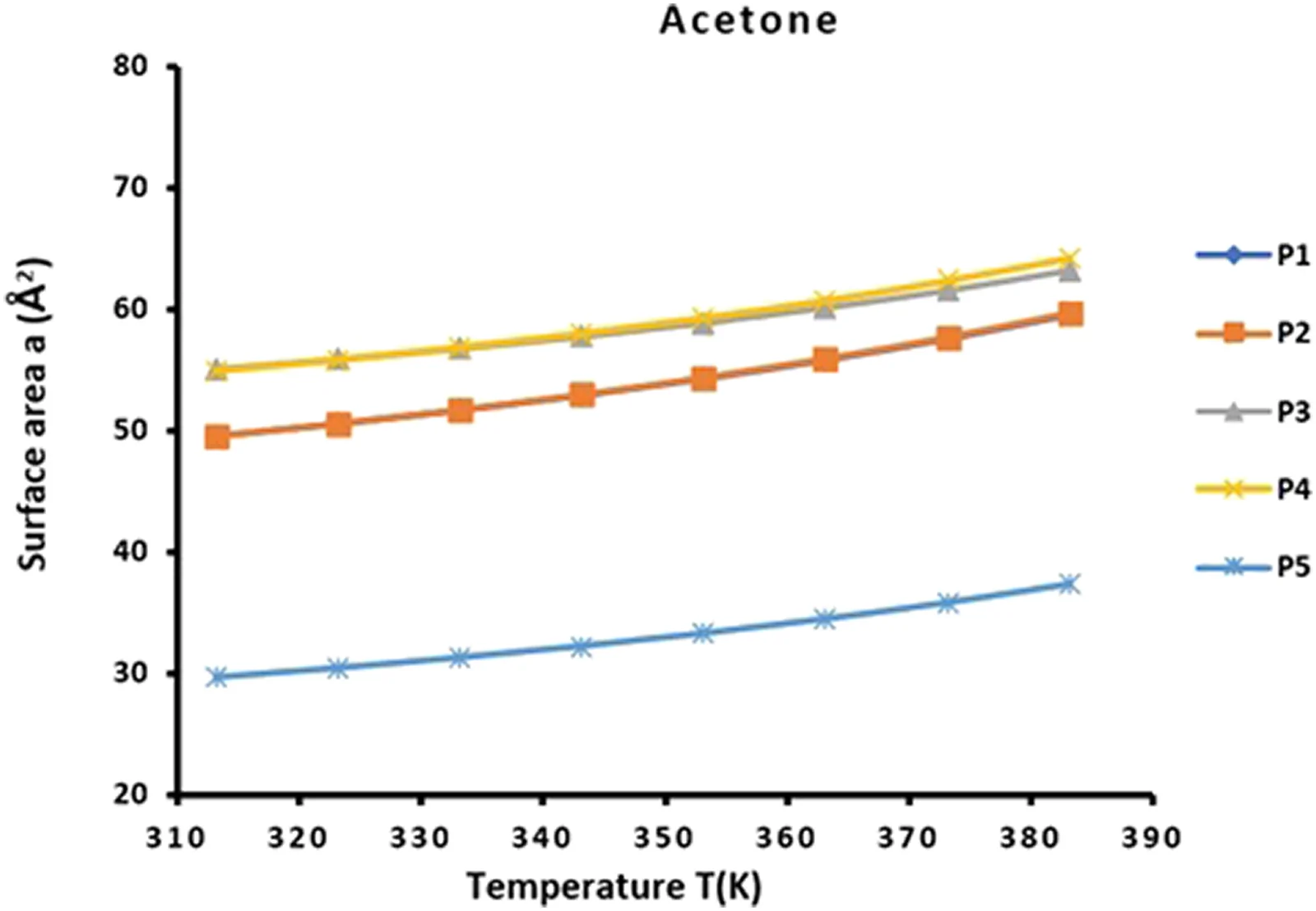
Temperature dependence of the molecular interfacial area, *a*_*X*/*S*_(*T*), for acetone adsorbed on the five DVB-based copolymers P1–P5

**Figure 16:**
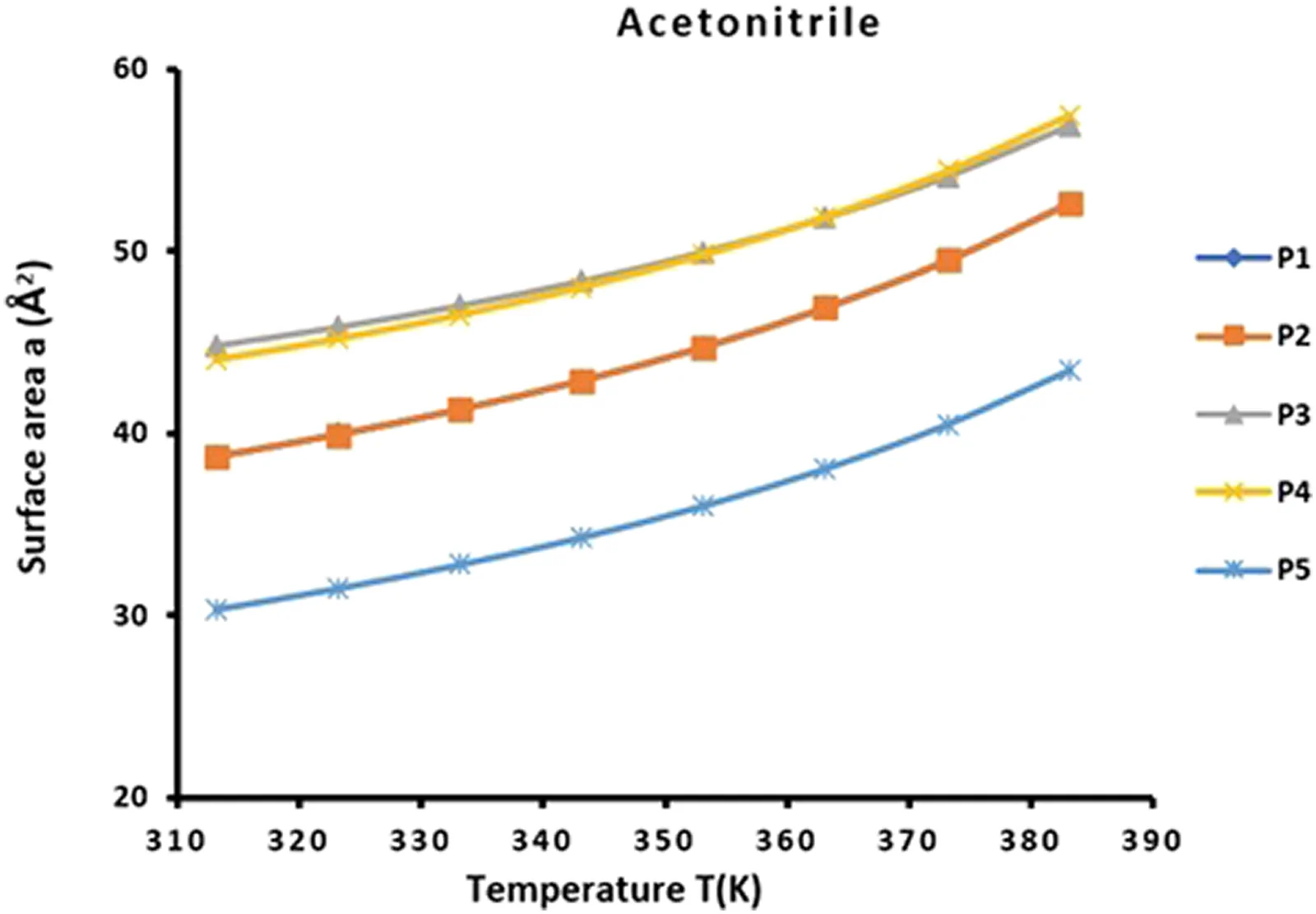
Temperature dependence of the molecular interfacial area, *a*_*X*/*S*_(*T*), for acetonitrile adsorbed on the five DVB-based copolymers P1–P5

**Figure 17:**
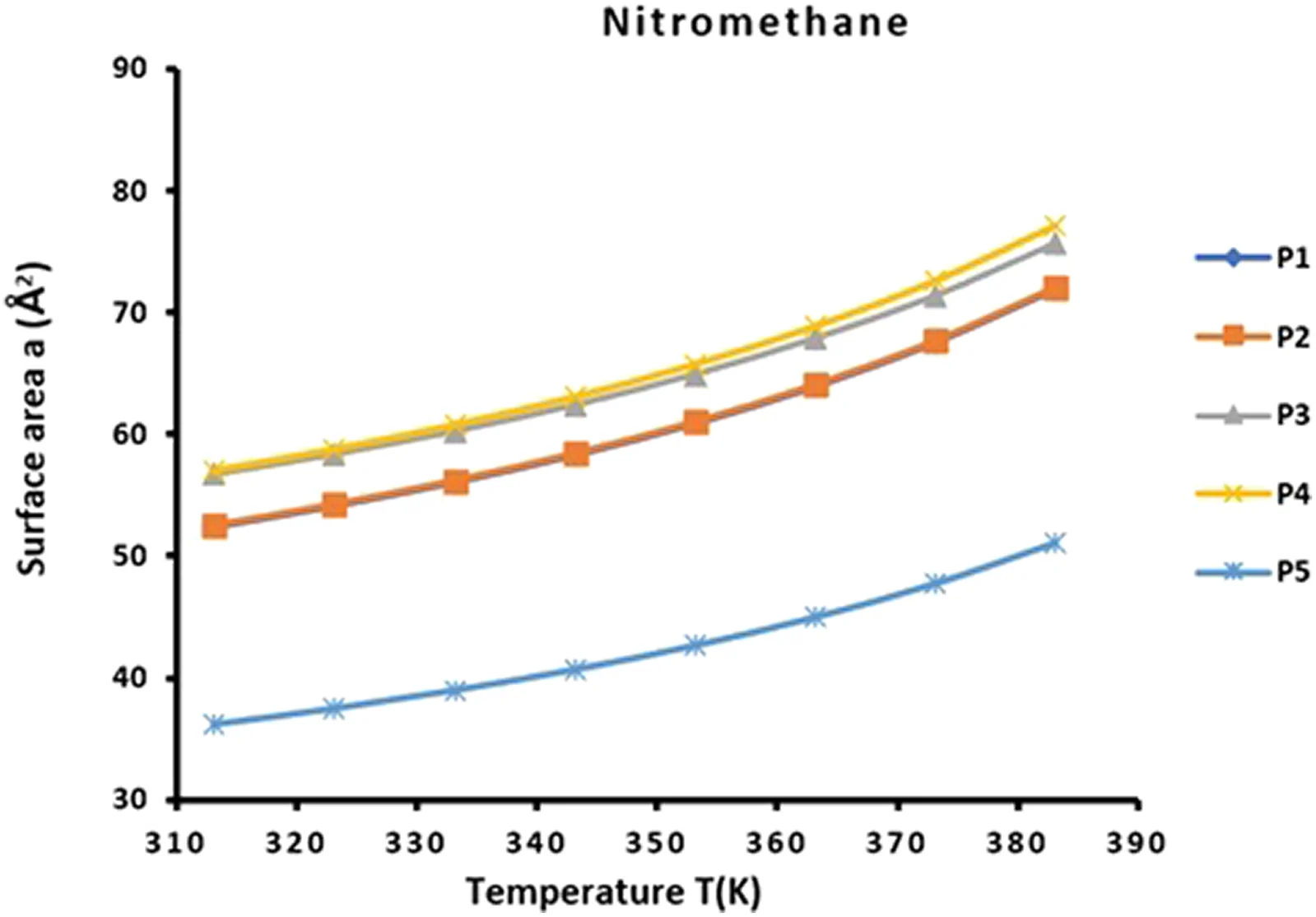
Temperature dependence of the molecular interfacial area, *a*_*X*/*S*_(*T*), for nitromethane adsorbed on the five DVB-based copolymers P1–P5

**Figure 18:**
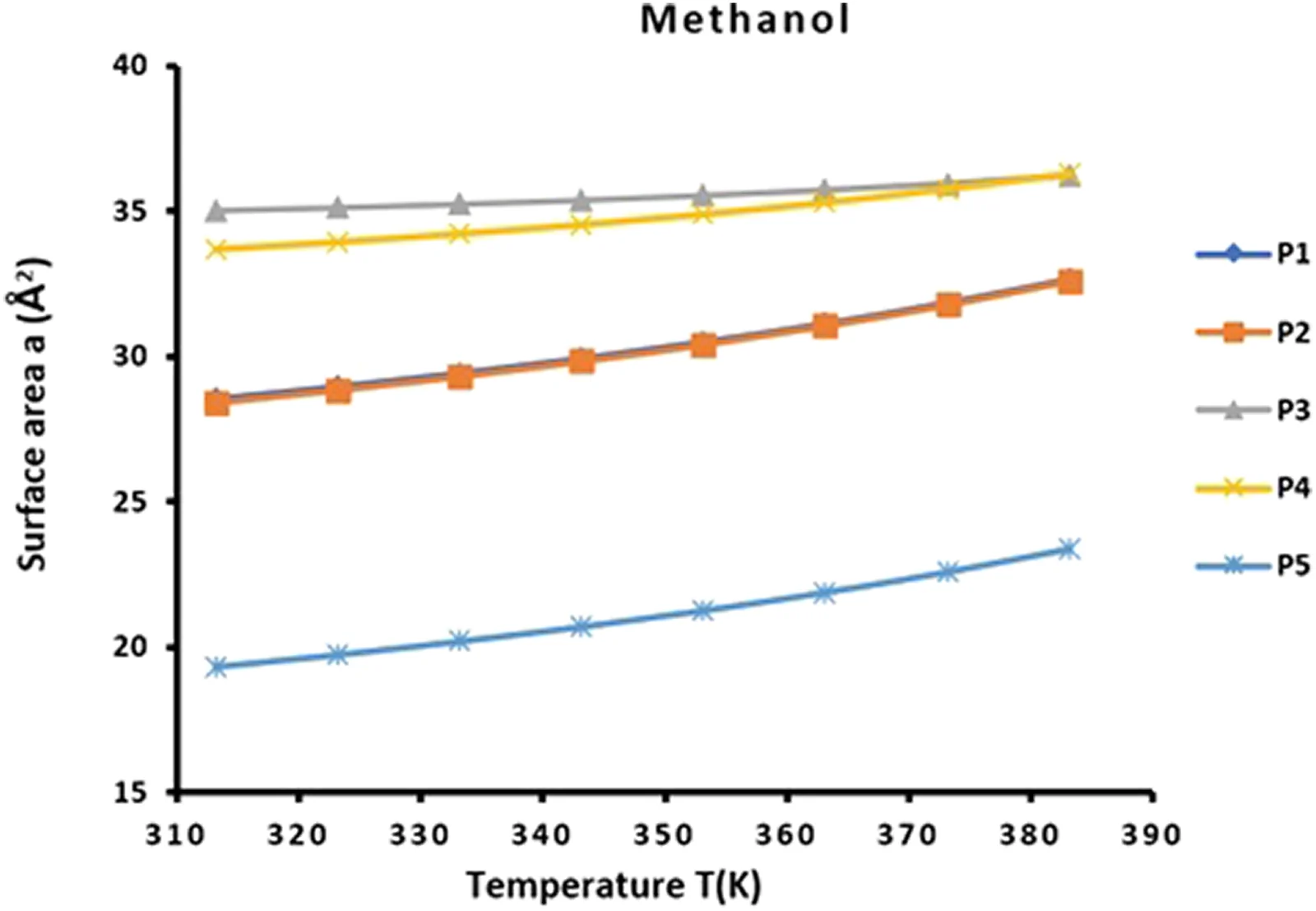
Temperature dependence of the molecular interfacial area, *a*_*X*/*S*_(*T*), for methanol adsorbed on the five DVB-based copolymers P1–P5

**Figure 19:**
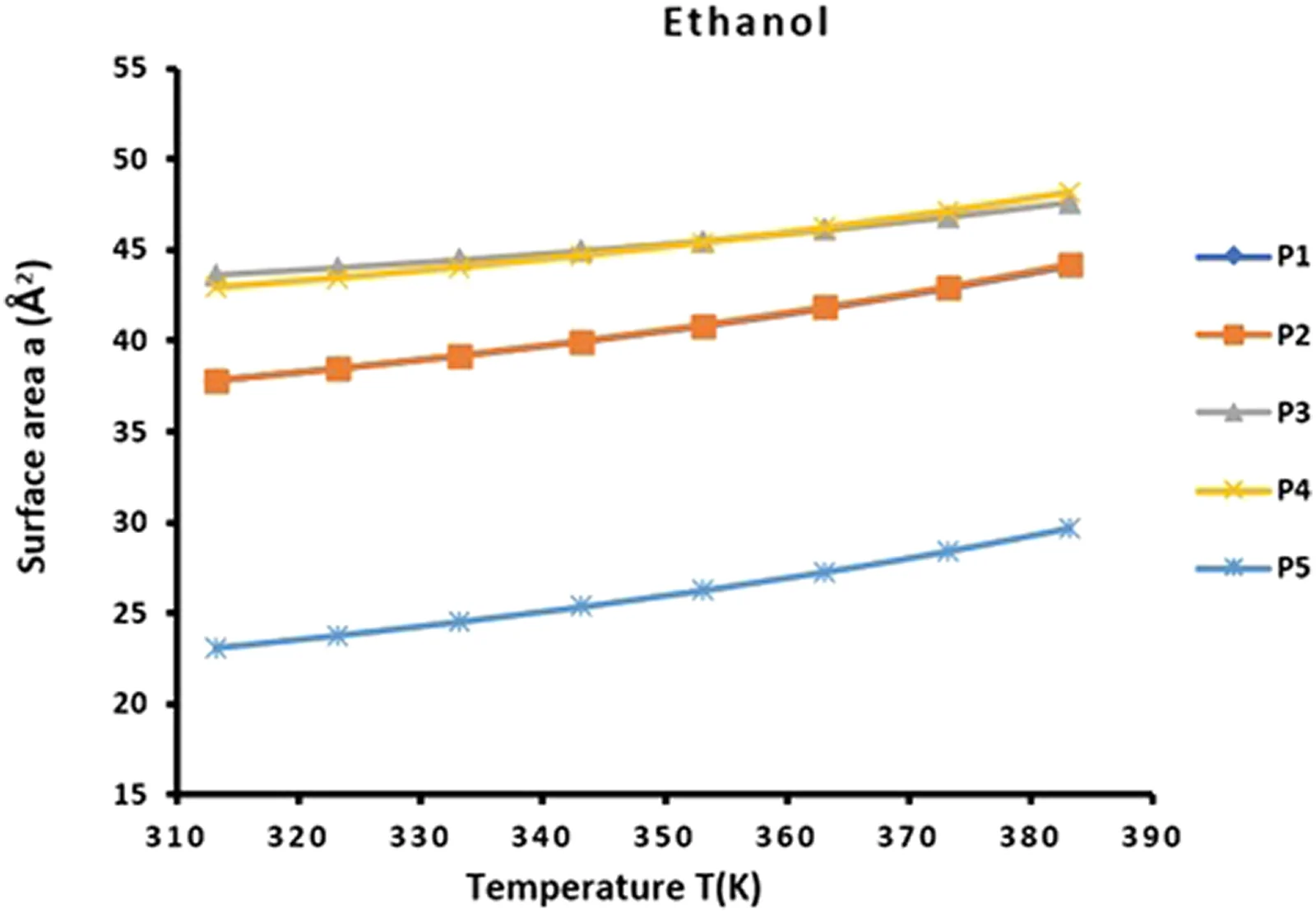
Temperature dependence of the molecular interfacial area, *a*_*X*/*S*_(*T*), for ethanol adsorbed on the five DVB-based copolymers P1–P5

For all investigated solvents and copolymers, *a*_*X*/*S*_(*T*) exhibits a monotonic increase with temperature throughout the investigated interval (313–383 K), as documented by the fitted coefficients summarized in Tables S1A–S1B and the corresponding temperature-dependent profiles presented in Figure S1 (Supplemental Information). The persistence of this trend across chemically diverse probe molecules demonstrates that thermal expansion of the adsorbed phase is a robust and reproducible phenomenon. This behavior supports the interpretation of the adsorbed layer as a thermodynamically organized two-dimensional phase whose effective molecular footprint evolves continuously with temperature. To facilitate interpretation of the copolymer-dependent regimes, a conceptual inset is included in Figures 1, 2, 3, 4, 5, 6, 7, 8, 9, 10, 11, 12, 13, 14, 15, 16, 17, 18, and 19, schematically illustrating how morphology governs both the molecular footprint and the strength of donor-acceptor interactions across the P1-P5 series.

Across all solvents, P1 and P2 exhibit nearly identical behavior, P3 and P4 form an intermediate geometric regime, and P5 displays the strongest deviation, either amplifying or compressing the effective footprint depending on solvent polarity. The persistence of copolymer-specific separation across the entire temperature range demonstrates that adsorption geometry is governed by a morphology-controlled interfacial field rather than purely by chemical composition. Figures 1, 2, 3, 4, 5, 6, 7, 8, 9, 10, 11, 12, 13, 14, 15, 16, 17, 18, and 19 provide direct geometric evidence supporting the unified morphology-thermodynamics framework developed in this work.

As temperature rises, increased vibrational amplitude and reduced residence time within specific adsorption micro-sites enhance lateral configurational freedom, leading to progressive enlargement of the effective projected footprint. The universality of this monotonic expansion across solvent classes confirms that thermal excitation consistently weakens lateral confinement at the interface.

Within each solvent panel, however, the copolymer-dependent separation of the curves reveals a highly structured morphological hierarchy. A first robust observation is the near-superposition of the P1 and P2 curves for essentially all solvents. Across the entire temperature window, the footprints for P1 and P2 differ only marginally, indicating that the DCE and NB synthesis routes do not significantly alter the effective two-dimensional packing environment within this copolymer family. A similar near-overlap is observed for P3 and P4 for many solvents, demonstrating that these two copolymers define a second, internally coherent geometric regime.

More importantly, a systematic separation exists between the groups (P1, P2), P3, and P4, while P5 consistently exhibits the strongest deviation. For nonpolar probes such as n-alkanes, cyclohexane, and aromatics, the hierarchy

(Equation 1) (P1,P2)<P3<P4≪P5

is clearly visible at all temperatures. The persistence of this ordering across the full temperature range indicates that the copolymer effect is not a temperature-specific artifact but reflects a stable morphological field governing adsorption geometry. The fact that the relative separation between copolymers is maintained as the curves shift upward with temperature demonstrates that morphology defines the baseline footprint, while temperature modulates its expansion.

The magnitude of the P5 deviation is particularly striking for long-chain alkanes and cyclic or aromatic molecules, where the effective footprint is significantly larger than for the other copolymers. This suggests that P5 provides an adsorption environment with markedly different lateral packing constraints and accessibility. In contrast, for several small polar molecules (e.g., alcohols, nitriles, ketones), P5 exhibits a reduced or compressed footprint relative to P3 and P4. This polarity-dependent inversion indicates that P5 does not simply rescale adsorption geometry but defines a qualitatively distinct interfacial regime in which molecular orientation, site localization, and packing mode are altered.

For polar probes, the copolymer ranking exhibits a distinct—and mechanistically informative—behavior. With the exception of carbon tetrachloride (predominantly dispersive), cyclohexane and aromatics (also dispersive-dominated), the polar solvents display essentially the same ordering within P1-P4 as observed for nonpolar probes, but poly(DVB) (P5) becomes inverted, yielding:

*P*5<(*P*1,*P*2)<*P*3<*P*4.

This reversal indicates that P5 does not simply shift the footprint magnitude; rather, it imposes a qualitatively different adsorption geometry for polar molecules. In the thermodynamic definition used here, the molecular footprint *a*_*X*/*S*_(*T*) is an *effective projected interfacial area* extracted from adsorption energetics. A smaller *a*_*X*/*S*_ therefore reflects a more compact, localized adsorption state—i.e., adsorption concentrated on fewer, higher-affinity microstates and/or a preferred orientation that reduces the projected area.

A physically consistent interpretation is that, for polar probes, adsorption on P5 is governed by stronger and more specific anchoring (donor-acceptor, induction, and local polarization accommodation) than on the hyper-cross-linked materials. Such anchoring reduces lateral delocalization and favors well-defined orientations (e.g., lone-pair alignment, dipole-field alignment, or site-selective binding), which thermodynamically manifests as a compressed effective footprint despite increasing temperature. In contrast, in the hyper-cross-linked copolymers (P1-P4), polar probes (Figures 6, 7, 8, 9, 10, 11, 12, 13, 14, 15, 16, 17, 18, and 19) are distributed over a broader spectrum of microenvironments—many of which are sterically constrained or energetically moderate—so adsorption is less localized and the extracted footprint remains larger. In other words, the polar probes “sense” P1-P4 primarily as an ensemble of heterogeneous sites, whereas on P5 they preferentially populate a narrower subset of stronger interaction states.

The fact that the inversion is absent for CCl_4_ further supports this mechanism (Figure 9). Carbon tetrachloride, while polarizable, lacks a permanent dipole and classical donor/acceptor character; its adsorption is therefore dominated by dispersive interactions, for which P5 behaves like the nonpolar-probe case (larger footprints). The polarity-dependent switch is therefore not a trivial geometric artifact but a signature of specific interaction control over adsorption geometry.

This interpretation is also consistent with the five-parameter Lewis analysis, where P5 displays markedly enhanced amphoteric strength and stronger nonlinear coupling/curvature terms relative to P1-P4. Stronger specific interactions increase the energetic preference for localized binding configurations, thereby decreasing the effective footprint for polar probes, whereas dispersive probes remain controlled by packing constraints and accessible surface geometry, producing the opposite trend (expanded footprints for P5). Thus, Figures 1, 2, 3, 4, 5, 6, 7, 8, 9, 10, 11, 12, 13, 14, 15, 16, 17, 18, and 19 provide direct geometric evidence that the morphological field is not purely topological: it couples to probe polarity by switching the dominant control from packing-limited dispersive adsorption (nonpolar probes) to site-localized specific adsorption (polar probes).

In this sense, P5 defines a distinct interfacial regime where the adsorption geometry becomes strongly probe-dependent: dispersive-dominated molecules expand into larger projected areas, while polar molecules collapse into compact, orientation-selected microstates. The persistence of this inversion across temperature further indicates that it reflects an intrinsic difference in the distribution of adsorption microstates rather than a temperature-specific effect.

The polarity-dependent inversion of a_X/S_ on P5 therefore demonstrates that interfacial geometry is controlled by a competition between morphology-driven packing constraints and energetics-driven site localization, with probe polarity selecting the dominant mechanism.

The molecular footprint *a*_*X*/*S*_ should be interpreted as an effective thermodynamic projected area derived from adsorption energetics rather than as a direct microscopic measurement of molecular dimensions or adsorption orientation. It represents the average interfacial area effectively occupied by a molecule within the ensemble of adsorption configurations accessible under the investigated thermodynamic conditions. Variations in *a*_*X*/*S*_ therefore reflect changes in lateral packing, confinement, adsorption-site localization, orientational preferences, and configurational entropy rather than a unique molecular geometry. Smaller values of *a*_*X*/*S*_ are generally associated with more localized and compact adsorption states, whereas larger values indicate increased lateral delocalization and greater configurational freedom. Consequently, the molecular footprint provides a thermodynamic descriptor of the geometric organization of the adsorbed phase and its evolution with temperature and surface morphology.

From a thermophysical perspective, these observations demonstrate that the adsorbed molecular footprint is controlled by two coupled variables: temperature and copolymer morphology. Temperature universally drives two-dimensional expansion through entropic activation, while morphology determines the baseline packing state and the amplitude of expansion. The near-parallel character of many curves within each solvent panel suggests that the effective thermal expansion coefficient *λ*_*X*/*S*_:

(Equation 2) $$\lambda_{X/S}={\left(\frac{\partial a_{X/S}}{\partial T}\right)}_{S}$$

remains positive and relatively stable for each copolymer, but its absolute magnitude and initial value are morphology-dependent.

The conceptual inset included in Figures 1, 2, 3, 4, 5, 6, 7, 8, 9, 10, 11, 12, 13, 14, 15, 16, 17, 18, and 19 summarizes the evolution of adsorption geometry across the copolymer series. It illustrates the transition from the nearly overlapping P1-P2 regime to the intermediate P3-P4 state and finally to the distinct P5 morphology, where the adsorption behavior becomes strongly dependent on the nature of the probe molecule. The persistence of these geometric regimes across all investigated temperatures demonstrates that adsorption geometry is governed by an underlying interfacial field associated with the structural organization of the copolymer network rather than by chemical composition alone. Figures 1, 2, 3, 4, 5, 6, 7, 8, 9, 10, 11, 12, 13, 14, 15, 16, 17, 18, and 19 therefore provide direct experimental evidence that surface morphology plays a determining role in defining the accessible adsorption configurations and the resulting molecular packing state.

A fundamentally different behavior is observed for polar probes, including alcohols, nitriles, ketones, ethers, and chlorinated solvents. In these cases, the ordering is generally reversed and follows:

(Equation 3) P5<(P1,P2)<P3<P4

over most of the investigated temperature range. This polarity-dependent inversion indicates that distinct physical mechanisms govern the adsorption geometry of nonpolar and polar molecules. For nonpolar probes, adsorption is dominated by dispersive packing effects. The relatively open morphology of P5 provides greater lateral accessibility and configurational freedom, allowing molecules to occupy a broader range of adsorption configurations and resulting in larger projected molecular footprints. In contrast, adsorption of polar probes is strongly influenced by specific donor-acceptor, induction, and polarization interactions. In this regime, the energetically favorable adsorption sites available on P5 promote orientation-selective anchoring and stronger localization of the adsorbed molecules. Such localization restricts lateral delocalization and produces more compact adsorption configurations, thereby reducing the effective projected footprint. The opposite trends observed for nonpolar and polar probes therefore reveal the dual nature of the P5 interface, where adsorption geometry is controlled either by dispersive packing or by energetic localization depending on the physicochemical character of the probe molecule.

From a thermodynamic perspective, the increase of *a*_*X*/*S*_(*T*) with temperature reflects the competition between enthalpic stabilization at preferred adsorption sites and the entropic tendency toward lateral delocalization within the adsorbed phase. At lower temperatures, adsorption is dominated by energetic minimization, favoring compact and highly localized configurations. As temperature increases, configurational entropy progressively gains importance, allowing molecules to explore a broader range of adsorption microstates and leading to larger projected interfacial areas. The thermal evolution of the molecular footprint therefore constitutes direct geometric evidence of the increasing entropic contribution to the adsorption free energy.

These observations demonstrate that adsorption geometry cannot be regarded as a purely structural parameter. Rather, it behaves as a thermodynamic observable that reflects the coupled evolution of energy and entropy at the interface. In this sense, the molecular footprint provides a geometric counterpart to the energetic information extracted from adsorption thermodynamics. Together, these quantities reveal that adsorption on DVB-based copolymers is governed by a coupled energy-geometry-entropy relationship rather than by independent energetic and structural contributions.

The temperature- and morphology-dependent behavior observed in Figures 1, 2, 3, 4, 5, 6, 7, 8, 9, 10, 11, 12, 13, 14, 15, 16, 17, 18, and 19 therefore establishes the thermophysical foundation of the present study. In the following section, it is shown that the geometric hierarchy revealed by the molecular footprint can be quantitatively described through a universal quadratic relationship $a_{X/S}\left(T,S\right)$ involving both temperature and SSA. Subsequently, it will be demonstrated that the same hierarchical organization governs the nonlinear donor-acceptor energetics described by the five-parameter Lewis model. The convergence of these independent geometric and energetic descriptors ultimately leads to a unified morphology-coupled thermodynamic framework in which adsorption geometry, interaction strength, and configurational entropy emerge as interconnected manifestations of the same underlying interfacial field.

### Adsorption geometry follows a common thermo-geometric relationship

#### Molecular footprints exhibit a quadratic dependence on temperature and surface morphology

The systematic thermal expansion and morphology-dependent separation previously observed naturally lead to a more general thermodynamic description of the adsorbed molecular footprint. Two key experimental observations require formalization: (1) the monotonic increase of *a*_*X*/*S*_(*T*) for every copolymer and solvent, and (2) the persistent copolymer hierarchy across the entire temperature range. Together, these features imply that the footprint must be expressed as a coupled function of both temperature and SSA.

The systematic temperature-morphology hierarchy established in Section 3.1 suggests that the molecular interfacial area *a*_*X*/*S*_(*T*) is not merely a monotonic function of temperature, but the manifestation of a deeper thermo-geometric coupling governed jointly by temperature and SSA S. To quantify this behavior, the full dataset for all solvents and copolymers was analyzed using polynomial expansions in both variables.

The observed quadratic dependence of *a*_*X*/*S*_(*T*,*S*) is described as universal only within the investigated family of DVB- and CDVB-based copolymers and within the explored temperature interval (313–383 K) and specific-surface-area domain. The remarkable convergence of all investigated solvent systems toward the same mathematical form suggests the existence of a morphology-controlled thermo-geometric principle within this copolymer family. However, extension of this behavior to other classes of polymers, porous materials, or broader thermodynamic conditions requires independent experimental verification.

Table S2 presents the linear temperature dependence of the molecular interfacial area and the corresponding thermal expansion coefficients *λ*_*X*/*S*_ for all systems. The strong linear correlations (*R*^2^ > 0.95) confirm that the adsorbed layer exhibits a well-defined two-dimensional thermal expansion behavior within the investigated temperature range. The parameter *λ*_*X*/*S*_ represents the effective 2D expansion coefficient of the adsorbed phase and quantifies the sensitivity of the molecular footprint to thermal excitation. The variation of *λ*_*X*/*S*_ among the investigated copolymers reflects significant differences in confinement effects, energetic heterogeneity, adsorption-site accessibility, and the effective stiffness of the adsorption potential. The fitted parameters reported in Tables S3 and S4 (Supplementary Information) demonstrate that both the baseline molecular footprint and the corresponding thermal expansion coefficient vary systematically with SSA. This behavior indicates that surface morphology influences not only the initial packing state of the adsorbed molecules but also their ability to undergo thermally induced lateral expansion. Consequently, the thermo-geometric response of the adsorbed phase is governed by a coupled morphology-temperature dependence, in which SSA modulates both the magnitude of the molecular footprint and its thermal evolution. The linear and quadratic regressions confirm that morphology modulates the thermal responsiveness of the adsorbed layer. The non-zero dependence of *λ*_*X*/*S*_ on S implies that temperature and surface topology are thermodynamically coupled variables. Increasing S modifies confinement and lateral packing constraints, thereby altering the magnitude of thermal expansion. The improved quadratic fits further support the presence of nonlinear morphology-temperature interactions.

The quadratic temperature dependence of the molecular interfacial area *a*_*X*/*S*_(*T*) was summarized in Table 1 for all investigated solvents and copolymer systems. The coefficients *a*_2_, *a*_1_, and *a*_0_ describe the curvature, linear thermal contribution, and baseline footprint at the reference temperature (*T*_0_ = 298.15 K), respectively. The consistently high regression coefficients confirm that the quadratic formulation provides an excellent representation of the thermophysical behavior of the adsorbed layer.

**Table 1:** Quadratic coefficients describing the temperature dependence of the adsorbed molecular footprint for solvents adsorbed on DVB-based copolymers

T(K)	P1					
Solvents	Equations *a*_*X*/*S*_ (*T*)	*a*_2_	*a*_1_	*a*_0_	*a*_*X*/*S*_(*T*_0_)	R^2^
n-Pentane	a(T) = 0.0056T^2^ - 3.3022T + 571.7871	0.0056	3.3022	571.7871	85.03	0.99883
n-Hexane	a(T) = 0.0031T^2^ - 1.7130T + 326.6196	0.0031	1.7130	326.6196	89.67	0.99963
n-Heptane	a(T) = 0.0023T^2^ - 1.2516T + 261.9781	0.0023	1.2516	261.9781	96.82	0.99979
n-Octane	a(T) = 0.0020T^2^ - 1.0456T + 237.5586	0.0020	1.0456	237.5586	104.48	0.99985
n-Nonane	a(T) = 0.0018T^2^ - 0.9389T + 228.4593	0.0018	0.9390	228.4593	112.07	0.99988
CCl_4_	a(T) = 0.0012T^2^ - 0.6336T + 151.4802	0.0012	0.6336	151.4802	71.93	0.99986
Nitromethane	a(T) = 0.0022T^2^ - 1.2279T + 225.4574	0.0022	1.2279	225.4574	51.37	0.99943
CH_2_Cl_2_	a(T) = 0.0069T^2^ - 4.2560T + 710.8753	0.0069	4.2560	710.8753	55.32	0.99611
CHCl_3_	a(T) = 0.0301T^2^ - 18.9896T + 3062.889	0.0301	18.9896	3062.8891	79.51	0.99105
Diethyl ether	a(T) = 0.0074T^2^ - 4.4352T + 751.2761	0.0074	4.4352	751.2761	83.19	0.99797
THF	a(T) = 0.0013T^2^ - 0.7209T + 148.8041	0.0013	0.7209	148.8041	52.10	0.99975
Ethyl acetate	a(T) = 0.0008T^2^ - 0.4174T + 115.8558	0.0008	0.4174	115.8558	65.20	0.99989
Acetone	a(T) = 0.0008T^2^ - 0.4178T + 101.6892	0.0008	0.4178	101.6892	48.22	0.99985
Acetonitrile	a(T) = 0.0016T^2^ - 0.8913T + 164.9803	0.0016	0.8913	164.9803	37.90	0.9994
Toluene	a(T) = 0.0010T^2^ - 0.5084T + 139.0801	0.0010	0.5084	139.0801	77.27	0.99989
Benzene	a(T) = 0.0013T^2^ - 0.6646T + 156.7341	0.0013	0.6646	156.7341	72.36	0.99985
Methanol	a(T) = 0.0003T^2^ - 0.1636T + 48.5628	0.0003	0.1636	48.5628	28.23	0.99987
Ethanol	a(T) = 0.0005T^2^ - 0.2488T + 68.1849	0.0005	0.2488	68.1849	37.56	0.99987
Cyclohexane	a(T) = 0.0014T^2^ - 0.7201T + 164.3595	0.0014	0.7201	164.3595	72.34	0.99984

**T(K)**	**P2**					

**Solvents**	**Equations *a***_***X*/*S***_**(*T*)**	***a***_**2**_	***a***_**1**_	***a***_**0**_	***a***_***X*/*S***_**(*T***_**0**_**)**	**R**^**2**^

n-Pentane	a(T) = 0.0056T^2^ - 3.3081T + 573.0377	0.0056	3.3081	573.0377	85.43	0.99883
n-Hexane	a(T) = 0.0031T^2^ - 1.7097T + 326.5723	0.0031	1.7097	326.5723	90.63	0.99962
n-Heptane	a(T) = 0.0023T^2^ - 1.2501T + 262.3714	0.0023	1.2501	262.3714	97.65	0.99979
n-Octane	a(T) = 0.0020T^2^ - 1.0407T + 237.5623	0.0020	1.0408	237.5623	105.04	0.99985
n-Nonane	a(T) = 0.0018T^2^ - 0.9355T + 228.8082	0.0018	0.9355	228.8082	112.58	0.99987
CCl_4_	a(T) = 0.0012T^2^ - 0.6258T + 150.5621	0.0012	0.6258	150.5621	72.43	0.99987
Nitromethane	a(T) = 0.0022T^2^ - 1.2250T + 225.0281	0.0022	1.2250	225.0281	50.93	0.99944
CH_2_Cl_2_	a(T) = 0.0069T^2^ - 4.2575T + 711.1472	0.0069	4.2575	711.1472	56.02	0.99612
CHCl_3_	a(T) = 0.0302T^2^ - 19.0395T + 3071.0526	0.0302	19.0395	3071.0526	79.90	0.99102
Diethyl ether	a(T) = 0.0074T^2^ - 4.4333T + 751.1261	0.0074	4.4333	751.1261	83.61	0.99798
THF	a(T) = 0.0013T^2^ - 0.7172T + 148.2786	0.0013	0.7172	148.2786	52.69	0.99976
Ethyl acetate	a(T) = 0.0008T^2^ - 0.4116T + 115.1182	0.0008	0.4116	115.1182	65.31	0.9999
Acetone	a(T) = 0.0008T^2^ - 0.4153T + 101.2450	0.0008	0.4153	101.2450	48.54	0.99986
Acetonitrile	a(T) = 0.0016T^2^ - 0.8915T + 164.8652	0.0016	0.8915	164.8652	37.73	0.99941
Toluene	a(T) = 0.0010T^2^ - 0.5005T + 138.1681	0.0010	0.5005	138.1681	77.84	0.9999
Benzene	a(T) = 0.0013T^2^ - 0.6577T + 155.8792	0.0013	0.6577	155.8792	72.69	0.99986
Methanol	a(T) = 0.0003T^2^ - 0.1642T + 48.4039	0.0003	0.1642	48.4039	27.89	0.99988
Ethanol	a(T) = 0.0005T^2^ - 0.2478T + 67.8848	0.0005	0.2478	67.8848	37.55	0.99988
Cyclohexane	a(T) = 0.0014T^2^ - 0.7125T + 163.4532	0.0014	0.7125	163.4532	72.82	0.99985

**T(K)**	**P3**					

**Solvents**	**Equations *a***_***X*/*S***_**(*T*)**	***a***_**2**_	***a***_**1**_	***a***_**0**_	***a***_***X*/*S***_**(*T***_**0**_**)**	**R**^**2**^

n-Pentane	a(T) = 0.0057T^2^ - 3.3584T + 584.9404	0.0057	3.3584	584.9404	89.45	0.99881
n-Hexane	a(T) = 0.0032T^2^ - 1.7478T + 334.5660	0.0032	1.7478	334.5660	93.47	0.99962
n-Heptane	a(T) = 0.0024T^2^ - 1.2998T + 270.2366	0.0024	1.2998	270.2366	98.71	0.99979
n-Octane	a(T) = 0.0021T^2^ - 1.1034T + 245.5784	0.0021	1.1034	245.5784	105.04	0.99985
n-Nonane	a(T) = 0.0020T^2^ - 1.0107T + 236.9559	0.0020	1.0107	236.9559	112.52	0.99988
CCl_4_	a(T) = 0.0012T^2^ - 0.6433T + 155.7269	0.0012	0.6433	155.7266	74.14	0.99987
Nitromethane	a(T) = 0.0021T^2^ - 1.2049T + 227.3167	0.0021	1.2049	227.3167	55.65	0.99941
CH_2_Cl_2_	a(T) = 0.0070T^2^ - 4.337413T + 729.1253	0.0070	4.3374	729.1253	59.96	0.99598
CHCl_3_	a(T) = 0.0313T^2^ - 19.7605T + 3189.6236	0.0313	19.7605	3189.6236	83.95	0.99094
Diethyl ether	a(T) = 0.0075T^2^ - 4.5075T + 768.0140	0.0075	4.5075	768.0140	88.13	0.99793
THF	a(T) = 0.0013T^2^ - 0.6903T + 149.5548	0.0013	0.6903	149.5548	56.63	0.99974
Ethyl acetate	a(T) = 0.0008T^2^ - 0.3974T + 118.2427	0.0008	0.3974	118.2427	69.09	0.99989
Acetone	a(T) = 0.0007T^2^ - 0.3597T + 100.9770	0.0007	0.3597	100.9770	54.17	0.999
Acetonitrile	a(T) = 0.0014T^2^ - 0.8150T + 161.4961	0.0014	0.8150	161.4961	43.86	0.99933
Toluene	a(T) = 0.0010T^2^ - 0.5090T + 142.6316	0.0010	0.5090	142.6316	80.66	0.9999
Benzene	a(T) = 0.0013T^2^ - 0.6544T + 159.7392	0.0013	0.6544	159.7392	76.62	0.99985
Methanol	a(T) = 0.0001T^2^ - 0.0801T + 46.4212	0.0001	0.0801	46.4212	35.00	0.99964
Ethanol	a(T) = 0.0003T^2^ - 0.1813T + 67.0167	0.0003	0.1813	67.0167	43.19	0.99984
Cyclohexane	a(T) = 0.0014T^2^ - 0.7273T + 168.4559	0.0014	0.7273	168.4556	75.16	0.99984

**T(K)**	**P4**					

**Solvents**	**Equations *a***_***X*/*S***_**(*T*)**	***a***_**2**_	***a***_**1**_	***a***_**0**_	***a***_***X*/*S***_**(*T***_**0**_**)**	**R**^**2**^

n-Pentane	a(T) = 0.0059T^2^ - 3.4892T + 605.3020	0.0059	3.4892	605.3020	91.25	0.99882
n-Hexane	a(T) = 0.0033T^2^ - 1.8172T + 345.9979	0.0033	1.8172	345.9979	94.88	0.99963
n-Heptane	a(T) = 0.0025T^2^ - 1.3457T + 278.8424	0.0025	1.3457	278.8424	101.62	0.99979
n-Octane	a(T) = 0.0022T^2^ - 1.1360T + 252.9356	0.0022	1.1360	252.9356	108.91	0.99985
n-Nonane	a(T) = 0.0020T^2^ - 1.0343T + 243.7425	0.0020	1.0343	243.7425	115.81	0.99988
CCl_4_	a(T) = 0.0013T^2^ - 0.6714T + 160.4325	0.0013	0.6714	160.4325	75.82	0.99987
Nitromethane	a(T) = 0.0022T^2^ - 1.2711T + 236.5115	0.0022	1.2711	236.5115	55.78	0.99943
CH_2_Cl_2_	a(T) = 0.0073T^2^ - 4.4924T + 752.7379	0.0073	4.4924	752.7379	60.48	0.99605
CHCl_3_	a(T) = 0.0321T^2^ - 20.2305T + 3263.9856	0.0321	20.2305	3263.9856	84.86	0.99103
Diethyl ether	a(T) = 0.0078T^2^ - 4.6761T + 794.0046	0.0078	4.6761	794.0046	88.74	0.99796
THF	a(T) = 0.0014T^2^ - 0.7371T + 155.7497	0.0014	0.7371	155.7497	56.88	0.99975
Ethyl acetate	a(T) = 0.0009T^2^ - 0.4265T + 122.0777	0.0009	0.4265	122.0777	70.46	0.9999
Acetone	a(T) = 0.0008T^2^ - 0.4031T + 105.6330	0.0008	0.4031	105.6330	53.89	0.99985
Acetonitrile	a(T) = 0.0015T^2^ - 0.8864T + 170.2409	0.0015	0.8864	170.2409	42.86	0.99937
Toluene	a(T) = 0.0011T^2^ - 0.5349T + 146.9268	0.0011	0.5349	146.9268	82.56	0.9999
Benzene	a(T) = 0.0013T^2^ - 0.6917T + 165.2054	0.0013	0.6917	165.2054	77.21	0.99986
Methanol	a(T) = 0.0002T^2^ - 0.1206T + 49.3922	0.0002	0.1206	49.3922	33.88	0.99983
Ethanol	a(T) = 0.0004T^2^ - 0.2200T + 70.4050	0.0004	0.2200	70.4050	42.16	0.99986
Cyclohexane	a(T) = 0.0015T^2^ - 0.7604T + 173.8316	0.0015	0.7604	173.8316	76.90	0.99985

**T(K)**	**P5**					

**Solvents**	**Equations *a***_***X*/*S***_**(*T*)**	***a***_**2**_	***a***_**1**_	***a***_**0**_	***a***_***X*/*S***_**(*T***_**0**_**)**	**R**^**2**^

n-Pentane	a(T) = 0.0047T^2^ - 2.8023T + 514.8566	0.0047	2.8023	514.8566	98.05	0.99862
n-Hexane	a(T) = 0.0022T^2^ - 1.2582T + 303.8227	0.0022	1.2582	303.8227	124.27	0.99947
n-Heptane	a(T) = 0.0013T^2^ - 0.7171T + 249.4653	0.0013	0.7171	249.4653	147.66	0.99958
n-Octane	a(T) = 0.0007T^2^ - 0.4285T + 245.5575	0.0007	0.4285	245.5575	180.91	0.99924
n-Nonane	a(T) = 0.0003T^2^ - 0.2404T + 241.0106	0.0003	0.2404	241.0106	199.56	0.98045
CCl_4_	a(T) = 0.0006T^2^ - 0.3184T + 139.9121	0.0006	0.3184	139.9121	95.66	0.99968
Nitromethane	a(T) = 0.0016T^2^ - 0.9319T + 167.4910	0.0016	0.9319	167.4910	35.42	0.99943
CH_2_Cl_2_	a(T) = 0.0050T^2^ - 3.0703T + 509.3967	0.0050	3.0703	509.3967	36.66	0.99619
CHCl_3_	a(T) = 0.0280T^2^ - 17.6713T + 2860.105	0.0280	17.6713	2860.1046	80.41	0.9906
Diethyl ether	a(T) = 0.0061T^2^ - 3.7052T + 644.069	0.0061	3.7052	644.0688	82.50	0.99775
THF	a(T) = 0.0010T^2^ - 0.5546T + 113.8301	0.0010	0.5546	113.8301	39.14	0.99973
Ethyl Acetate	a(T) = 0.0005T^2^ - 0.2595T + 98.1784	0.0005	0.2595	98.1784	64.38	0.99982
Acetone	a(T) = 0.0006T^2^ - 0.3226T + 69.8516	0.0006	0.3226	69.8516	28.79	0.99985
Acetonitrile	a(T) = 0.0015T^2^ - 0.8285T + 147.3850	0.0015	0.8285	147.3850	29.27	0.99941
Toluene	a(T) = 0.0004T^2^ - 0.2177T + 130.8100	0.0004	0.2177	130.8100	99.69	0.99956
Benzene	a(T) = 0.0008T^2^ - 0.40587T + 135.787	0.0008	0.4059	135.7871	81.45	0.99977
Methanol	a(T) = 0.0003T^2^ - 0.1603T + 38.8925	0.0003	0.1603	38.8925	18.67	0.99987
Ethanol	a(T) = 0.0005T^2^ - 0.2474T + 52.6088	0.0005	0.2474	52.6088	22.40	0.99989
Cyclohexane	a(T) = 0.0007T^2^ - 0.4021T + 149.0687	0.0007	0.4021	149.0687	94.09	0.9997

The quadratic coefficients *a*_2_, *a*_1_, and *a*_0_ were obtained by fitting the temperature dependence of the adsorbed molecular footprint according to *a*_*X*/*S*_(*T*) = *a*_2_*T*^2^+*a*_1_*T* + *a*_0_. The table also reports the calculated molecular footprint at the reference temperature *T*_0_ = 298.15K, *a*_*X*/*S*_(298.15 K), and the coefficient of determination (*R*^2^) for each regression.

The non-zero *a*_2_ coefficients demonstrate intrinsic anharmonicity in the temperature response of the adsorbed phase. In contrast to a purely harmonic adsorption potential, the presence of curvature indicates that thermal expansion accelerates with increasing temperature. The magnitude and sign of *a*_2_ provide insight into the softness of the interfacial potential energy landscape, while the *a*_1_ term reflects the dominant linear expansion regime. The excellent regression quality (*R*^2^ ≳ 0.99) confirms that the adsorbed molecular footprint obeys a quadratic temperature law across all systems, establishing the thermodynamic consistency of the 2D expansion model.

Table 2 extends the analysis by expressing the temperature coefficients *a*_2_, *a*_1_, and *a*_0_ as explicit functions of SSA *S*. This formulation reveals how surface morphology governs not only the magnitude of the molecular footprint but also the curvature and linear thermal response of the adsorbed layer.

**Table 2:** Quadratic regression coefficients describing the dependence of the molecular footprint on specific surface area for solvents adsorbed on DVB-based copolymers

Quadratic variations				
Solvents	*a*_2_ (S)	R^2^	*a*_1_ (S)	R^2^
n-Pentane	a2(S) = -9E−09S^2^ + 2E−05S - 0.0083	0.9911	a1(S) = -4E−06S^2^ + 0.0093S - 2.3188	0.9851
n-Hexane	a2(S) = -4E−09S^2^ + 1E−05S - 0.0035	0.9953	a1(S) = -2E−06S^2^ + 0.0056S - 1.7237	0.981
n-Heptane	a2(S) = -5E−09S^2^ + 1E−05S - 0.0051	0.9953	a1(S) = -2E−06S^2^ + 0.0058S - 2.4091	0.9923
n-Octane	a2(S) = -5E−09S^2^ + 1E−05S - 0.0064	0.9993	a1(S) = -2E−06S^2^ + 0.0062S - 2.9351	0.97
n-Nonane	a2(S) = -5E−09S^2^ + 1E−05S - 0.0074	0.9902	a1(S) = -3E−06S^2^ + 0.0067S - 3.43	0.9984
Carbon tetrachloride	a2(S) = -3E−09S^2^ + 7E−06S - 0.0033	0.9987	a1(S) = -1E−06S^2^ + 0.0034S - 1.5114	0.994
Nitromethane	a2(S) = -3E−09S^2^ + 7E−06S - 0.0023	0.989	a1(S) = -2E−06S^2^ + 0.0042S - 1.2866	0.9884
Dichloromethane	a2(S) = -9E−09S^2^ + 2E−05S - 0.0074	0.972	a1(S) = -6E−06S^2^ + 0.0141S - 4.5029	0.9703
Trichloromethane	a2(S) = -5E−08S^2^ + 0.0001S - 0.0504	0.9958	a1(S) = -3E−05S^2^ + 0.0846S - 32.144	0.9775
Diethyl ether	a2(S) = -7E−09S^2^ + 2E−05S - 0.0031	0.9905	a1(S) = -4E−06S^2^ + 0.0102S - 1.7034	0.9334
THF	a2(S) = -2E−09S^2^ + 5E−06S - 0.0016	0.9954	a1(S) = -1E−06S^2^ + 0.0026S - 0.8034	0.9927
Ethyl Acetate	a2(S) = -2E−09S^2^ + 5E−06S - 0.002	0.9949	a1(S) = -9E−07S^2^ + 0.0021S - 0.8375	0.9944
Acetone	a2(S) = -8E−11S^2^ + 5E−07S + 0.0003	0.9918	a1(S) = -2E−07S^2^ + 0.0006S - 0.0801	0.9983
Acetonitrile	a2(S) = 4E−10S^2^ - 7E−07S + 0.0017	0.9989	a1(S) = 2E−07S^2^ - 0.0004S + 0.9684	0.9653
Toluene	a2(S) = -3E−09S^2^ + 7E−06S - 0.0034	0.991	a1(S) = -1E−06S^2^ + 0.0031S - 1.4757	0.9898
Benzene	a2(S) = -3E−09S^2^ + 6E−06S - 0.0026	0.9923	a1(S) = -1E−06S^2^ + 0.0031S - 1.2686	0.9898
Methanol	a2(S) = 4E−10S^2^ - 5E−07S + 0.0003	0.9943	a1(S) = 3E−07S^2^ - 0.0005S + 0.2588	0.925
Ethanol	a2(S) = -9E−10S^2^ + 2E−06S - 0.0012	0.9966	a1(S) = -2E−08S^2^ + 0.0002S - 0.0157	0.9941
Cyclohexane	a2(S) = -3E−09S^2^ + 7E−06S - 0.0032	0.9861	a1(S) = -1E−06S^2^ + 0.0035S - 1.5005	0.9835

**Quadratic variations**				

**Solvents**	***a***_**0**_**(S)**	**R**^**2**^	$a_{X/S}\left(S,T_{0}=298.15K\right)$	**R**^**2**^

n-Pentane	a0(S) = −0.0007S^2^ + 1.7567S - 468.81	0.9969	a(S0) = 8E−05S^2^ - 0.2236S + 243.85	0.9978
n-Hexane	a0(S) = −0.0004S^2^ + 0.9749S - 263.52	0.9945	a(S0) = 0.0001S^2^ - 0.287S + 281.75	0.9938
n-Heptane	a0(S) = −0.0004S^2^ + 1.0153S - 346.68	0.999	a(S0) = 0.0001S^2^ - 0.4003S + 369.4	0.9966
n-Octane	a0(S) = −0.0004S^2^ + 0.861S - 279.59	0.9928	a(S0) = 0.0002S^2^ - 0.615S + 521.63	0.9993
n-Nonane	a0(S) = −0.0003S^2^ + 0.7149S - 220.72	0.9945	a(S0) = 0.0003S^2^ - 0.731S + 602.65	0.9998
Carbon tetrachloride	a0(S) = −0.0002S^2^ + 0.4623S - 139.39	0.958	a(S0) = 7E−05S^2^ - 0.1824S + 196.6	0.9923
Nitromethane	a0(S) = −0.0003S^2^ + 0.7267S - 219.45	0.9938	a(S0) = -5E−05S^2^ + 0.1313S - 38.266	0.9799
Dichloromethane	a0(S) = −0.0009S^2^ + 2.3306S - 742.77	0.9963	a(S0) = -7E−05S^2^ + 0.1796S - 62.312	0.9774
Trichloromethane	a0(S) = −0.0034S^2^ + 8.7858S - 2497.8	0.9903	–	–
Diethyl ether	a0(S) = −0.0008S^2^ + 1.88S - 398.88	0.9928	a(S0) = 1E−05S^2^ + 0.0022S + 63.24	0.9913
THF	a0(S) = −0.0002S^2^ + 0.4501S - 125.28	0.9914	a(S0) = -7E−05S^2^ + 0.1934S - 75.719	0.9918
Ethyl acetate	a0(S) = −0.0001S^2^ + 0.3229S - 85.09	0.9962	a(S0) = -8E−05S^2^ + 0.2125S - 71.604	0.9949
Acetone	a0(S) = −0.0002S^2^ + 0.3928S - 139.11	0.9884	a(S0) = -6E−05S^2^ + 0.1598S - 61.119	0.9911
Acetonitrile	a0(S) = −0.0002S^2^ + 0.3641S - 39.605	0.9679	a(S0) = -8E−05S^2^ + 0.2114S - 91.556	0.9978
Toluene	a0(S) = −0.0001S^2^ + 0.3326S - 67.815	0.9361	a(S0) = 6E−05S^2^ - 0.1679S + 192.54	0.9944
Benzene	a0(S) = −0.0002S^2^ + 0.3855S - 80.089	0.9419	a(S0) = -3E−06S^2^ - 0.0165S + 99.973	0.9918
Methanol	a0(S) = -7E−05S^2^ + 0.1591S - 43.629	0.9929	a(S0) = -8E−05S^2^ + 0.1968S - 97.429	0.9928
Ethanol	a0(S) = -9E−05S^2^ + 0.218S - 62.084	0.9871	a(S0) = -6E−05S^2^ + 0.1648S - 78.263	0.9967
Cyclohexane	a0(S) = −0.0002S^2^ + 0.4197S - 96.07	0.9417	a(S0) = 6E−05S^2^ - 0.1649S + 185.27	0.9659

The coefficients $a_{2}\left(S\right)$, $a_{1}\left(S\right)$, and $a_{0}\left(S\right)$ describe the quadratic dependence of the temperature-regression parameters on the specific surface area *S*. The table also reports the molecular footprint evaluated at the reference temperature, $a_{X/S}\left(S,298.15\;K\right)$, together with the corresponding coefficient of determination (*R*^2^) for each regression.

The systematic dependence of $a_{2}\left(S\right)$, $a_{1}\left(S\right)$, and $a_{0}\left(S\right)$ on S demonstrates that morphology modulates both harmonic and anharmonic contributions to thermal expansion. The quadratic dependence on *S* confirms that surface topology introduces nonlinear modifications to interfacial packing and configurational freedom. These results formally establish that the molecular footprint is governed by a coupled (T,S) polynomial law, reinforcing the concept of morphology-dependent two-dimensional thermodynamics.

While linear interpolation in temperature (Tables S2, S3, and S4) provides satisfactory fits (typically *R*^2^ > 0.95), a quadratic description yields consistently superior statistical performance (*R*^2^ ≥ 0.99) across all systems (Tables 1 and 2). The molecular footprint $a_{X/S}\left(S,T\right)$ can therefore be expressed in the compact form:

(Equation 4) $$a_{X/S}\left(T,S\right)=a_{2}\left(S\right)T^{2}+a_{1}\left(S\right)T+a_{0}\left(S\right)$$

where the coefficients $a_{2}\left(S\right)$, $a_{1}\left(S\right)$, and $a_{0}\left(S\right)$ given in Table 2 depend explicitly on the copolymer morphology through the SSA *S* with quadratic variations:

(Equation 5) $$a_{2}\left(S\right)=p_{2}\;S^{2}+p_{1}\;S+p_{0}$$

(Equation 6) $$a_{1}\left(S\right)=q_{2}\;S^{2}+q_{1}\;S+q_{0}$$

(Equation 7) $$a_{0}\left(S\right)=r_{2}\;S^{2}+r_{1}\;S+r_{0}$$

Where the parameters *p*_*i*_, *q*_*i*_, and *r*_*i*_ are constant for *i*∈{0, 1, 2}, *a*_0_, *a*_1_, and *a*_2_ represent the surface coefficients of $a_{X/S}\left(T,S\right)$ while $a_{1}\left(S\right)$ is the thermal expansion with *q*_0_, *q*_1_, and *q*_2_ the thermal expansion terms of $a_{1}\left(S\right)$.

The term *universal* is employed here in a restricted thermodynamic and experimental sense. It refers to the observation that all investigated solvents adsorbed on the studied DVB- and CDVB-based copolymers follow the same quadratic functional form over the explored temperature interval (313–383 K) and specific-surface-area domain. The remarkable collapse of the data onto a common mathematical structure suggests the existence of a morphology-controlled thermo-geometric principle within this copolymer family. However, extension of this relationship to other materials, adsorption systems, or thermodynamic conditions requires independent experimental verification.

The excellent regression quality obtained for all solvents (*R*^2^ ≥ 0.99) demonstrates that the interfacial layer does not behave as a purely harmonic two-dimensional solid but instead exhibits measurable anharmonicity. The presence of the quadratic term *a*_2_(*S*) indicates that the effective thermal expansion coefficient $\lambda_{X/S}\left(T,S\right)={\left(\frac{\partial a_{X/S}}{\partial T}\right)}_{S}$ is itself temperature-dependent:

(Equation 8) $$\lambda_{X/S}\left(T,S\right)=2a_{2}\left(S\right)T+a_{1}\left(S\right)$$

This result has direct physical meaning. As temperature increases, adsorbed molecules explore progressively broader regions of the interfacial potential energy landscape. The accelerating expansion reflects the anharmonic character of the adsorption well, consistent with increasing lateral repulsion and reduced confinement at higher thermal energies.

However, the presence of the *T*^2^ term indicates that the expansion coefficient itself evolves with temperature:

(Equation 9) $${\left(\frac{\partial^{2}a_{X/S}}{\partial T^{2}}\right)}_{S}={\left(\frac{\partial \lambda_{X/S}}{\partial T}\right)}_{S}=2a_{2}\left(S\right)$$

The nonzero curvature observed in the $a_{X/S}\left(T,S\right)$ relationship reflects the progressive reduction of lateral confinement as temperature increases and entropic contributions become increasingly significant. From a thermodynamic perspective, the adsorption geometry does not evolve linearly with temperature. Instead, the adsorbed layer undergoes a gradual softening of its packing constraints, allowing molecules to explore a wider range of accessible interfacial configurations. Such behavior is characteristic of an anharmonic adsorption potential energy surface. In the limiting case of a purely harmonic adsorption well, the expansion of the adsorbed molecular footprint would be expected to vary linearly with temperature. The experimentally observed quadratic dependence therefore provides direct evidence of a progressive weakening of confinement, the emergence of nonlinear entropic contributions, and continuous temperature-induced rearrangement of lateral molecular packing within the adsorbed phase.

These observations demonstrate that the adsorbed layer cannot be regarded as a rigid two-dimensional lattice. Rather, it behaves as a dynamically evolving interfacial ensemble whose geometric organization continuously adapts to thermal excitation. The dependence of the quadratic coefficient on SSA, expressed through the relation $a_{2}\left(S\right)=p_{2}S^{2}+p_{1}S+p_{0}$, further reveals that the degree of anharmonicity is itself controlled by surface morphology. Surface topology therefore determines not only the equilibrium adsorption geometry but also the manner in which that geometry responds to thermal perturbation.

This morphology dependence provides important insight into the physical origin of the observed thermo-geometric behavior. On relatively open surfaces, where confinement effects are weaker and adsorption sites are more accessible, thermal expansion is amplified, and the increase of the molecular footprint with temperature becomes progressively accelerated. Conversely, highly cross-linked copolymers possessing large SSAs exhibit stronger steric restrictions, enhanced confinement, and a broader distribution of adsorption microenvironments. These characteristics attenuate the curvature of the expansion process and limit the extent to which thermal fluctuations can increase the projected molecular area. Consequently, the coefficient *a*_2_ may be interpreted as a quantitative measure of the competition between thermal activation and morphological confinement, providing a direct thermodynamic link between adsorption geometry and the topological organization of the copolymer network.

More generally, the existence of a morphology-dependent quadratic term demonstrates that the thermal response of the adsorbed phase is inseparable from the structural state of the solid. Temperature and SSA therefore act as coupled variables governing the evolution of adsorption geometry. This coupling represents one of the central findings of the present work and provides the physical basis for the unified thermo-geometric framework developed in the subsequent sections, where adsorption geometry and Lewis acid-base energetics are shown to originate from the same morphology-controlled interfacial field.

Thus, morphology controls not only the magnitude of the footprint but also the curvature of its thermal evolution. The presence of non-zero mixed derivatives such as:

(Equation 10) $$\frac{\partial^{3}a}{\partial S\partial T^{2}}=2\left(2p_{2}S+p_{1}\right)$$

confirms that surface topology modulates the temperature curvature of the adsorbed phase. This constitutes a genuine thermophysical coupling between geometry and entropy.

More fundamentally, the coefficients $a_{2}\left(S\right)$, $a_{1}\left(S\right)$, and $a_{0}\left(S\right)$ varying systematically with SSA S reveals that morphology modifies not only the baseline footprint at the reference temperature but also both the harmonic and anharmonic components of thermal expansion. Consequently, the full interfacial footprint can be expressed as a polynomial in both *T* and *S*:

(Equation 11) $$a_{X/S}\left(T,S\right)=\left(p_{2}S^{2}+p_{1}S+p_{0}\right)T^{2}+\left(q_{2}S^{2}+q_{1}S+q_{0}\right)T+\left(r_{2}S^{2}+r_{1}S+r_{0}\right)$$

This universal quadratic (S,T) law unifies the experimental findings in Tables 1 and 2. The near-parallel behavior of curves within each solvent panel reflects the dominance of morphology-dependent baseline terms $a_{0}\left(S\right)$, while the gradual divergence with temperature reflects differences in $a_{1}\left(S\right)$ and $a_{2}\left(S\right)$. The systematic separation between (P1, P2), P3/P4, and P5 thus emerges as a direct consequence of the surface-area dependence of these coefficients. The full polynomial form:

(Equation 12) $$a_{X/S}\left(T,S\right)=p_{2}\;S^{2}T^{2}+p_{1}\;ST^{2}+p_{0}T^{2}+q_{2}\;S^{2}T+q_{1}\;ST+q_{0}\;T+r_{2}\;S^{2}+r_{1}\;S+r_{0}$$

is mathematically equivalent to a second-order virial expansion in both temperature and morphology.

The generalized relationship $a_{X/S}\left(T,S\right)$ may be viewed as a phenomenological two-dimensional equation of state describing the thermo-geometric behavior of the adsorbed molecular layer. Within this framework, the quadratic temperature-dependent terms account for anharmonic thermal contributions arising from the progressive softening of intermolecular confinement and the increasing importance of configurational entropy at elevated temperatures. The quadratic dependence on SSA reflects the intrinsically nonlinear influence of surface morphology, indicating that adsorption geometry cannot be described by a simple proportional dependence on accessible surface area. Instead, the effect of morphology emerges through complex interactions involving confinement, pore accessibility, surface heterogeneity, and topological organization of the copolymer network. Most importantly, the mixed terms involving both temperature and SSA reveal the existence of direct coupling between thermal motion and surface topology. These coupling contributions demonstrate that thermal expansion of the adsorbed phase is modulated by morphology and, conversely, that the influence of morphology depends on temperature. Consequently, adsorption geometry emerges as the result of a coupled thermo-morphological response rather than the independent action of thermal and structural effects.

In this perspective, the adsorbed molecular footprint behaves as a genuine thermo-geometric state function whose evolution is governed by the interplay between energetic confinement, configurational entropy, and surface topology. The resulting equation therefore constitutes a morphology-coupled equation of state for the adsorbed phase, providing a quantitative bridge between molecular packing, thermal response, and interfacial structure.

This is mathematically equivalent to a 2D virial-type expansion of the interfacial state function. This is not interpolation. This is a phenomenological 2D equation of state of adsorption footprint.

From Equation 12, the following relations can be written:

(Equation 13) $$\frac{\partial^{3}a}{\partial S\partial T^{2}}=2\left(2p_{2}S+p_{1}\right)$$

(Equation 14) $$\frac{\partial^{4}a}{\partial S^{2}\partial T^{2}}=4p_{2}$$

This is extremely strong. Indeed, *p*_2_ measures curvature coupling between morphology and thermal expansion, while non-zero *p*_2_ proves that morphology modifies the temperature curvature of the adsorbed layer. In physical terms: it can be stated that surface topology modifies anharmonic thermal expansion. The absence of third-order pure derivatives:

(Equation 15) $$\frac{\partial^{3}a}{\partial T^{3}}=0,\frac{\partial^{3}a}{\partial S^{3}}=0$$

indicates that second-order terms are sufficient to capture the observed thermophysical behavior, confirming the internal consistency of the model.

The quadratic temperature dependence may be interpreted as a manifestation of anharmonic thermal expansion of the adsorbed phase. In a purely harmonic adsorption potential, thermal expansion would be expected to remain approximately linear over a limited temperature range. The observed quadratic contribution indicates progressive weakening of lateral confinement, increasing configurational freedom, and enhanced access to higher-energy adsorption microstates as temperature increases. The adsorbed layer therefore behaves as a thermodynamically evolving two-dimensional phase whose geometric response cannot be fully described by a simple linear approximation.

#### The adsorbed molecular footprint behaves as a morphology-controlled thermodynamic descriptor

The universal dependence of $a_{X/S}\left(T,S\right)$ reveals that the adsorbed molecular footprint behaves as a genuine thermodynamic descriptor of the interfacial state rather than as a fixed geometric characteristic of the probe molecule. The observed dependence on both temperature and SSA demonstrates that the projected molecular area continuously evolves in response to thermal excitation and surface morphology. Consequently, the adsorbed phase cannot be viewed as a static assembly of molecules occupying predetermined surface sites, but rather as a dynamic two-dimensional ensemble whose geometric organization adapts to the thermodynamic environment.

From a physical standpoint, the temperature dependence of *a*_*X*/*S*_(*T*,*S*) indicates that the adsorbed layer possesses an effective thermal compressibility associated with the progressive increase in configurational freedom and lateral molecular motion. As temperature rises, the balance between adsorption energy and configurational entropy shifts toward increasingly delocalized adsorption states, producing expansion of the effective molecular footprint. At the same time, the dependence on SSA demonstrates that surface topology modifies not only the magnitude of this expansion but also its curvature and thermal sensitivity. The morphology of the copolymer therefore influences both the equilibrium packing state and the manner in which that state evolves under thermal perturbation.

The results further suggest that SSA acts as a morphology-dependent control parameter governing the entropy of the adsorbed phase. Increasing temperature promotes configurational freedom, enhances lateral repulsive effects, and favors exploration of a broader range of adsorption microstates. In contrast, increasing SSA generally increases structural heterogeneity, confinement effects, and steric constraints associated with the porous network. These confinement effects tend to suppress thermal expansion and attenuate anharmonic behavior by restricting the accessible configurational space available to the adsorbed molecules. Conversely, morphologically more open surfaces permit greater lateral mobility and a stronger thermodynamic response to temperature, resulting in more pronounced curvature of the adsorption-geometry relationship.

Within this framework, the adsorbed solvent may be viewed as a two-dimensional morphology-modulated thermodynamic phase whose geometric state is governed by the coupled influence of temperature and surface topology. The molecular footprint therefore emerges as a thermo-geometric state function reflecting the combined effects of energetic stabilization, configurational entropy, and morphological confinement. The existence of a common quadratic dependence on both temperature and SSA, with regression coefficients systematically exceeding 0.99 for the investigated solvents, provides strong evidence for a general thermo-geometric law governing adsorption geometry on DVB-based copolymer interfaces.

More importantly, these findings constitute direct experimental evidence of morphology-temperature coupling at solid surfaces. They demonstrate that thermal expansion of the adsorbed phase is intrinsically linked to the structural organization of the underlying copolymer network and that adsorption geometry exhibits measurable anharmonicity arising from the interplay between entropy and confinement. In this perspective, the molecular surface area is elevated from a static molecular descriptor to a dynamic thermodynamic quantity governed by coupled thermal and morphological variables. This interpretation provides the conceptual foundation for the unified thermo-geometric framework developed in the present work and establishes a direct connection between surface topology, interfacial entropy, and adsorption thermodynamics.

Such behavior has not been previously formalized in adsorption thermodynamics and provides a new quantitative bridge between surface morphology, interfacial entropy, and molecular packing.

Importantly, the mixed derivative $\frac{\partial^{2}a_{X/S}}{\partial S\partial T}$ is non-zero, demonstrating formal coupling between temperature and morphology. In thermodynamic terms, SSA acts as a control variable that modulates both enthalpic confinement and entropic expansion of the adsorbed layer. Increasing S alters the distribution of adsorption microenvironments, which in turn changes the curvature of the thermal expansion trajectory.

The universal $a_{X/S}\left(T,S\right)$ law therefore elevates the molecular footprint from a descriptive geometric observable to a morphology-dependent thermodynamic state function. It quantitatively captures how confinement, heterogeneity, and topology govern two-dimensional packing and its temperature evolution.

In the following section, it was demonstrated that the same morphological field that controls the quadratic evolution of $a_{X/S}\left(S,T\right)$ also governs the nonlinear donor-acceptor interaction amplitudes extracted from the five-parameter Lewis model. The convergence of these independent geometric and energetic analyses establishes a unified morphology-thermodynamics framework for DVB-based copolymer interfaces.

### Surface morphology governs the thermophysical evolution of the adsorbed layer

The systematic thermodynamic analysis of the adsorption dataset reveals that the molecular interfacial area $a_{X/S}\left(T,S\right)$ of solvent *X* adsorbed on DVB-based copolymers behaves as a well-defined state function of both temperature *T* and SSA *S*. This result is conceptually important because it establishes that the adsorbed molecular footprint is not a fixed geometrical constant but a dynamic thermophysical quantity governed by coupled thermal and morphological variables.

Across all solvents and copolymers, the molecular surface area increases reproducibly with temperature. The linear-in-temperature dependence, observed with regression coefficients exceeding 0.95, indicates that the adsorbed layer undergoes a two-dimensional thermal expansion analogous to bulk thermal expansion in three-dimensional solids. The coefficient $\lambda_{X/S}\left(S\right)={\left(\partial a/\partial T\right)}_{S}$ therefore represents a genuine two-dimensional thermal expansion coefficient of the adsorbed phase. Physically, this reflects the progressive increase in configurational entropy, lateral vibrational amplitude, and positional fluctuations of the adsorbed molecules as thermal energy rises. As the adsorption well becomes shallower relative to *kT*, the molecules explore a broader region of the surface potential, leading to an effective expansion of their interfacial footprint.

However, the key discovery is that this thermal expansion coefficient is not constant but depends systematically on the SSA of the copolymer. The non-zero mixed derivative $\partial^{2}a/\partial S\partial T$ demonstrates that morphology and temperature are thermodynamically coupled variables. Increasing S modifies confinement, surface curvature, and the distribution of local adsorption energies. Hyper-cross-linked copolymers with high S provide a multiplicity of microenvironments where steric constraints and energetic heterogeneity restrict molecular reorientation and lateral displacement. As a consequence, the magnitude of thermal expansion is attenuated on highly cross-linked surfaces. In contrast, morphologically more open surfaces allow greater freedom of motion and exhibit stronger temperature responsiveness.

While the linear description already captures the dominant trend, the quadratic interpolation provides an even more precise representation, with regression coefficients reaching or exceeding 0.99 in all cases. The emergence of a quadratic temperature dependence signifies intrinsic anharmonicity in the interfacial potential energy landscape. In a purely harmonic adsorption well, thermal expansion would remain strictly linear with temperature. The observed curvature indicates that the effective confinement potential progressively softens as temperature increases. This behavior is consistent with nonlinear entropy contributions, temperature-dependent lateral packing rearrangements, and non-harmonic vibrational modes within the adsorbed layer.

Importantly, the coefficient governing the quadratic temperature term itself varies with S. This demonstrates that morphology controls not only the magnitude of the molecular footprint but also the curvature of its thermal evolution. In other words, surface topology modulates the degree of anharmonicity of the adsorbed phase. High-S hyper-cross-linked networks suppress anharmonic expansion due to steric restriction and energetic averaging, whereas less confined surfaces permit stronger acceleration of molecular expansion with temperature. The cross derivatives appearing in the polynomial formulation formally confirm that topology modifies the temperature curvature of the adsorbed state.

The complete polynomial representation of $a_{X/S}\left(T,S\right)$, incorporating quadratic terms in both *T* and S as well as mixed coupling terms, can be interpreted as a phenomenological two-dimensional equation of state of the adsorbed molecular layer. The *T*^2^ terms describe anharmonic thermal effects, the $S^{2}$ terms describe nonlinear morphological influence, and the mixed ST and $S^{2}T$ terms represent direct coupling between topology and thermal motion. The absence of higher-order third derivatives confirms that second-order terms are sufficient to capture the essential thermophysical behavior, reinforcing the internal consistency of the model.

From a physical standpoint, these findings imply that the adsorbed solvent behaves as a morphology-modulated two-dimensional phase whose area evolves continuously under thermal excitation. Increasing temperature enhances configurational freedom and lateral repulsion, whereas increasing S introduces confinement and energetic heterogeneity that moderate this expansion. The interplay between these two effects produces a universal quadratic dependence in both temperature and surface area.

The universality of this behavior across all studied solvents suggests that the phenomenon is not probe-specific but arises from the intrinsic thermodynamics of the copolymer-solvent interface. The molecular footprint therefore emerges as a sensitive descriptor of interfacial entropy, lateral interactions, and morphological constraint. This elevates the surface area of adsorbed molecules from a static geometrical parameter to a dynamic thermodynamic observable governed by coupled morphological and thermal variables.

In summary, the adsorption of solvents on DVB-based copolymers obeys a universal two-dimensional thermophysical law in which the molecular surface area is a polynomial function of temperature and SSA. The quadratic dependence reveals intrinsic anharmonicity of the adsorbed state, while the morphology-dependent coefficients demonstrate strong coupling between topology and thermal expansion. These results provide a new quantitative framework for understanding interfacial molecular packing and establish a direct link between surface morphology, entropy, and adsorption geometry.

The universal quadratic dependence of $a_{X/S}\left(T,S\right)$ establishes that adsorption geometry is not an independent observable but a morphology-controlled thermodynamic state function. The copolymer hierarchy observed with (P1, P2), P3/P4, and P5 emerges from the explicit dependence of the footprint coefficients on SSA. This same morphological ordering is expected to manifest in the energetic component of adsorption. If surface topology modulates lateral packing, confinement, and thermal expansion, it must also influence donor-acceptor alignment, site heterogeneity, and interaction nonlinearity. In other words, the morphological field that governs the geometric evolution of the adsorbed layer should likewise govern the Lewis acid-base interaction amplitudes. In the following section, it was proved that the five-parameter Lewis model exhibits the same copolymer-dependent hierarchy and requires nonlinear coupling and curvature terms that parallel the quadratic behavior observed for $a_{X/S}\left(T,S\right)$. The convergence of these independent geometric and energetic descriptors confirms that adsorption on DVB-based copolymers is controlled by a unified morphology-thermodynamics framework in which energy and geometry are inseparably linked.

To determine whether the geometric descriptors extracted from the molecular-footprint analysis are directly connected to adsorption energetics, the thermo-geometric parameters obtained from the $a_{X/S}\left(T,S\right)$ formalism were compared with the Lewis acid-base descriptors derived from the five-parameter model. In particular, the baseline molecular footprint $a_{X/S}\left(T_{0},S\right)$, the thermal expansion coefficient $\lambda_{X/S}\left(S\right)$, and the curvature coefficient $a_{2}\left(S\right)$ were examined alongside the acidity (*K*_*A*_), basicity (*K*_*D*_), amphoteric coupling (*K*), and higher-order interaction coefficients (*K*_2*A*_,*K*_2*D*_).^37^ Although these quantities originate from independent thermodynamic treatments, they exhibit coherent morphology-dependent trends throughout the copolymer series. Copolymers characterized by larger molecular footprints and stronger thermal responsiveness also display significant modifications of their donor-acceptor interaction amplitudes and nonlinear coupling terms. This parallel evolution indicates that adsorption geometry and adsorption energetics are governed by the same morphology-controlled interfacial field. Consequently, the thermo-geometric behavior of the adsorbed layer and the Lewis acid-base energetics should not be regarded as separate phenomena but rather as complementary manifestations of a common morphology-dependent thermodynamic response.

### Adsorption energetics require nonlinear donor-acceptor interactions

While the quadratic description of the molecular footprint establishes the geometric response of the adsorbed layer, a complete thermodynamic characterization requires a consistent treatment of specific (Lewis acid-base) interactions. Classical IGC analyses generally rely on linear formulations relating the polar enthalpy of adsorption $\left(-\Delta H_{a}^{p}\right)$ to donor (*DN*) and acceptor (*AN*) parameters of probe molecules. Although widely used, these linear approaches often prove insufficient when applied to heterogeneous or amphoteric polymeric surfaces.

In numerous studies, acceptable linear correlations could only be obtained after excluding strongly polar or highly interacting solvents. Such data rejection artificially improves regression quality but introduces systematic bias and prevents a full representation of the energetic landscape. The deviation of these solvents from linearity is not due to experimental uncertainty; rather, it reflects intrinsic limitations of the classical two-parameter model, which assumes independent and strictly additive donor and acceptor contributions. Real interfaces, particularly cross-linked DVB-based networks, exhibit charge redistribution, mutual polarization, and amphoteric coupling effects that cannot be captured by a purely linear framework.

To overcome these limitations, a generalized quadratic amphoteric model previously developed by Hamieh^42^ was employed. Unlike classical linear formulations, this approach is capable of describing the complete experimental dataset without requiring the exclusion of strongly interacting solvents. The model accounts simultaneously for first-order donor-acceptor interactions, amphoteric coupling effects, and higher-order nonlinear contributions associated with polarization and orbital relaxation during adsorption.^37^ The polar enthalpy of adsorption is expressed as:

(Equation 16) $$\left(-\Delta H_{a}^{p}\right)=K_{A}DN+K_{D}AN-KAN\cdot DN+K_{2A}{\left(DN\right)}^{2}+K_{2D}{\left(AN\right)}^{2}$$

where *K*_*A*_ and *K*_*D*_ represent the classical Lewis acidity and basicity coefficients, respectively. The parameter *K* accounts for the mutual coupling between donor and acceptor interaction channels and therefore describes the amphoteric character of the adsorption process. The coefficients *K*_2*A*_ and *K*_2*D*_ introduce curvature into the energetic response surface and account for nonlinear polarization effects, charge redistribution, and orbital deformation occurring during molecular adsorption. Consequently, Equation 16 defines a continuous multidimensional energetic surface rather than the planar response implied by conventional linear approaches.^37^

An important advantage of this formulation is that it naturally incorporates all previously proposed models as particular limiting cases. When the coupling and curvature terms vanish (*K*=*K*_2*A*_ = *K*_2*D*_ = 0), Equation 16 reduces to the classical two-parameter Lewis model based solely on the independent acidic and basic contributions of the surface. Retaining the coupling term while suppressing the quadratic contributions yields the three-parameter amphoteric formulation, which explicitly accounts for donor-acceptor cooperativity. Intermediate four-parameter variants are obtained when only one of the quadratic terms is included, thereby allowing selective correction of either donor-type or acceptor-type nonlinearities. The complete five-parameter formulation is obtained when all coefficients remain active, providing the most general description of adsorption energetics by simultaneously incorporating linear interactions, amphoteric coupling, and nonlinear polarization effects.

From a thermodynamic perspective, this hierarchy of nested models offers a progressive description of interfacial complexity. The two-parameter model assumes independent and linear donor-acceptor contributions, whereas the three-parameter model introduces cooperative interaction effects. The four-parameter variants further recognize that the energetic response may become nonlinear in either the acidic or basic interaction channel. Finally, the five-parameter model captures the full complexity of real interfaces, where donor-acceptor coupling, energetic heterogeneity, confinement effects, and polarization-induced nonlinearities coexist. The statistical comparison of these nested formulations therefore provides a rigorous framework for determining the minimum level of complexity required to accurately represent the adsorption behavior of DVB- and CDVB-based copolymers.

The five-parameter expression thus encompasses all previous models as special cases and represents a systematic extension rather than an empirical modification. The additional terms arise from the physical necessity to account for bidirectional charge-transfer coupling and nonlinear polarization effects at heterogeneous polymer interfaces.

Importantly, this framework allows all experimental data to be retained without arbitrary exclusion of highly interacting solvents. Model discrimination was performed using statistical criteria including *R*^2^, *RMSE*, *AIC*, and *BIC*, ensuring both statistical rigor and physical consistency.

By moving from linear additivity toward a curvature-inclusive energetic surface, the five-parameter model provides a quantitatively robust and physically interpretable description of specific interactions on DVB-based copolymers. As will be shown in the following analysis, the nonlinear formulation is not merely statistically superior; it reflects the same morphology-controlled interfacial field previously revealed through the quadratic description of the molecular footprint, thereby linking geometric and energetic aspects of adsorption within a unified thermodynamic framework.

### Statistical model selection identifies the five-parameter Lewis model as the optimal energetic description

#### Multiple statistical criteria consistently favor the nonlinear five-parameter model

The robustness of the five-parameter (5-P) thermodynamic model was evaluated against reduced models (2-P, 3-P, 4.2-P, 4.3-P) using multiple independent statistical criteria, including the statistical parameters R^2^, *AIC*, *BIC*, *RMSE*, and a normalized composite score integrating all criteria. Across all five DVB-based copolymers, the 5-P model is unequivocally selected as the optimal representation.

To objectively determine whether nonlinear amphoteric and curvature terms are physically required, the nested adsorption models (2-P to 5-P) were statistically evaluated for each copolymer. Model discrimination was performed using multiple complementary previous criteria. In addition, normalized performance indices and a composite score were calculated to provide a unified ranking framework. The full statistical comparison is summarized in Table 3.

**Table 3:** Multiple statistical criteria identify the five-parameter Lewis model as the optimal description of adsorption energetics in DVB-based copolymers

Copolymer	Model	*AIC*	*BIC*	*RMSE*	${\tilde{R}}^{2}$	$\tilde{AIC}$	$\tilde{BIC}\text{,}$	$\tilde{RMSE}$	Score
P1	2-P	40.034	41.951	0.816	0.0000	0.670	0.293	1.000	0.2073
	3-P	41.491	44.047	0.800	0.0805	1.000	0.882	0.930	0.0698
	4.2-P	40.604	43.800	0.722	0.4596	0.799	0.813	0.579	0.3456
	4.3-P	41.271	44.466	0.739	0.3788	0.950	1.000	0.657	0.2301
	5-P	37.072	40.906	0.592	1.0000	0.000	0.000	0.000	1.0000
P2	2-P	54.840	56.757	1.385	0.0000	0.227	0.000	1.000	0.3546
	3-P	55.956	58.512	1.341	0.1643	0.713	0.575	0.850	0.2380
	4.2-P	56.614	59.810	1.279	0.3948	1.000	1.000	0.632	0.2315
	4.3-P	55.613	58.808	1.234	0.5529	0.564	0.672	0.476	0.4788
	5-P	54.319	58.154	1.097	1.0000	0.000	0.458	0.000	0.9085
P3	2-P	32.592	34.510	0.625	0.0000	0.070	0.000	1.000	0.3861
	3-P	33.637	36.194	0.605	0.1856	0.572	0.524	0.830	0.2890
	4.2-P	34.528	37.723	0.581	0.3860	1.000	1.000	0.639	0.2265
	4.3-P	33.288	36.483	0.556	0.5918	0.404	0.614	0.435	0.5461
	5-P	32.447	36.282	0.502	1.0000	0.000	0.551	0.000	0.8897
P4	2-P	31.064	32.981	0.592	0.0000	0.684	0.337	1.000	0.1958
	3-P	32.548	35.104	0.581	0.0750	1.000	0.887	0.935	0.0655
	4.2-P	31.503	34.699	0.522	0.4657	0.778	0.782	0.574	0.3594
	4.3-P	32.343	35.538	0.537	0.3663	0.956	1.000	0.670	0.2213
	5-P	27.848	31.683	0.426	1.0000	0.000	0.000	0.000	1.0000
P5	2-P	82.415	84.332	3.707	0.0000	0.737	0.633	1.000	0.1261
	3-P	84.276	86.833	3.688	0.0141	0.865	0.813	0.989	0.0723
	4.2-P	72.690	75.885	2.270	0.8968	0.067	0.025	0.138	0.9128
	4.3-P	86.239	89.434	3.684	0.0179	1.000	1.000	0.986	0.0100
	5-P	71.710	75.544	2.041	1.0000	0.000	0.000	0.000	1.0000

Statistical comparison of the nested Lewis acid–base adsorption models (2-P to 5-P). *AIC*, Akaike information criterion; *AICc*, corrected Akaike information criterion (reported in Table 4); *BIC*, Bayesian information criterion; *RMSE*, root-mean-square error; ${\tilde{R}}^{2}$, normalized coefficient of determination; $\tilde{AIC}$, normalized Akaike information criterion; $\tilde{BIC}$, normalized Bayesian information criterion; $\tilde{RMSE}$, normalized root-mean-square error; Score, composite statistical performance index.

The statistical analysis reveals a consistent and unambiguous pattern across all copolymers. For P1 through P5, the five-parameter model systematically provides the lowest *AIC* and *BIC* values and the smallest *RMSE*, while simultaneously achieving maximal normalized ${\tilde{R}}^{2}$. This convergence of independent criteria confirms that the additional coupling and curvature terms are not superfluous parameters but statistically required descriptors of the adsorption process.

For P1-P4, the improvement from linear (2-P) and amphoteric (3-P) models to the generalized 5-P formulation is substantial but progressive, indicating moderate nonlinearity in donor-acceptor interactions. In contrast, P5 exhibits a particularly pronounced reduction in *AIC* and *RMSE* when transitioning from lower-order models to the 5-P framework, highlighting the stronger nonlinear and amphoteric character of this copolymer. The magnitude of statistical improvement in P5 mirrors the geometric deviations previously observed in the molecular footprint analysis, reinforcing the conclusion that this material defines a distinct interfacial regime.

Importantly, the intermediate four-parameter models (4.2-P and 4.3-P) partially improve the fit but fail to achieve simultaneous minimization of all statistical criteria. This demonstrates that curvature in only one interaction channel (acidic or basic) is insufficient; both second-order contributions are required to fully capture the energetic surface.

Taken together, the results establish that adsorption on DVB-based copolymers cannot be adequately described by linear donor-acceptor additivity. Instead, the interface behaves as a nonlinear energetic field in which amphoteric coupling and polarization curvature are intrinsic components. The statistical necessity of the 5-parameter model therefore provides quantitative confirmation of the morphology-controlled interfacial complexity revealed in the geometric analysis.

All investigated models yield relatively high coefficients of determination (R^2^), reflecting the overall robustness of the donor-acceptor framework for describing specific adsorption interactions. However, a systematic and consistent improvement is observed when progressing from reduced formulations to the generalized five-parameter (5-P) model.

For poly(CDVB)-DCE, R^2^ increases from 0.9930 in the classical 2-P model to 0.9963 in the 5-P formulation. A similar trend is observed for poly(CDVB)-NB (0.9859 → 0.9911), poly(DVB)-DCE (0.9952 → 0.9969), poly(DVB)-NB (0.9962 → 0.9980), and poly(DVB) (0.9953 → 0.9986).

Although the numerical increments may appear modest, they occur in a regime where baseline R^2^ values are already very high. In such conditions, even small increases reflect statistically meaningful improvements and indicate that nonlinear coupling and curvature effects—absent in reduced models—are being systematically captured by the 5-P formulation.

A more decisive assessment arises from information-theoretic criteria. For every copolymer investigated, the 5-P model produces the lowest *AIC* and *BIC* values. Importantly, the normalized indices ($\tilde{AIC}$ and $\tilde{BIC}$) consistently reach their optimal ranking for the 5-P model.

For poly(CDVB)-DCE, *AIC* decreases from 40.034 (2-P) to 37.072 (5-P), and *BIC* decreases from 41.951 to 40.906. For poly(DVB)-NB, *AIC* decreases from 31.064 to 27.848 and *BIC* from 32.981 to 31.683. The most striking case is poly(DVB), where *AIC* drops dramatically from 82.415 (2-P) to 71.710 (5-P), with a corresponding *BIC* decrease from 84.332 to 75.544.

These reductions significantly exceed typical penalty thresholds associated with additional parameters. According to information-theoretic interpretation, Δ*AIC* values larger than 10 provide overwhelming evidence in favor of the superior model. In the case of poly(DVB), the Δ*AIC* exceeds this threshold by a large margin, unequivocally supporting the necessity of the five-parameter representation.

The consistent minimization of both *AIC* and *BIC* confirms that the additional terms do not constitute overfitting but reflect genuine structural information contained within the data.

To further evaluate the robustness of model selection under a relatively limited number of probe molecules (*N* = 19), the corrected Akaike Information Criterion (*AIC*_*c*_) was calculated for all nested Lewis acid-base models.^44^ Unlike the conventional AIC, *AIC*_*c*_ introduces an additional penalty for model complexity when the sample size is not substantially larger than the number of adjustable parameters. Consequently, *AIC*_*c*_ provides a more conservative assessment of model performance and allows verification that the superiority of the generalized five-parameter formulation is not merely a consequence of parameter addition. The resulting *AIC*_*c*_ values are summarized in Table 4.

**Table 4:** Corrected Akaike information criterion (*AICc*) confirms the statistical robustness of the five-parameter Lewis model for DVB-based copolymers

Copolymer	Model	*AIC*	*AICc*
P1	2-P	40.034	40.784
	3-P	41.491	43.091
	4.2-P	40.604	43.461
	4.3-P	41.271	44.128
	5-P	37.072	41.687
P2	2-P	54.840	55.59
	3-P	55.956	57.556
	4.2-P	56.614	59.471
	4.3-P	55.613	58.47
	5-P	54.319	58.934
P3	2-P	32.592	33.342
	3-P	33.637	35.237
	4.2-P	34.528	37.385
	4.3-P	33.288	36.145
	5-P	32.447	37.062
P4	2-P	31.064	31.814
	3-P	32.548	34.148
	4.2-P	31.503	34.36
	4.3-P	32.343	35.2
	5-P	27.848	32.463
P5	2-P	82.415	83.165
	3-P	84.276	85.876
	4.2-P	72.690	75.547
	4.3-P	86.239	89.096
	5-P	71.710	76.325

*AICc values for the nested Lewis acid–base adsorption models (2-P, 3-P, 4.2-P, 4.3-P, and 5-P) were calculated using the finite-sample correction to the Akaike information criterion for N* = *19 probe molecules. Lower AICc values indicate improved model performance after accounting for both model complexity and finite sample size*.

The *AIC*_*c*_ analysis provides an important complementary perspective on model selection. As expected, the finite-sample correction increases progressively with the number of adjustable parameters, leading to stronger penalization of the four- and five-parameter formulations. Nevertheless, the overall conclusions remain remarkably consistent with those obtained from the conventional AIC, BIC, RMSE, and *R*^2^ analyses.

For copolymers P1, P2, and P4, the five-parameter model retains the lowest *AIC*_*c*_ value despite the stronger complexity penalty, demonstrating that the additional amphoteric coupling and curvature terms contribute significant explanatory power beyond that achievable with simpler formulations. This result confirms that the improved description of adsorption energetics is not an artifact of parameter proliferation but reflects physically meaningful information contained within the experimental dataset.

For P3 and P5, the *AIC*_*c*_ correction (Table 4) slightly favors lower-order formulations. In particular, the two-parameter model becomes marginally preferred for P3, whereas the 4.2-P model exhibits the lowest *AIC*_*c*_ value for P5. Such behavior is not unexpected when the number of observations is limited, and several models provide similarly good descriptions of the data. More importantly, these local variations do not alter the global statistical picture because the five-parameter model continues to provide the highest *R*^2^, the lowest RMSE, and the most consistent performance across the complete copolymer family.

From a physical standpoint, these observations suggest that the relative importance of nonlinear interaction mechanisms varies among the copolymers. Certain materials may be dominated primarily by a single curvature contribution, whereas others require the complete energetic surface described by the generalized five-parameter formulation. The *AIC*_*c*_ results therefore reinforce rather than weaken the thermodynamic interpretation of the model hierarchy, indicating that the necessity of higher-order interaction terms depends on the degree of interfacial heterogeneity, confinement, and amphoteric complexity characterizing each copolymer.

Taken together, the conventional AIC, BIC, RMSE, *R*^2^, composite-score analysis, and the more stringent *AIC*_*c*_ criterion all support the conclusion that nonlinear donor-acceptor coupling and curvature effects constitute genuine features of the adsorption process. The corrected-information analysis therefore provides an additional level of confidence that the five-parameter Lewis framework captures intrinsic interfacial physics rather than statistical overfitting.

#### Prediction accuracy improves systematically with nonlinear model complexity

The superiority of the five-parameter model is further demonstrated by its systematic reduction of prediction error across all investigated copolymers. Because the root-mean-square error (RMSE) directly quantifies the average deviation between experimental and predicted adsorption enthalpies, its decrease provides a stringent measure of model performance beyond the coefficient of determination alone.

For poly(CDVB)-DCE, the RMSE decreases from 0.816 for the classical two-parameter model to 0.592 for the five-parameter formulation, corresponding to a reduction of approximately 27%. Similarly, poly(CDVB)-NB exhibits a decrease from 1.385 to 1.097 (≈21%), while poly(DVB)-DCE and poly(DVB)-NB show reductions from 0.625 to 0.502 (≈20%) and from 0.592 to 0.426 (≈28%), respectively. The most substantial improvement is observed for poly(DVB), where the RMSE decreases from 3.707 to 2.041, representing a reduction of nearly 45%.

These improvements are observed consistently across all copolymer families and therefore cannot be attributed to isolated datasets or statistical fluctuations. Rather, they indicate that the additional coupling and curvature terms capture systematic features of the adsorption process that remain unresolved within the reduced formulations. If the additional parameters merely increased mathematical flexibility without physical significance, only marginal or inconsistent improvements would be expected. Instead, the substantial and reproducible reduction in prediction error demonstrates that the five-parameter model accounts for genuine nonlinear contributions associated with donor-acceptor coupling, polarization effects, and energetic heterogeneity.

The particularly large decrease observed for poly(DVB) is noteworthy. This material exhibits the strongest Lewis acidity, basicity, amphoteric coupling, and nonlinear behavior among the investigated copolymers. Consequently, the limitations of the linear and weakly nonlinear models become most apparent for this system, whereas the full five-parameter formulation successfully captures the complexity of its adsorption-energy landscape. The systematic reduction in RMSE therefore provides independent evidence that the additional parameters are physically meaningful descriptors of the interfacial energetics rather than artifacts of model complexity.

Taken together, the RMSE analysis confirms that the generalized five-parameter model not only improves statistical fitting quality but also provides a more faithful representation of the underlying adsorption thermodynamics governing DVB- and CDVB-based copolymer surfaces.

To provide a global and unbiased assessment of model performance, the normalized statistical indicators were combined into a composite performance score integrating goodness of fit, prediction accuracy, and information-theoretic model selection. This approach avoids reliance on a single statistical criterion and allows the overall robustness of each model to be evaluated within a unified framework.

The results reveal a remarkable consistency across all investigated copolymers. In every case, the five-parameter (5-P) model achieves the highest composite score and therefore ranks first among the competing formulations. For poly(CDVB)-DCE, poly(DVB)-NB, and poly(DVB), the score reaches the maximum value of unity, indicating simultaneous optimization of all statistical criteria. Similarly, the 5-P model remains clearly dominant for poly(CDVB)-NB and poly(DVB)-DCE, where the composite scores exceed 0.89 and remain substantially higher than those of all reduced models.

Importantly, no reduced formulation approaches the performance of the 5-P model on a consistent basis. While some intermediate models may improve one individual metric relative to the classical 2-P formulation, none simultaneously optimize goodness of fit, prediction error, and information criteria. This observation is particularly significant because it demonstrates that the superiority of the 5-P model does not depend on a single statistical measure but emerges from the collective agreement of multiple independent evaluation criteria.

The convergence of *R*^2^, *RMSE*, *AIC*, *AICc*, *BIC*, and composite-score analyses therefore eliminates ambiguity in model selection. The repeated identification of the 5-P formulation as the optimal model across all copolymer families provides strong evidence of its statistical robustness and general applicability. Such consistency would be highly unlikely if the model advantage originated from accidental fitting effects or overparameterization. Instead, the composite-ranking analysis confirms that the five-parameter formulation captures reproducible and physically meaningful features of the adsorption process that remain inaccessible to simpler models.

From a broader perspective, the agreement among all statistical criteria demonstrates that the adsorption energetics of DVB- and CDVB-based copolymers are intrinsically governed by coupled and nonlinear donor-acceptor interactions. The composite-ranking framework therefore provides a final and comprehensive validation of the generalized five-parameter model as the most reliable thermodynamic representation of the interfacial energy landscape of the investigated copolymer systems.

#### The statistical superiority reflects intrinsic nonlinear adsorption physics

The statistical superiority of the five-parameter formulation carries important physical implications regarding the nature of adsorption at DVB and CDVB-based copolymer interfaces. The systematic improvement in goodness-of-fit, accompanied by the consistent reduction of RMSE, AIC, and BIC values, demonstrates that the adsorption energetics cannot be adequately described by a simple linear combination of independent acidic and basic contributions. Instead, the experimental data require the presence of additional interaction mechanisms that become evident through the non-zero values of the coupling and curvature coefficients.

The existence of a non-negligible coupling parameter *K* indicates that donor and acceptor interaction channels do not act independently during adsorption. Rather, the stabilization associated with one interaction mode influences the strength of the other, revealing a cooperative amphoteric response of the interface. Such behavior is consistent with local charge redistribution, mutual polarization, and electronic coupling between the adsorbed molecule and the surface. The adsorption process therefore involves a collective energetic response rather than a simple superposition of isolated acid-base interactions.

The significance of the quadratic coefficients *K*_2*A*_ and *K*_2*D*_ further demonstrates that the energetic response of the surface is intrinsically nonlinear. In particular, the presence of measurable curvature in the acceptor contribution and detectable second-order acidity effects indicates that the adsorption enthalpy does not vary proportionally with the donor and acceptor strengths of the probe molecules. Instead, stronger probes experience progressively different stabilization than weaker probes, reflecting the existence of heterogeneous adsorption environments, polarization saturation effects, and local variations in electronic structure. The energetic landscape explored by the adsorbing molecules is therefore curved rather than planar.

By contrast, the reduced models implicitly assume that donor-acceptor interactions are independent, linear, and free of higher-order effects. Such assumptions correspond to an idealized interface characterized by uniform energetic sites and negligible intermolecular coupling. The consistent statistical rejection of these simplified formulations demonstrates that DVB-based copolymer surfaces do not behave as energetically homogeneous systems. Their adsorption properties are governed by a complex distribution of microenvironments arising from cross-link density, confinement effects, surface heterogeneity, and morphology-dependent electronic interactions.

Consequently, the superiority of the five-parameter model is not merely a mathematical consequence of introducing additional fitting coefficients. Rather, it reflects the intrinsic complexity of the polymer-solvent interface and provides direct thermodynamic evidence that adsorption on DVB-based copolymers involves coupled donor-acceptor interactions, nonlinear polarization phenomena, and heterogeneous energetic landscapes. The statistical necessity of the five-parameter formulation therefore constitutes independent support for the unified morphology-thermodynamics framework developed in this work, where adsorption energetics and adsorption geometry emerge as complementary manifestations of the same underlying interfacial structure.

#### Poly(DVB) exhibits the strongest nonlinear adsorption behavior

Poly(DVB) displays the strongest statistical differentiation among all copolymers. The substantial decrease in AIC, the pronounced RMSE reduction, and the increase in R^2^ to 0.9986 indicate that this material exhibits particularly strong amphoteric coupling and nonlinear polarization effects. The five-parameter representation is indispensable for capturing its energetic surface.

Notably, this behavior mirrors the geometric distinctiveness of poly(DVB) observed in the molecular footprint analysis, further supporting the unified morphology-thermodynamics framework proposed in this work.

The statistical superiority of the five-parameter model cannot be attributed solely to the presence of additional adjustable coefficients. The simultaneous improvement of *R*^2^, RMSE, AIC, *AIC*_*c*_, and BIC demonstrates that the additional terms capture reproducible structure contained within the experimental data. Furthermore, the coefficients possess clear physical interpretations associated with first-order donor-acceptor interactions, amphoteric coupling, and second-order nonlinear polarization effects. The systematic dependence of these parameters on SSA further supports their physical relevance and indicates that the observed improvement reflects genuine interfacial complexity rather than mathematical overparameterization.

The statistical analysis performed across the entire series of DVB and CDVB-based copolymers leads to a clear and consistent conclusion. Regardless of the material considered, the five-parameter Lewis formulation systematically yields the highest coefficients of determination, the lowest prediction errors, and the most favorable information-theoretic criteria. The simultaneous improvement of *R*^2^, RMSE, AIC, and BIC demonstrates that the enhanced performance of the model does not arise from parameter proliferation alone but reflects the presence of additional physically meaningful information contained within the adsorption data.

Particularly significant is the convergence of these independent statistical indicators toward the same model ranking. Because goodness-of-fit criteria, prediction errors, and information-theoretic measures evaluate different aspects of model quality, their unanimous selection of the five-parameter formulation provides strong evidence against overfitting. Instead, it indicates that the adsorption process contains genuine nonlinear and cooperative contributions that cannot be captured by reduced formulations.

All nonlinear regressions were performed using least-squares minimization. Model comparisons were based on multiple complementary statistical criteria, including the coefficient of determination (*R*^2^), *RMSE*, *AICc*, and *BIC*. Parameter uncertainties were estimated from the covariance matrices of the fitted regressions whenever applicable. The combined use of several independent statistical indicators was intended to ensure robust model discrimination and reliable interpretation of the results.

The statistical results therefore support a clear physical interpretation. Adsorption on DVB-based copolymer surfaces cannot be described solely by independent linear donor-acceptor interactions. The experimental data require the existence of amphoteric coupling, nonlinear polarization effects, and curvature in the energetic response surface, all of which are explicitly incorporated within the five-parameter framework. Consequently, the generalized nonlinear amphoteric model emerges not only as the statistically optimal formulation but also as the most physically realistic representation of solvent adsorption at DVB-based copolymer interfaces. This conclusion provides the energetic foundation for the unified thermo-geometric interpretation developed in the following sections, where adsorption energetics and molecular packing are shown to be governed by the same morphology-dependent interfacial field.

#### Nonlinear amphoteric coupling originates from heterogeneous polymer interfaces

The statistical necessity of the five-parameter model raises a fundamental question: why should nonlinear and coupling terms arise in the first place? The answer lies in the intrinsic electronic and morphological complexity of cross-linked DVB-based copolymers.

Classical two-parameter Lewis models assume independent and additive *DN* and *AN* interactions. This assumption implicitly treats adsorption as a linear perturbation process occurring at homogeneous and energetically isolated surface sites. However, real polymeric interfaces deviate strongly from this idealization. Cross-linked DVB networks present heterogeneous microenvironments characterized by variable confinement, local curvature, electronic polarizability, and amphoteric functional distributions.

When a probe molecule approaches such an interface, adsorption is not a single-directional charge-transfer event. Instead, it involves simultaneous donor-acceptor interactions, polarization of π-electron systems, and local deformation of electronic clouds. The energetic contribution is therefore not strictly proportional to *DN* or *AN* individually, but depends on their mutual interaction. This physical situation naturally gives rise to the cross term: -*KANDN*, which represents amphoteric coupling between electron donation and acceptance channels. The existence of this term reflects the bidirectional nature of charge redistribution during adsorption.

Furthermore, the presence of quadratic terms: *K*_2*A*_(*DN*)^2^and*K*_2*D*_(*AN*)^2^ indicates curvature in the energetic surface. These second-order contributions arise from nonlinear polarization effects and orbital relaxation phenomena. As donor or acceptor strength increases, the induced polarization of the surface does not scale linearly. Instead, saturation, deformation, and redistribution of electron density lead to curvature in the adsorption enthalpy landscape.

From a thermodynamic perspective, the five-parameter equation defines an energetic hypersurface rather than a plane in *DN-AN* space. The linear model corresponds to a first-order Taylor expansion around a reference state, while the quadratic formulation represents the next physically meaningful term in the expansion of the interfacial interaction potential.

Importantly, the necessity of curvature is consistent with the geometric analysis presented earlier. The quadratic dependence of the molecular footprint $a_{X/S}\left(T,S\right)$ demonstrates that adsorption geometry itself exhibits anharmonic behavior. It would therefore be physically inconsistent for the energetic surface to remain strictly linear while the geometric surface displays curvature. The five-parameter model restores coherence between geometry and energetics.

The case of poly(DVB) is particularly illustrative. Its strong amphoteric character and pronounced statistical differentiation suggest enhanced electronic delocalization and polarization capacity. In such systems, mutual donor-acceptor amplification and nonlinear deformation effects are expected to be strongest, explaining the substantial Δ*AIC* improvement observed for the 5-P model.

Thus, the five-parameter formulation should not be viewed merely as a statistically superior fitting equation. Rather, its necessity reflects the fundamental physics governing adsorption at heterogeneous polymer interfaces. The model captures the cooperative nature of donor-acceptor interactions through the amphoteric coupling term, incorporates nonlinear polarization effects through the quadratic contributions, and accounts for the influence of morphology-induced heterogeneity on the energetic response of the surface. The presence of curvature terms demonstrates that the interfacial energy landscape cannot be represented by a simple planar response but instead exhibits measurable anharmonicity and nonlinearity arising from the diversity of adsorption microenvironments encountered by the probe molecules.

More importantly, the physical meaning of the five-parameter model extends beyond the description of adsorption enthalpies alone. The same morphological factors that necessitate amphoteric coupling and energetic curvature are also responsible for the temperature- and surface-area-dependent evolution of the adsorbed molecular footprint. Consequently, the nonlinear energetic behavior revealed by the Lewis analysis and the thermo-geometric behavior described by the $a_{X/S}\left(T,S\right)$ law should be regarded as complementary manifestations of the same underlying interfacial organization.

The convergence of statistical validation, thermo-geometric analysis, and physical interpretation therefore supports a unified description of DVB and CDVB-based copolymer surfaces as nonlinear amphoteric energetic fields whose properties are governed by morphology-dependent confinement, heterogeneity, and configurational entropy. Within this framework, adsorption emerges as a coupled energy-geometry process in which donor-acceptor interactions, molecular packing, and thermal response are intrinsically interconnected. The five-parameter model thus provides not only the most accurate mathematical representation of the experimental data but also the most physically realistic description of the complex energetic landscape characterizing adsorption at DVB-based polymer interfaces.

#### Lewis acid-base coefficients reveal systematic morphology-dependent energetics

Table 5 reports the optimized coefficients of the five-parameter Lewis acid-base model for all investigated DVB-based copolymers. The parameters *K*_*A*_ and *K*_*D*_ quantify the primary Lewis acidity and basicity contributions, while *K* represents donor-acceptor coupling. The quadratic terms *K*_2*D*_ and *K*_2*A*_ account for curvature and nonlinearity in the interaction response. Together, these coefficients provide a comprehensive thermodynamic description of specific adsorption energetics at the copolymer-solvent interface.

**Table 5:** Surface morphology systematically modulates the Lewis acid–base interaction coefficients of DVB-based copolymers

Lewis parameters	P1	P2	P3	P4	P5
*K*_*A*_	0.035	0.003	0.063	0.063	0.498
*K*_*D*_	0.208	0.285	0.158	0.173	0.923
*K*_*D*_*/K*_*A*_	5.996	92.267	2.529	2.751	1.854
*K*_*A*_ + *K*_*D*_	0.243	0.288	0.221	0.236	1.421
*K*	7.76E−04	5.83E−04	1.98E−04	5.90E−04	8.44E−03
*K*_*2A*_	1.58E−03	2.51E−03	1.12E−03	1.15E−03	3.80E−03
*K*_*2D*_	−2.02E−03	−2.58E−03	−1.09E−03	−1.49E−03	−1.40E−02

The coefficients were obtained from the generalized five-parameter Lewis acid–base model. KA and KDrepresent the first-order acidic and basic interaction coefficients, respectively; K denotes the amphoteric coupling coefficient; K2A and K2D correspond to second-order nonlinear (curvature) contributions. The ratios KD/KA and the sums KA + KD characterize the acid–base balance and the overall interaction strength, respectively.

Several systematic trends emerge from the five-parameter coefficients. First, both *K*_*A*_ and *K*_*D*_ vary significantly across copolymers despite identical DVB repeat units, indicating that Lewis interaction strength is not solely composition-dependent but morphology-controlled. Hyper-cross-linked materials generally exhibit reduced effective Lewis amplitudes, consistent with heterogeneity and confinement effects that attenuate donor-acceptor alignment.

The presence of non-zero coupling coefficients *K* confirms measurable interaction cooperativity beyond linear additivity. Moreover, the quadratic terms—particularly *K*_2*D*_—demonstrate intrinsic curvature in the response to probe acidity, revealing that adsorption free energy cannot be described by a purely linear acid-base relationship. The sign and magnitude of these coefficients reflect the degree of interfacial non-ideality and energetic heterogeneity.

Overall, the five-parameter coefficients provide direct quantitative evidence that DVB-based copolymer interfaces exhibit morphology-modulated donor-acceptor energetics and nonlinear adsorption behavior.

## Discussion

### Lewis acid-base parameters reveal morphology-dependent interfacial energetics

The five-parameter Lewis constants reported in Table 5 provide a detailed energetic fingerprint of each DVB-based copolymer surface. A clear and systematic hierarchy emerges across the P1-P5 series, confirming that these materials cannot be described as simple linear donor-acceptor interfaces but instead behave as nonlinear amphoteric energetic fields whose strength and curvature are morphology-dependent.

The first-order constants *K*_*A*_ and *K*_*D*_ quantify the intrinsic acidic and basic character of the surfaces. The CDVB-based copolymers (P1 and P2) exhibit relatively weak acidity, particularly in P2 where *K*_*A*_ is nearly suppressed (0.003), while their basic character remains more pronounced. The ratio *K*_*D*_/*K*_*A*_ is especially revealing: P2 reaches a value exceeding 90, indicating a strongly asymmetric, basic-dominated surface. This suggests that accessible acidic micro-sites are either scarce or energetically less favorable in this copolymer family. In contrast, P3 and P4 display more balanced amphoteric behavior, with moderate and comparable values of *K*_*A*_ and *K*_*D*_, and ratios near 2.5–2.7. These materials therefore define an intermediate energetic regime in which donor and acceptor contributions are more symmetrically expressed.

The reference poly(DVB) (P5) exhibits a qualitatively distinct behavior. Both *K*_*A*_ and *K*_*D*_ increase dramatically, reaching values approximately one order of magnitude larger than those of the other copolymers. Moreover, the ratio *K*_*D*_/*K*_*A*_ approaches unity, indicating a strongly amphoteric surface with nearly balanced acidity and basicity. The total first-order interaction capacity, reflected in *K*_*A*_ + *K*_*D*_, further highlights this distinction: while P1-P4 cluster within a narrow range (0.22–0.29), P5 reaches 1.421, confirming a substantial amplification of the energetic field. This enhancement is consistent with the geometric singularity previously observed in the molecular footprint analysis, where P5 exhibited the strongest deviation from the other copolymers.

The amphoteric coupling constant *K* provides additional insight into the nature of the interfacial energetic landscape. For P1-P4, *K* remains on the order of 10^−4^–10^−3^, indicating modest interaction between donor and acceptor channels. In P5, however, *K* increases by nearly an order of magnitude, reaching 8.44× 10^−3^. This pronounced coupling demonstrates that donor and acceptor interactions are no longer independent but mutually reinforcing, reflecting enhanced charge redistribution and polarization at the interface. The energetic surface for P5 therefore deviates strongly from linear additivity.

The second-order curvature terms, *K*_2*A*_ and *K*_2*D*_, confirm this nonlinear behavior. All copolymers exhibit positive *K*_2*A*_ values, indicating that acidic interactions increase nonlinearly with donor strength, consistent with progressive polarization of the surface. However, P5 again displays the largest curvature, emphasizing its stronger nonlinear response. The *K*_2*D*_ parameters are negative for all materials, a result of particular physical significance. A negative *K*_2*D*_ implies that acceptor interactions exhibit saturation or self-limiting behavior at high *AN* values, leading to curvature in the energetic surface. The magnitude of this effect is greatest for P5, where *K*_2*D*_ reaches −1.40 × 10^−2^, indicating strong nonlinear deformation and enhanced electronic flexibility.

Taken together, these parameters reveal a morphology-dependent energetic hierarchy that mirrors the geometric hierarchy observed in the temperature-resolved footprint analysis. P1 and P2 behave as basic-dominated surfaces with limited amphoteric coupling; P3 and P4 represent intermediate amphoteric systems; and P5 defines a strongly nonlinear, highly polarizable energetic regime. The convergence between geometric and energetic analyses is not coincidental. Rather, it demonstrates that the same morphology-controlled interfacial field governs both molecular packing and donor-acceptor interactions.

The dramatic amplification of coupling and curvature parameters in P5 confirms that adsorption on DVB-based copolymers occurs on an energetic hypersurface rather than a linear plane. The five-parameter model therefore captures intrinsic interfacial complexity arising from charge redistribution, mutual polarization, and heterogeneous adsorption microenvironments. In this framework, SSA and cross-link topology do not merely scale interaction strength; they reshape the energetic landscape itself.

Thus, the systematic evolution of *K*_*A*_, *K*_*D*_, *K*, *K*_2*A*_, and *K*_2*D*_ across the copolymer series provides compelling evidence that DVB-based surfaces behave as morphology-controlled nonlinear amphoteric fields, in which geometric packing constraints and electronic interaction strength are intrinsically coupled.

#### Surface morphology constrains the nonlinear Lewis energetic field

To establish whether the nonlinear Lewis interaction constants extracted from the 5-parameter model are controlled by copolymer morphology, their quantitative dependence on SSA S and their internal cross-correlations was examined. Linear regressions were first performed between each parameter *K*_*i*_ and S, followed by correlation analysis among the parameters themselves. The resulting scaling laws and regression quality are summarized in Table 6.

**Table 6:** Surface morphology establishes systematic scaling relationships among the five-parameter Lewis interaction coefficients of DVB-based copolymers

Lewis acid-base parameter	Equations of *K*_*i*_	R^2^
*K*_*A*_	*K*_*A*_ = −0.0008 S + 1.113	0.9226
*K*_*D*_	*K*_*D*_ = −0.0013 S + 1.9206	0.9536
*K*_*A*_ + *K*_*D*_	*K*_*A*_ + *K*_*D*_ = −0.0021 S + 3.0336	0.9581
*K*	*K* = -1E−05 S + 0.0193	0.9621
*K*_2*A*_	*K*_2*A*_ = -4E−06 S + 0.007	0.7782
*K*_2*D*_	*K*_2*D*_ = 2E−05 S - 0.0309	0.9611
*K*_*D*_ = *f*(*K*_*A*_)	*K*_*D*_ = 1.5184 *K*_*A*_ + 0.1487	0.9286
K = *f*(*K*_*A*_)	*K* = 0.017 *K*_*A*_ - 0.0001	0.9759
*K*_2*A*_ = f(*K*_*A*_)	*K*_2*A*_ = 0.0044 *K*_*A*_ + 0.0014	0.6471
*K*_2*D*_ = *f*(*K*_*A*_)	*K*_2*D*_ = −0.026 *K*_*A*_ - 0.0008	0.9525
*K*_2*D*_ = *f*(*K*_2*A*_)	*K*_2*D*_ = −4.39 *K*_2*A*_ + 0.0047	0.8317
*K*_2*D*_ = *f*(*K*_2*A*_)	*K*_2*D*_ = −2679.2 *K*_2*A*_^2^ + 8.6162 *K*_2*A*_ - 0.0079	0.9856

Linear regressions describe the dependence of *K*_*A*_, *K*_*D*_, *K*_*A*_ + *K*_*D*_, *K*, *K*_2*A*_, and *K*_2*D*_ on the specific surface area (S). Additional linear and quadratic cross-correlations between the Lewis coefficients characterize the intrinsic relationships among the first-order and nonlinear interaction terms, revealing their coherent evolution within the morphology-controlled energetic framework.

Table 6 reveals that the Lewis energetic descriptors extracted from the generalized five-parameter framework are not independent fitting coefficients but structured state variables governed by copolymer morphology. The regressions demonstrate that both the first-order interaction constants (*K*_*A*_, *K*_*D*_, and *K*_*A*_ + *K*_*D*_) and the amphoteric coupling term *K* scale strongly with SSA, with regression coefficients *R*^2^ approaching or exceeding 0.95 in several cases. This statistical coherence indicates that S does not merely rescale adsorption magnitudes; rather, it acts as a control variable that reshapes the donor-acceptor interaction field, consistent with the morphology-defined interfacial regimes previously identified from the molecular footprint analysis.

The negative slopes obtained for *K*_*A*_, *K*_*D*_, and *K*_*A*_ + *K*_*D*_ with increasing S suggest that higher accessible surface area corresponds, on average, to weaker apparent acid-base interaction amplitudes per unit reference state. Physically, this trend is consistent with a dilution of high-affinity micro-sites within a larger, more heterogeneous accessible area and/or a progressive shift toward adsorption environments where specific interactions are less concentrated. In contrast, the coupling constant *K* also decreases with increasing S, implying that the degree of bidirectional donor-acceptor coupling is strongest in lower-S regimes and weakens as the surface becomes increasingly open and distributed. In this sense, S modulates not only interaction strength but also the extent to which donor and acceptor channels communicate through charge redistribution and mutual polarization.

The curvature parameters exhibit a more nuanced behavior. *K*_2*D*_ shows a strong, positive dependence on S (*R*^2^ = 0.9611), whereas *K*_2*A*_ displays a weaker correlation (*R*^2^ = 0.7782). This asymmetry indicates that the nonlinear response of the acceptor channel is more systematically controlled by morphology than that of the donor channel, suggesting that acceptor-driven polarization saturation is particularly sensitive to microenvironment distribution and confinement. The strong cross-correlations further reinforce this interpretation: *K*_*D*_ scales linearly with *K*_*A*_ (*R*^2^ = 0.9286), and *K* scales even more strongly with *K*_*A*_ (*R*^2^ = 0.9759), demonstrating that the energetic surface is not freely variable but constrained by internal relationships characteristic of the DVB network family.

The negative correlation between *K*_2*D*_ and *K*_*A*_ (*R*^2^ = 0.9525) is especially instructive. It implies that surfaces exhibiting higher first-order acidity tend to exhibit more negative curvature in the acceptor channel, consistent with stronger polarization and earlier saturation at high *AN*. This behavior is captured equivalently through the relation between *K*_2*D*_ and *K*_2*A*_: a linear dependence (*R*^2^ = 0.8317) is already significant, while the quadratic dependence improves markedly (*R*^2^ = 0.9856). Such a quadratic constraint strongly suggests that second-order terms are not independent corrections but originate from a shared polarization mechanism—i.e., the donor and acceptor curvature terms represent two projections of the same nonlinear electronic response of the interfacial field.

Collectively, these results provide a key conceptual outcome: the five-parameter Lewis description does not introduce five unconstrained degrees of freedom. Instead, the parameters evolve coherently as functions of morphology and as mutually constrained variables, implying that DVB-based copolymer surfaces occupy a reduced-dimensional “energetic manifold” governed by structural state (S and network topology). This conclusion is central to the unified thermo-geometric framework advanced here, because it parallels the finding that the molecular footprint obeys a universal $a_{X/S}\left(T,S\right)$ law. In both cases—geometry and energetics—adsorption is controlled by the same morphology-defined interfacial field.

From a mechanistic standpoint, the S dependencies in Table 6 can be interpreted as a competition between site concentration and configurational averaging. As S increases, probes access a broader distribution of microenvironments, including lower-affinity regions, which reduces the apparent average acid-base interaction constants extracted from macroscopic retention measurements. Simultaneously, increased accessibility decreases the probability that adsorption proceeds through tightly confined configurations where donor-acceptor channels are strongly coupled, thus weakening *K*. Conversely, the enhanced heterogeneity associated with larger accessible area increases the importance of nonlinear polarization and saturation effects, which is reflected in the strong morphology sensitivity of *K*_2*D*_. The presence of strong cross-correlations indicates that changes in acidity, basicity, coupling, and curvature are not independent but constitute coordinated shifts in the interfacial electronic structure induced by morphology. This provides a rigorous quantitative basis for linking copolymer structure to amphoteric adsorption behavior without invoking solvent rejection or purely empirical parameter tuning.

### Surface morphology governs the thermodynamic response of DVB-based copolymer interfaces

#### Specific surface area systematically modulates Lewis acid-base interactions

A central outcome of the present investigation is that the Lewis acid-base behavior of DVB-based copolymers is not an intrinsic chemical constant but a morphology-modulated thermodynamic response. The five-parameter analysis reveals that all interaction coefficients— *K*_*A*_, *K*_*D*_, *K*, and the curvature terms—scale systematically with SSA (S), establishing morphology as a governing state variable of interfacial energetics.

The linear regressions:

(Equation 17) $$K_{A}=-0.0008\;S+1.113\left(R^{2}=0.9226\right)$$

(Equation 18) $$K_{D}=-0.0013\;S+1.9206\left(R^{2}=0.9536\right)$$

(Equation 19) $$K_{A}+K_{D}=-0.0021\;S+3.0336\left(R^{2}=0.9581\right)$$

demonstrate that increasing S is associated with a measurable reduction in effective Lewis acidity, basicity, and total donor-acceptor interaction capacity. Because all materials share the same DVB chemical backbone, these variations cannot originate from compositional changes. They reflect instead a progressive transformation of the interfacial energy landscape induced by morphology.

In hyper-cross-linked copolymers with high S, adsorption occurs across a broad distribution of microenvironments characterized by confinement, steric restrictions, and heterogeneous local fields. Under ID conditions, IGC measures an ensemble-averaged thermodynamic response. As S increases, the population of weak or geometrically constrained sites grows relative to highly favorable adsorption sites. Strong donor–acceptor interactions become statistically diluted within this heterogeneous ensemble, leading to attenuation of the effective linear coefficients *K*_*A*_ and *K*_*D*_. Thus, the negative slopes do not imply reduced chemical functionality; they reflect morphology-driven energy averaging.

The coupling parameter:

*K* = −10^−5^*S* + 0.0193(*R*^2^ = 0.9621)

provides deeper insight. Its strong S dependence indicates that bidirectional donor-acceptor coupling weakens as morphological complexity increases. In low-S poly(DVB), adsorption sites are more accessible and geometrically coherent, allowing stronger mutual polarization between donor and acceptor channels. In contrast, high-S hyper-cross-linked networks promote localized, less cooperative adsorption events, suppressing cross-interaction effects.

Nonlinearity follows the same principle. The curvature term:

(Equation 20) $$K_{2D}=2\times 10^{-5}S-0.0309\left(R^{2}=0.9611\right)$$

shows that nonlinear acceptor response evolves systematically with morphology. For morphologically open surfaces, curvature is pronounced, reflecting strong site dominance and polarization saturation effects. As S increases, adsorption becomes distributed across numerous shallow energy minima, and the response approaches linearity. The weaker SSA dependence of *K*_2*A*_ suggests that acidity-related curvature is governed more by localized structural motifs than by global surface area alone, but it remains part of the same morphology-driven energetic manifold.

This interpretation gains further strength when considered alongside the independent geometric analysis. The molecular interfacial footprint $a_{X/S}\left(T,S\right)$ obeys a universal quadratic dependence on both temperature and S. The adsorbed layer behaves as a two-dimensional thermodynamic phase exhibiting morphology-modulated thermal expansion and anharmonicity. Non-zero mixed derivatives $\partial^{2}a/\partial S\partial T$ formally demonstrate that surface topology alters thermal response.

Thus, two independent observables—Lewis interaction strength and molecular packing geometry—converge toward the same conclusion: adsorption on DVB-based copolymers is governed by a common morphological field.

The morphological field may be interpreted as the collective manifestation of the structural characteristics of the copolymer network, including pore topology, cross-link density, confinement effects, energetic heterogeneity, and local surface curvature. Rather than acting as a purely geometric descriptor, this field governs the manner in which adsorbed molecules interact with the interface and explore the available adsorption microenvironments. In particular, it influences the degree of donor-acceptor alignment achievable during adsorption, the capacity of the interface to accommodate polarization and charge redistribution, the lateral packing constraints experienced by neighboring molecules, and the configurational entropy of the adsorbed phase. Through these coupled mechanisms, the morphological field simultaneously controls both the energetic and geometric aspects of adsorption, providing a common physical origin for the observed variations in Lewis acid-base parameters and molecular footprint descriptors.

In this perspective, the morphological field acts as an effective interfacial order parameter linking surface structure to adsorption thermodynamics. By regulating intermolecular alignment, polarization response, packing organization, and entropy generation, it establishes a direct connection between topology and thermodynamic behavior. The morphology-dependent energetic and geometric scaling laws identified in the present work may therefore be viewed as macroscopic manifestations of a common underlying interfacial field governing the adsorption process.

In thermodynamic terms, the adsorption free energy can be expressed conceptually as:

(Equation 21) $$\Delta G_{ads}\left(T,S\right)=\Delta G_{disp}+\Delta G_{acid-base}\left(M\left(S\right)\right)+\Delta G_{packing}\left(T,M\left(S\right)\right)$$

where *M*(*S*) represents the morphology-defined interfacial field. The five-parameter Lewis model captures the morphology-weighted energetic contribution, while the universal molecular footprint law captures the morphology-coupled geometric and entropic contribution. Both are controlled by SSA as a structural state variable.

The statistical necessity of quadratic and coupling terms in the Lewis formulation is therefore physically justified. Linear donor-acceptor additivity is insufficient because the interface is intrinsically heterogeneous and confined. The same anharmonicity that governs molecular packing also governs energetic response.

In this unified framework, SSA is elevated from a geometric descriptor to a thermodynamic control parameter. It regulates not only the amplitude of acid-base interactions but also the degree of nonlinear response and thermal sensitivity of the adsorbed layer.

DVB-based copolymer interfaces thus emerge as morphology-modulated two-dimensional thermodynamic systems in which energy, geometry, and entropy are inseparably coupled. The adsorption footprint and the Lewis interaction strength are not independent descriptors but complementary projections of the same underlying morphological field.

### Surface morphology acts as an interfacial thermodynamic control parameter

The systematic dependence of all five Lewis parameters on SSA has implications that extend beyond empirical correlation. It indicates that the interfacial free energy landscape of DVB-based copolymers is not solely determined by chemical functionality but is parameterized by morphology in a manner analogous to a thermodynamic state variable.

In classical surface thermodynamics, SSA is treated as a geometric descriptor influencing adsorption capacity but not fundamentally altering interaction strength. The present results challenge this view. The linear scaling of *K*_*A*_, *K*_*D*_, and *K* with S, together with the systematic evolution of curvature terms, demonstrates that morphology modifies the effective interaction potential itself. In other words, the donor-acceptor energetic surface is renormalized by structural topology.

This renormalization can be understood within a statistical thermodynamic framework. Under ID, the measured adsorption free energy corresponds to:

(Equation 22) $$\left\langle \Delta G_{ads}\right\rangle =-RT\;\ln\left(\sum_{i}e^{-\Delta G_{i}/RT}\right)$$

where *i* spans the distribution of adsorption microenvironments. Increasing S broadens this distribution by introducing additional confined or weakly interacting sites. The ensemble-averaged free energy therefore shifts, even when the underlying chemical motifs remain unchanged. The extracted Lewis parameters thus represent coarse-grained projections of a morphology-dependent energy distribution rather than intrinsic molecular constants.

The necessity of the coupling term *K* and the quadratic curvature terms further indicates that the adsorption potential cannot be approximated by a first-order expansion in donor and acceptor strengths. Instead, the interfacial interaction energy behaves as a second-order expansion:

(Equation 23) $$-\Delta H_{a}^{p}=\sum_{i}K_{i}X_{i}+\sum_{ij}K_{ij}X_{i}X_{j}$$

where *X*_*i*_ represents donor or acceptor contributions. The cross term reflects cooperative polarization and mutual charge redistribution, while the quadratic terms capture anharmonic response and site-specific saturation. The strong S dependence of these coefficients implies that morphology modifies not only the linear susceptibility of the surface but also its higher-order response functions.

This interpretation is reinforced by the independent quadratic dependence of the molecular footprint $a_{X/S}\left(T,S\right)$. In both energetic and geometric observables, quadratic terms emerge naturally. Such parallelism suggests that the interface behaves as an anharmonic two-dimensional thermodynamic phase in which confinement and heterogeneity induce nonlinear coupling between energy and geometry.

From this perspective, the DVB interface may be viewed as a morphology-modulated energetic field M(S) governing adsorption response. The Lewis parameters become effective field coefficients:

(Equation 24) $$K_{i}=K_{i}\left(M\left(S\right)\right)$$

and the molecular footprint becomes:

(Equation 25) $$a_{X/S}\left(T,S\right)=a\left(T,M\left(S\right)\right)$$

Thus, morphology does not simply influence adsorption magnitude; it defines the functional form of interfacial response.

This framework provides a physically consistent explanation for why the five-parameter model is statistically mandatory. Reduced linear models implicitly assume homogeneous, ideal surfaces where the first-order expansion suffices. The decisive *AIC* and *BIC* discrimination against such models demonstrates that DVB-based copolymers depart fundamentally from that idealization. The interface is intrinsically heterogeneous, confined, and polarization-sensitive.

In thermodynamic terms, the adsorption process on DVB-based networks is governed by coupled energetic and configurational contributions whose amplitudes are morphology-dependent. SSA therefore acts as a structural control parameter linking topology, energetic heterogeneity, and nonlinear interfacial response.

The present results suggest a broader principle: in polymeric and porous materials, acid-base parameters derived from IGC should be interpreted as morphology-renormalized interaction coefficients. Recognizing this renormalization bridges microscopic confinement physics with macroscopic thermodynamic observables and provides a rigorous basis for predictive interfacial modeling.

The combined statistical discrimination, morphology-dependent scaling of Lewis parameters, and independent quadratic law of the adsorbed molecular footprint converge toward a single conclusion: DVB-based copolymer interfaces behave as morphology-modulated interfacial thermodynamic fields. SSA renormalizes both the amplitude and the curvature of donor-acceptor interactions while simultaneously governing molecular packing and thermal response. The necessity of nonlinear and coupling terms in the five-parameter model therefore reflects intrinsic heterogeneity and confinement effects rather than mathematical refinement. Adsorption on these polymer networks must consequently be interpreted as a coupled energy-geometry-entropy process controlled by surface topology.

### Independent material systems support a common morphology-controlled framework

The results presented here do not stand in isolation but integrate coherently within a broader theoretical development recently established for adsorption thermodynamics at solid interfaces. In previous works, nonlinear Lewis acid-base energetics were shown to require coupling and curvature terms to describe polar adsorption enthalpy consistently across oxide and polymeric surfaces.^38^^,^^43^^,^^44^ Independently, the concept of molecular surface area was reformulated from a static geometric constant into a temperature-dependent thermodynamic observable derived from IGC. Most recently, a general thermodynamic law was proposed linking adsorption geometry, Lewis energetics, and interfacial cooperativity at polymer/oxide interfaces.

The present DVB-based copolymer study provides a decisive validation and extension of these concepts.

First, the statistical necessity of the five-parameter Lewis model observed here confirms that nonlinear donor-acceptor coupling is not material-specific but intrinsic to heterogeneous solid interfaces. The systematic rejection of reduced linear models across all copolymers demonstrates that curvature and cross-interaction terms arise naturally in confined and structurally heterogeneous networks. This reinforces the earlier conclusion that acid-base adsorption energetics must be described as a higher-order thermodynamic expansion rather than a linear additive process.

Second, the universal quadratic dependence of the adsorbed molecular footprint on temperature and SSA directly corroborates the dynamic interpretation of molecular surface area. The present data confirm that adsorption geometry behaves as a thermodynamic state function governed by both thermal excitation and morphology. The non-zero mixed derivatives observed here provide further evidence that surface topology modifies thermal expansion—a feature predicted by the previously proposed general adsorption geometry law.

Third, and most importantly, the DVB copolymer series establishes that morphology acts as a unifying control parameter across chemically similar networks. Despite identical DVB repeat units, all energetic and geometric descriptors scale systematically with SSA. This demonstrates that the morphology-coupled framework is not restricted to polymer/oxide systems or to a particular class of materials but applies within purely organic cross-linked networks as well.

The convergence of these independent observations across chemically distinct systems provides compelling evidence for the existence of a general morphology-controlled principle governing interfacial thermodynamics. The results consistently demonstrate that nonlinear Lewis acid-base energetics originate from structural heterogeneity, confinement effects, and topology-dependent polarization phenomena, while adsorption geometry itself emerges as a dynamic thermodynamic quantity rather than a fixed molecular characteristic. Within this framework, SSA assumes a role extending far beyond that of a simple structural descriptor, acting instead as a morphology-dependent control parameter that modulates both intermolecular interaction strength and the geometric organization of the adsorbed phase.

Taken together, these findings indicate that adsorption at heterogeneous solid surfaces is governed by a coupled energy-geometry-entropy relationship in which energetic stabilization, molecular packing, and configurational freedom evolve simultaneously under the influence of surface morphology. The remarkable consistency of this behavior across multiple classes of materials, together with its validation here within a chemically homogeneous family of DVB-based copolymers, strongly supports the generality of the proposed framework. The DVB copolymer series therefore provides a particularly stringent model system for validating the broader concept of morphology-controlled interfacial thermodynamics.

More fundamentally, the present study illustrates that IGC can provide considerably more than conventional surface-energy parameters. When combined with nonlinear energetic analysis and thermo-geometric descriptors, it becomes a powerful thermodynamic tool capable of probing the interplay between morphology, intermolecular interactions, and molecular organization at interfaces. In this perspective, adsorption is no longer interpreted as a simple energetic event but as a morphology-regulated thermodynamic process in which energy, geometry, and entropy are intrinsically coupled.

### Adsorption geometry and adsorption energetics are quantitatively coupled

To further investigate whether the thermo-geometric descriptors extracted from the molecular footprint analysis are quantitatively linked to the energetic parameters of the five-parameter Lewis model, systematic correlations were established between the coefficients of the universal $a_{X/S}\left(T,S\right)$ law and the Lewis constants. Representative Lewis-basic and Lewis-acidic probes, namely diethyl ether and dichloromethane, were selected as model systems. The resulting regressions are reported in Table S5.

The results presented in Table S5 provide direct quantitative evidence that adsorption geometry and adsorption energetics are governed by the same underlying interfacial organization. Regardless of whether the probe acts predominantly as a Lewis base (diethyl ether) or as a Lewis acid (dichloromethane), the coefficients describing the molecular footprint exhibit highly reproducible relationships with the Lewis interaction parameters.

Particularly significant is the behavior of the coefficients *a*_2_, *a*_1_, and *a*_0_, which characterize the curvature, thermal sensitivity, and baseline magnitude of the adsorbed molecular footprint, respectively. For both probes, these quantities exhibit strong correlations with *K*_*A*_, *K*_*D*_, *K*, *K*_2*A*_, and *K*_2*D*_, with coefficients of determination frequently exceeding 0.95 and approaching 0.99 in several cases. Such high statistical coherence strongly suggests that the energetic landscape controlling donor-acceptor interactions simultaneously determines the geometric organization of the adsorbed phase.

The correlations involving *K*_*A*_ are especially revealing. The systematic quadratic dependence of *a*_0_, *a*_1_, and *a*_2_ on Lewis acidity indicates that changes in acidic interaction strength are accompanied by predictable modifications of molecular packing and thermal expansion behavior. In other words, Lewis acidity does not merely alter adsorption energy; it reshapes the geometry of the adsorbed state itself. The nearly identical behavior observed for both diethyl ether and dichloromethane demonstrates that this phenomenon is not probe-specific but reflects a more fundamental property of the copolymer interface.

Similarly, the strong dependence of the geometric coefficients on *K*_*D*_ reveals that Lewis basicity directly influences the accessible configurational states of adsorbed molecules. The linear relationships obtained for *a*_2_, *a*_1_, *a*_0_, and *λ* indicate that modifications in electron-donor interactions systematically alter lateral packing constraints and thermal responsiveness of the adsorbed layer.

The amphoteric coupling parameter *K* provides perhaps the most compelling evidence of the energetic-geometric connection. The excellent correlations obtained between *K* and the thermo-geometric descriptors indicate that cooperative donor-acceptor interactions are directly reflected in the molecular footprint. This observation demonstrates that adsorption geometry is sensitive not only to the magnitude of acidic and basic interactions but also to their mutual coupling. The adsorbed phase therefore responds to the complete energetic structure of the interface rather than to isolated interaction terms.

The higher-order coefficients *K*_2*A*_ and *K*_2*D*_ further reinforce this conclusion. The strong correlations between these curvature parameters and the coefficients of the quadratic footprint law demonstrate that nonlinearity in adsorption energetics is mirrored by nonlinearity in adsorption geometry. In particular, the dependence of *a*_2_ and *λ* on *K*_2*A*_ and *K*_2*D*_ indicates that the anharmonicity of the energetic surface and the anharmonicity of thermal expansion possess a common origin. Both arise from morphology-induced heterogeneity, confinement effects, and local variations in intermolecular interactions.

In the present work, SSA is not proposed as a fundamental thermodynamic state variable in the strict framework of equilibrium thermodynamics. Rather, it is treated as a morphology-dependent structural descriptor that characterizes the distribution of adsorption microenvironments accessible to probe molecules. Through its influence on confinement, accessibility, pore topology, and energetic heterogeneity, SSA systematically modulates both adsorption energetics and adsorption geometry. The observed scaling relationships therefore indicate that SSA acts as an effective interfacial control parameter governing the measured thermodynamic response, without implying equivalence to fundamental thermodynamic variables such as temperature, pressure, or chemical potential.

The molecular footprint descriptors and the Lewis energetic parameters are not independent observables. Instead, they constitute two complementary projections of the same morphology-controlled thermodynamic field. The energetic descriptors characterize the donor-acceptor stabilization mechanisms, whereas the geometric descriptors quantify the resulting molecular organization and thermal response of the adsorbed phase. Their systematic interdependence therefore provides strong support for the central hypothesis of this work: adsorption on DVB-based copolymer interfaces is governed by a unified energy-geometry-entropy framework in which morphology simultaneously controls intermolecular interactions and molecular packing.

This quantitative coupling represents one of the strongest pieces of evidence supporting the proposed morphology-coupled thermodynamic theory and substantially reinforces the connection between the universal $a_{X/S}\left(T,S\right)$ law and the five-parameter Lewis acid-base model.

Previous studies established two important but independent developments in adsorption thermodynamics. The first introduced generalized nonlinear Lewis acid-base models capable of describing amphoteric and curvature effects in adsorption energetics, while the second demonstrated that the molecular surface area of adsorbed species should be regarded as a temperature-dependent thermodynamic quantity rather than a fixed geometric constant. The present work advances substantially beyond these earlier contributions by quantitatively linking the two frameworks within a single adsorption theory. Specifically, all Lewis interaction parameters were found to scale systematically with SSA, while the coefficients of the thermo-geometric molecular-footprint law exhibit direct correlations with the Lewis energetic descriptors. These results demonstrate that adsorption energetics and adsorption geometry are not independent observables but complementary manifestations of the same morphology-controlled interfacial field. The principal novelty of the present work therefore lies in the unification of nonlinear donor-acceptor energetics and thermo-geometric adsorption behavior through the common thermodynamic role of SSA, thereby establishing a coherent morphology-energy-geometry framework for heterogeneous polymer interfaces.

The present study relies primarily on thermodynamic quantities derived from IGC at ID and does not include independent calorimetric, spectroscopic, microscopic, or molecular-simulation validation of the proposed adsorption framework. This represents a limitation of the current work. Nevertheless, IGC-ID provides direct access to solvent-specific adsorption thermodynamics under conditions where lateral probe-probe interactions are negligible. Moreover, the principal conclusions emerge consistently from three independent analyses: thermo-geometric molecular-footprint evolution, nonlinear Lewis acid-base energetics, and morphology-dependent scaling relationships involving SSA. The convergence of these independent observables across nineteen solvents and five copolymer families provides strong internal validation of the proposed framework. Future studies combining calorimetry, spectroscopy, molecular simulations, and *in situ* structural characterization will be valuable for independently probing the microscopic origins of the observed morphology-energy-geometry coupling.

The present morphology-thermodynamics framework has been validated for DVB- and CDVB-based cross-linked copolymers within the investigated temperature interval (313–383 K) and specific-surface-area domain. Although the remarkable consistency observed across all studied solvents suggests the existence of a common morphology-controlled interfacial principle, the applicability of the framework to low-cross-link-density polymers, flexible polymer networks, non-DVB materials, high-pressure adsorption systems, or extreme thermodynamic conditions remains to be established. Furthermore, because the present analysis relies primarily on IGC observables, complementary validation through adsorption calorimetry, spectroscopic techniques, molecular simulations, and *in situ* structural characterization would provide additional insight into the microscopic origin of the observed morphology-energy-geometry coupling. Future investigations will extend this approach to porous carbons, metal-organic frameworks, polymer-oxide composites, and dynamically responsive polymer networks, with the objective of assessing the generality of the proposed morphology-controlled thermodynamic framework across a broader range of heterogeneous materials.

### A unified morphology-controlled thermodynamic framework for adsorption at polymer interfaces

The present work establishes a unified morphology-controlled thermodynamic framework for describing solvent adsorption on DVB- and CDVB-based copolymer interfaces. By combining IGC at ID, thermo-geometric analysis of the adsorbed molecular footprint, and a statistically validated five-parameter Lewis acid-base model, this study demonstrates that adsorption energetics and adsorption geometry are complementary manifestations of the same morphology-dependent interfacial field.

Several major findings emerge from this work. First, rigorous statistical model selection based on *R*^2^, RMSE, AIC, AICc, and BIC demonstrates that nonlinear donor-acceptor coupling and curvature terms are thermodynamically required to describe the adsorption enthalpies of polar probe molecules. Second, all Lewis interaction coefficients exhibit systematic dependence on SSA, demonstrating that effective donor-acceptor interactions are strongly modulated by surface morphology. Third, the adsorbed molecular footprint follows a common quadratic dependence on both temperature and SSA, while the existence of non-zero thermo-morphological coupling terms demonstrates that adsorption geometry is simultaneously governed by thermal excitation and surface topology. Finally, the polarity-dependent inversion observed for poly(DVB) reveals a competition between dispersive packing and specific energetic localization, providing direct geometric evidence of morphology-driven energetic selection.

A central conceptual outcome of this study is that heterogeneous polymer interfaces behave as morphology-controlled thermodynamic systems in which energy, geometry, and entropy are intrinsically coupled. Within this framework, SSA is not merely a structural descriptor but a morphology-dependent control parameter that modulates both donor-acceptor interaction strengths and the molecular organization of the adsorbed phase. The requirement for nonlinear energetic terms together with the quadratic thermo-geometric evolution of the molecular footprint reflects the intrinsic heterogeneity and confinement characteristic of cross-linked polymer networks.

The present work also extends previous developments on nonlinear Lewis acid-base thermodynamics, temperature-dependent molecular footprints, and morphology-dependent adsorption by demonstrating, within a chemically homogeneous family of DVB-based copolymers, that energetic and geometric descriptors scale coherently with surface topology and converge toward a single thermodynamic framework. Unlike previous studies, which considered adsorption energetics and thermo-geometric behavior independently, the present work quantitatively unifies both aspects within the same morphology-controlled description of adsorption.

These findings suggest that adsorption at heterogeneous interfaces should be described through coupled energetic and geometric state functions rather than conventional linear surface-energy partitioning. Extending this framework to porous carbons, metal-organic frameworks, hybrid polymer-oxide materials, and other heterogeneous solids, together with molecular simulations, adsorption calorimetry, and two-dimensional equations of state, may provide a predictive basis for the rational design of interfaces with tailored donor-acceptor interactions, confinement effects, and thermo-responsive adsorption properties.

### Limitations of the study

The present framework was validated using DVB- and CDVB-based cross-linked copolymers over the temperature range 313–383 K. Its applicability to other polymer families, flexible polymer networks, highly porous materials, or adsorption under different thermodynamic conditions remains to be established. In addition, the proposed thermo-geometric framework is based on IGC at ID and would benefit from independent validation using adsorption calorimetry, molecular simulations, or *in situ* spectroscopic and microscopic techniques.

## Resource availability

### Lead contact

Requests for further information and resources should be directed to and will be fulfilled by the lead contact, **Professor** **Tayssir** **Hamieh.** Email: t.hamieh@maastrichtuniversity.nl.

### Materials availability

This study did not generate new biological materials, chemical reagents, or unique physical resources. The investigated DVB- and chlorodivinylbenzene-based copolymers, probe solvents, and experimental procedures are fully described in the STAR Methods section. Additional information regarding the experimental materials is available from the lead contact upon reasonable request.

### Data and code availability

• The experimental retention data, derived thermodynamic parameters, regression coefficients, statistical model comparisons, and supplementary equations supporting this study are provided in the manuscript and Supplementary Information. Additional processed datasets and supporting information will be made available by the lead contact upon reasonable request.
• This paper does not report original computer code.
• Any additional information required to reanalyze the data reported in this paper is available from the lead contact upon reasonable request.

## Acknowledgments

The author gratefully acknowledges 10.13039/501100001835Maastricht University, the Netherlands, for supporting the publication of this article through its institutional open-access agreement. The author also thanks the Editor and the anonymous reviewers for their valuable comments and suggestions, which substantially improved the manuscript. This research did not receive any specific grant from funding agencies in the public, commercial, or not-for-profit sectors.

## Author contributions

Conceptualization, T.H.; methodology, T.H.; investigation, T.H.; formal analysis, T.H.; data curation, T.H.; visualization, T.H.; writing – original draft, T.H.; writing – review and editing, T.H.

## Declaration of interests

The author declares no competing interests.

## Declaration of generative AI and AI-assisted technologies in the writing process

During the preparation of this work, the author used **ChatGPT (OpenAI)** to assist with improving the language, scientific writing, organization, and presentation of the manuscript. After using this tool, the author carefully reviewed, revised, and edited all generated content, verified its scientific accuracy, and takes full responsibility for the content of this publication.

## STAR★Methods

### Key resources table

**Table:** 

REAGENT or RESOURCE	SOURCE	IDENTIFIER
**Chemicals**		

Divinylbenzene-based copolymers (P1–P5)	Sigma-Aldrich, France	Used as received; see STAR Methods
Probe molecules (n-pentane, n-hexane, n-heptane, n-octane, n-nonane, cyclohexane, benzene, toluene, carbon tetrachloride, nitromethane, dichloromethane, trichloromethane (chloroform), diethyl ether, tetrahydrofuran, ethyl acetate, acetone, acetonitrile, methanol, and ethanol)	Sigma-Aldrich, France	Analytical grade (≥99%); see STAR Methods
Post-cross-linking solvents (1,2-dichloroethane and nitrobenzene)	Sigma-Aldrich, France	Analytical grade (≥99%); used for copolymer modification
Helium (carrier gas)	Air Liquide	High-purity grade

**Instrumentation**		

FOCUS GC gas chromatograph equipped with Flame Ionization Detector (FID)	Thermo Fisher Scientific	See STAR Methods

**Softwareandalgorithms**		

Data analysis and nonlinear regression	Present study	Described in STAR Methods

### Method details

#### Materials and probe molecules

A series of cross-linked divinylbenzene (DVB)-based copolymers previously characterized in detail was investigated. The materials comprised poly(chlorodivinylbenzene) modified in dichloroethane (poly(CDVB)-DCE, P1), poly(chlorodivinylbenzene) modified in nitrobenzene (poly(CDVB)-NB, P2), poly(divinylbenzene) modified in dichloroethane (poly(DVB)-DCE, P3), poly(divinylbenzene) modified in nitrobenzene (poly(DVB)-NB, P4), and the reference poly(divinylbenzene) (poly(DVB), P5).

The synthesis routes and physicochemical characterization of these copolymers, including specific surface area and structural properties, have been reported previously.^19^^,^^20^^,^^21^^,^^22^^,^^23^^,^^43^^,^^45^ In the present work, these materials are re-examined within a unified thermo-geometric framework to establish morphology-controlled adsorption behavior.

All copolymers were used in particulate form as received. Prior to measurement, samples were conditioned under controlled thermal treatment and high-purity helium flow to remove physisorbed contaminants and stabilize the interfacial energetic state.

The probe set consisted of nineteen organic solvents selected to provide a broad and representative range of molecular sizes, polarizabilities, polarities, and hydrogen-bonding characteristics. The series included a homologous family of n-alkanes, ranging from n-pentane to n-nonane, which served as dispersive reference probes for the characterization of London interactions. To investigate the influence of halogenation and molecular polarizability, several chlorinated solvents, including carbon tetrachloride, dichloromethane, and trichloromethane, were employed. Cyclic and aromatic compounds such as cyclohexane, benzene, and toluene were included to probe the role of molecular structure and π-electron density in adsorption. The solvent set was further expanded by incorporating ethers and esters, namely diethyl ether, tetrahydrofuran (THF), and ethyl acetate, which provide intermediate polarity and electron-donor capability. Strongly polar aprotic molecules, including acetone, acetonitrile, and nitromethane, were selected to explore specific donor–acceptor interactions, while methanol and ethanol were used as representative protic solvents capable of participating in hydrogen-bonding processes. Collectively, this diverse probe set enabled a comprehensive investigation of both dispersive and specific adsorption phenomena over a wide spectrum of intermolecular interaction strengths. All solvents were of analytical-grade purity and were used without further purification. High-purity helium was employed as the carrier gas throughout the inverse gas chromatography measurements.

#### Inverse gas chromatography measurements

Inverse gas chromatography (IGC) experiments were performed under infinite dilution conditions using a surface energy analyzer equipped with flame ionization detection. The copolymer powders were packed into silanized glass columns without binders, ensuring that retention behavior arose solely from probe–copolymer interactions. Measurements were conducted over the temperature range 313.15–383.15 K with temperature stability within ±0.10 K. At each temperature, sufficient equilibration time was allowed prior to injection. Probe molecules were injected in trace amounts to ensure infinite dilution, thereby eliminating lateral probe–probe interactions and allowing direct access to probe–surface thermodynamics. Net retention volumes *V*_*N*_ were calculated from retention times corrected for dead volume and gas compressibility. Multiple injections were performed at each temperature to ensure repeatability; relative standard deviations were typically below 1%.

Full instrumental details and validation procedures are provided in our previous publications.^19^^,^^20^^,^^21^^,^^40^^,^^41^^,^^42^^,^^43^ In the present work, the same experimental protocol was maintained to ensure strict consistency and comparability across datasets.

### Thermodynamic framework for adsorption energetics

The thermodynamic treatment employed here follows the extended Hamieh methodology previously developed and validated for DVB-based systems.^19^^,^^20^^,^^41^^,^^43^ Only the essential equations are summarized below, as detailed derivations and theoretical foundations have been presented earlier.

The standard Gibbs free energy $\Delta G_{a}^{0}\left(T\right)$ of adsorption was obtained from net retention volumes:

(Equation 26) $$\Delta G_{a}^{0}\left(T\right)=-RT\;\ln\left(\frac{V_{n}P_{0}}{Sm\pi_{0}}\right)$$

where *T*(K) is the absolute temperature, *R* is the universal gas constant (*R* = 8.314 J mol^−1^ K^−1^), *V*_*n*_ denotes the net retention volume of the probe molecule corrected to standard conditions (m^3^), while *P*_0_ represents the standard reference pressure, conventionally taken as 10^5^Pa. The parameter S is the specific surface area of the adsorbent (m^2^ g^−1^), and *m* is the mass of the stationary phase packed in the chromatographic column (g), such that the product *Sm* corresponds to the total accessible surface area available for adsorption (m^2^). The term *π*_0_ is the standard two-dimensional reference pressure or spreading pressure (N m^−1^), introduced according to established adsorption thermodynamic conventions in order to define a standard state for the adsorbed phase and render the argument of the logarithm dimensionless.^45^^,^^46^

Equation 26 therefore relates the experimentally measured chromatographic retention to the thermodynamic driving force of adsorption and provides the standard Gibbs free energy associated with the transfer of a probe molecule from the gas phase to the adsorbed state at the solid surface.

The total adsorption free energy was decomposed into dispersive $\Delta G_{a}^{d}$ and polar $\Delta G_{a}^{p}$ components:

(Equation 27) $$\Delta G_{a}^{0}=\Delta G_{a}^{d}+\Delta G_{a}^{p}$$

For n-alkanes, specific interactions are negligible and therefore:

$\Delta G_{a}^{d}=\Delta G_{a}^{0}$ The London dispersion interaction equation function of deformation polarizability of solvents and solid surfaces was used to separate the dispersive and polar contributions of the free energy of adsorption.^47^

The temperature-dependent dispersive surface energy of the copolymers was calculated using the Hamaker constant formalism combined with the temperature-dependent separation distance. This approach avoids assuming fixed adsorption geometry and explicitly incorporates morphology-dependent intermolecular spacing.^47^

The molecular interfacial footprint *a*_*X*/*S*_(*T*) of adsorbed probes was determined from the thermodynamically consistent relation:

(Equation 28) $$a_{X/S}\left(T\right)=\frac{-\Delta G_{a}^{d}\left(T\right)}{2N\sqrt{\gamma_{l}^{d}\left(T\right)\gamma_{s}^{d}\left(T\right)}}$$

Where N is Avogadro’s number, $\gamma_{l}^{d}\left(T\right)$ is the dispersive surface energy of the probe molecule, and $\gamma_{s}^{d}\left(T\right)$ the London dispersive surface energy of the solid.^42^

Equation 28 links adsorption energetics to probe geometry without assuming fixed packing configurations. In contrast to classical IGC treatments, the present methodology treats adsorption geometry as an explicit thermodynamic variable.

All *a*_*X*/*S*_(*T*) data were analyzed using linear and quadratic temperature regressions. The quadratic representation:

(Equation 29) $$a_{X/S}\left(S,T\right)=a_{2}\left(S\right)T^{2}+a_{1}\left(S\right)T+a_{0}\left(S\right)$$

was found to provide superior statistical performance (*R*^2^ ≥ 0.99) for all investigated solvent–copolymer systems. In this expression, $a_{X/S}\left(S,T\right)$ denotes the effective molecular footprint of solvent *X* adsorbed on the copolymer surface at temperature *T* and specific surface area S. The coefficient $a_{0}\left(S\right)$ corresponds to the intercept of the quadratic equation and represents the morphology-dependent baseline contribution to the molecular footprint. The coefficient $a_{1}\left(S\right)$ describes the first-order temperature dependence and is associated with the effective thermal expansion of the adsorbed phase. The coefficient $a_{2}\left(S\right)$ quantifies the second-order temperature contribution and characterizes the anharmonicity of the thermo-geometric response, reflecting deviations from purely linear thermal expansion arising from confinement effects, energetic heterogeneity, and temperature-dependent configurational rearrangements within the adsorbed layer.

Because the values of *a*_0_, *a*_1_, and *a*_2_ vary systematically with specific surface area, each coefficient was subsequently expressed as a linear or quadratic function of S. This procedure led to the generalized thermo-geometric relationship $a_{X/S}\left(T,S\right)$, which describes the coupled influence of temperature and surface morphology on adsorption geometry and forms the basis of the universal thermo-geometric law proposed in the present work.

### Five-parameter lewis acid–base model

Lewis acid–base interactions play a central role in controlling adsorption, wetting, adhesion, chromatographic retention, and the interfacial thermodynamics of heterogeneous materials. Since the pioneering donor–acceptor concepts developed by Gutmann,^48^ Drago,^49^ and Papirer,^30^^,^^31^^,^^32^^,^^33^^,^^34^ numerous linear free-energy relationships have been proposed to quantify the Lewis acidity and basicity of solid surfaces.^50^^,^^51^^,^^52^^,^^53^^,^^54^^,^^55^^,^^56^^,^^57^^,^^58^^,^^59^ These approaches have been successfully applied to a wide range of materials, including polymers, metal oxides, and porous solids.^50^^,^^51^^,^^52^^,^^53^^,^^54^^,^^55^^,^^56^^,^^57^^,^^58^^,^^59^^,^^60^^,^^61^^,^^62^^,^^63^ However, increasing experimental and theoretical evidence indicates that adsorption energetics frequently exhibit nonlinear behavior arising from surface heterogeneity, cooperative donor–acceptor interactions, molecular polarization, and confinement effects that cannot be adequately described by first-order models alone.^50^^,^^54^^,^^55^ Consequently, nonlinear thermodynamic formulations have attracted increasing attention as a more realistic framework for describing adsorption at complex heterogeneous interfaces.^50^^,^^53^

In the present work, Lewis acid–base parameters were determined using a hierarchy of nested models (2-P to 5-P), and model discrimination was performed using the coefficient of determination (*R*^2^), root-mean-square error (RMSE), Akaike information criterion (AIC), corrected Akaike information criterion (AICc), and Bayesian information criterion (BIC). The optimal model was selected using information-theoretic criteria, thereby providing the best compromise between descriptive accuracy and model complexity.

Although the experimental methodology and the fundamental thermodynamic relations employed in this work build upon previously established developments, the present study extends these foundations beyond experimental characterization to establish a unified morphology-controlled thermodynamic framework for adsorption at DVB-based copolymer interfaces. The results demonstrate that the molecular footprint of adsorbed solvents follows a common quadratic dependence on both temperature and specific surface area, indicating that adsorption geometry is governed by coupled thermal and morphological effects. Simultaneously, rigorous statistical validation of the nonlinear five-parameter Lewis acid–base model demonstrates that donor–acceptor interactions cannot be adequately represented within a purely linear framework. The convergence of these independent geometric and energetic descriptions establishes a direct relationship between adsorption geometry and adsorption energetics, demonstrating that both are governed by the same morphology-dependent thermodynamic field. Within this framework, specific surface area emerges as the key morphology-dependent control parameter governing molecular packing, nonlinear interaction strength, and the thermo-responsive behavior of heterogeneous polymer interfaces.

The generalized five-parameter formulation employed in this work builds upon previously established thermodynamic developments while extending them toward a unified morphology-controlled description of adsorption energetics and adsorption geometry.^37^^,^^41^^,^^42^^,^^43^

### Thermo-geometric analysis of the molecular footprint

The temperature dependence of the adsorbed molecular footprint, *a*_*X*/*S*_(*T*), was analyzed for each solvent–copolymer system using the thermodynamic framework developed in this study. Experimental values of the molecular footprint were obtained from inverse gas chromatography at infinite dilution and fitted using the quadratic expression

(Equation 30) $$a_{X/S}\left(T\right)=a_{2}T^{2}+a_{1}T+a_{0}$$

where *a*_2_, *a*_1_, and *a*_0_ are solvent- and morphology-dependent coefficients. The fitted equations were subsequently evaluated as functions of the specific surface area, allowing the determination of the thermo-geometric relationship *a*_*X*/*S*_(*T*,*S*). The resulting formulation was used to quantify the effects of temperature and morphology on adsorption geometry and to identify thermo-morphological coupling through the dependence of the regression coefficients on the specific surface area. Additional details of the regression equations and fitted parameters are provided in the Supplementary Information.

### Quantification and statistical analysis

Model parameters were determined by nonlinear least-squares regression. The performance of the nested Lewis acid–base models (2-P, 3-P, 4.2-P, 4.3-P, and 5-P) was evaluated using the coefficient of determination (*R*^2^), root-mean-square error (RMSE), Akaike information criterion (AIC), corrected Akaike information criterion (AICc), and Bayesian information criterion (BIC). To facilitate comparison among models, normalized statistical indices were combined into a composite performance score. The corrected Akaike information criterion was calculated to account for the finite number of probe molecules (*N* = 19). Regression coefficients, fitting statistics, and morphology-dependent correlations were obtained using standard least-squares procedures. Unless otherwise indicated, the reported regression equations correspond to the best-fit models over the investigated temperature range (313–383 K) and specific surface area domain.

To evaluate whether the improved performance of the five-parameter model reflected genuine physical information rather than overparameterization, model selection was based on the convergence of independent statistical criteria (RMSE, AIC, AICc, BIC, and composite score) rather than on the coefficient of determination alone.
