# Supplementary material for: Morphology controls nonlinear donor acceptor thermodynamics in divinylbenzene-based copolymers
Source: iScience. 2026 Aug 13;29(9):117141. doi: 10.1016/j.isci.2026.117141 (PMC13495352; doi:10.1016/j.isci.2026.117141)
Supplement: Supplementary file 1 [file mmc1.pdf]

iScience, Volume *29*

Supplemental information

Morphology controls nonlinear donor acceptor thermodynamics in divinylbenzene-based copolymers

Tayssir Hamieh

Supplemental Information

Morphology Controls Nonlinear Donor Acceptor Thermodynamics in Divinylbenzene Based Copolymers

Table S1.a. Variations of the surface area (Å^2) of solvents adsorbed on the different copolymers as a function of temperature.

Poly(CDVB)-DCE								
Solvents	313.15	323.15	333.15	343.15	353.15	363.15	373.15	383.15
n-Pentane	86.62	90.06	94.04	98.70	104.22	110.90	119.16	129.71
n-Hexane	92.43	95.27	98.47	102.10	106.25	111.05	116.66	123.32
n-Heptane	99.47	102.09	105.00	108.24	111.90	116.05	120.79	126.28
n-Octane	107.03	109.55	112.32	115.40	118.83	122.68	127.04	132.02
n-Nonane	114.64	117.11	119.83	122.83	126.15	129.86	134.03	138.75
Carbon tetrachloride	73.45	75.05	76.79	78.72	80.89	83.29	86.02	89.10
Nitromethane	52.36	54.09	56.03	58.27	60.87	63.91	67.54	71.94
Dichloromethane	54.10	56.75	59.89	63.70	68.45	74.55	82.76	94.55
Trichloromethane	68.83	74.64	82.33	93.20	110.20	142.30		
Diethyl ether	83.09	86.77	91.05	96.14	102.33	109.99	119.83	132.98
THF	53.49	54.89	56.45	58.20	60.18	62.44	65.05	68.08
Ethyl Acetate	66.53	67.72	69.00	70.42	71.99	73.73	75.69	77.87
Acetone	49.54	50.55	51.65	52.88	54.26	55.79	57.54	59.52
Acetonitrile	38.73	39.95	41.32	42.90	44.74	46.91	49.50	52.65
Toluene	79.13	80.59	82.17	83.90	85.83	87.96	90.35	93.03
Benzene	74.13	75.76	77.54	79.51	81.73	84.20	87.01	90.19
Methanol	28.53	28.95	29.41	29.92	30.48	31.11	31.83	32.63
Ethanol	37.85	38.50	39.20	39.98	40.86	41.82	42.92	44.15
Cyclohexane	73.99	75.70	77.57	79.65	81.99	84.60	87.57	90.95
Poly(CDVB)-NB								
Solvents	313.15	323.15	333.15	343.15	353.15	363.15	373.15	383.15
n-Pentane	86.96	90.41	94.40	99.05	104.58	111.27	119.55	130.11
n-Hexane	92.76	95.60	98.79	102.41	106.54	111.32	116.92	123.56
n-Heptane	99.98	102.59	105.49	108.73	112.37	116.51	121.24	126.72
n-Octane	107.54	110.04	112.79	115.85	119.25	123.08	127.41	132.35
n-Nonane	115.30	117.76	120.46	123.43	126.74	130.42	134.57	139.26
Carbon tetrachloride	73.74	75.33	77.07	79.01	81.15	83.56	86.26	89.33
Nitromethane	52.48	54.20	56.15	58.40	60.99	64.05	67.67	72.08
Dichloromethane	54.22	56.87	60.02	63.84	68.59	74.70	82.91	94.72

Trichloromethane	69.05	74.87	82.57	93.48	110.49	142.69		
Diethyl ether	83.36	87.04	91.33	96.44	102.61	110.30	120.13	133.29
THF	53.62	55.03	56.58	58.34	60.32	62.59	65.19	68.22
Ethyl Acetate	66.74	67.92	69.20	70.63	72.19	73.93	75.87	78.05
Acetone	49.59	50.61	51.72	52.96	54.33	55.88	57.62	59.61
Acetonitrile	38.70	39.92	41.30	42.90	44.74	46.92	49.52	52.68
Toluene	79.46	80.91	82.48	84.22	86.12	88.25	90.62	93.28
Benzene	74.40	76.02	77.80	79.78	81.98	84.46	87.24	90.41
Methanol	28.43	28.86	29.33	29.85	30.43	31.07	31.80	32.62
Ethanol	37.83	38.48	39.20	39.99	40.86	41.84	42.94	44.18
Cyclohexane	74.29	75.99	77.86	79.94	82.26	84.88	87.82	91.19
Poly(DVB)-DCE								
Solvents	313.15	323.15	333.15	343.15	353.15	363.15	373.15	383.15
n-Pentane	91.02	94.49	98.51	103.21	108.78	115.54	123.91	134.60
n-Hexane	95.51	98.40	101.66	105.36	109.58	114.47	120.18	126.97
n-Heptane	101.63	104.36	107.39	110.77	114.58	118.90	123.84	129.55
n-Octane	108.22	110.90	113.85	117.13	120.77	124.87	129.49	134.77
n-Nonane	115.08	117.79	120.76	124.03	127.65	131.69	136.22	141.35
Carbon tetrachloride	76.26	77.85	79.62	81.56	83.74	86.18	88.91	92.04
Nitromethane	56.74	58.38	60.25	62.39	64.89	67.82	71.32	75.59
Dichloromethane	58.73	61.35	64.49	68.30	73.06	79.20	87.47	99.43
Trichloromethane	72.95	78.91	86.86	98.09	115.69	149.04		
Diethyl ether	88.07	91.74	96.05	101.16	107.39	115.12	125.03	138.35
THF	57.65	58.95	60.40	62.03	63.89	66.01	68.45	71.31
Ethyl Acetate	70.62	71.69	72.88	74.18	75.64	77.25	79.04	81.09
Acetone	55.08	55.87	56.76	57.75	58.86	60.12	61.53	63.17
Acetonitrile	44.82	45.84	47.02	48.38	49.97	51.86	54.13	56.93
Toluene	82.26	83.68	85.25	86.96	88.86	90.98	93.32	95.99
Benzene	77.93	79.49	81.22	83.13	85.28	87.69	90.39	93.50
Methanol	35.01	35.11	35.23	35.36	35.53	35.72	35.94	36.22
Ethanol	43.67	44.05	44.49	44.97	45.51	46.13	46.83	47.64
Cyclohexane	76.91	78.60	80.49	82.57	84.91	87.54	90.50	93.91
Poly(DVB)-NB								
Solvents	313.15	323.15	333.15	343.15	353.15	363.15	373.15	383.15
n-Pentane	92.52	96.15	100.35	105.25	111.08	118.12	126.84	137.98
n-Hexane	97.65	100.67	104.07	107.93	112.34	117.43	123.39	130.46
n-Heptane	104.39	107.22	110.36	113.87	117.82	122.30	127.42	133.34
n-Octane	111.56	114.32	117.36	120.73	124.49	128.71	133.47	138.91
n-Nonane	118.98	121.75	124.78	128.13	131.83	135.96	140.60	145.85
Carbon tetrachloride	77.84	79.53	81.40	83.45	85.75	88.31	91.21	94.48
Nitromethane	57.02	58.78	60.78	63.07	65.74	68.86	72.60	77.13
Dichloromethane	58.99	61.74	65.03	69.02	74.00	80.38	89.01	101.42
Trichloromethane	73.91	80.05	88.24	99.79	117.87	152.04		
Diethyl ether	89.23	93.06	97.58	102.92	109.43	117.48	127.83	141.67

THF	58.05	59.46	61.05	62.82	64.83	67.12	69.77	72.85
Ethyl Acetate	71.50	72.69	74.01	75.44	77.04	78.80	80.79	83.01
Acetone	54.84	55.78	56.82	57.97	59.27	60.71	62.36	64.24
Acetonitrile	44.01	45.17	46.50	48.02	49.80	51.90	54.43	57.51
Toluene	83.94	85.46	87.14	88.96	91.00	93.24	95.76	98.58
Benzene	79.19	80.86	82.73	84.78	87.08	89.64	92.56	95.86
Methanol	33.70	33.95	34.23	34.54	34.89	35.29	35.75	36.27
Ethanol	42.92	43.45	44.03	44.68	45.40	46.21	47.13	48.16
Cyclohexane	78.48	80.27	82.27	84.47	86.94	89.70	92.85	96.42
Poly(DVB)								
Solvents	313.15	323.15	333.15	343.15	353.15	363.15	373.15	383.15
n-Pentane	98.27	100.89	103.94	107.55	111.89	117.19	123.84	132.43
n-Hexane	125.25	126.92	128.83	131.05	133.62	136.66	140.29	144.69
n-Heptane	148.25	149.22	150.35	151.65	153.17	154.95	157.06	159.58
n-Octane	181.26	181.59	182.01	182.53	183.16	183.95	184.92	186.14
n-Nonane	199.01	198.82	198.65	198.53	198.47	198.48	198.58	198.80
Carbon tetrachloride	96.06	96.56	97.14	97.79	98.54	99.41	100.43	101.63
Nitromethane	36.18	37.48	38.98	40.67	42.65	44.96	47.72	51.07
Dichloromethane	36.15	38.08	40.39	43.18	46.64	51.08	57.04	65.59
Trichloromethane	69.93	75.07	81.99	91.81	107.32	136.95		
Diethyl ether	82.63	85.48	88.86	92.89	97.82	103.98	111.95	122.71
THF	40.14	41.19	42.37	43.69	45.19	46.90	48.88	51.19
Ethyl Acetate	65.16	65.74	66.39	67.11	67.92	68.82	69.86	71.05
Acetone	29.62	30.40	31.26	32.21	33.27	34.46	35.80	37.34
Acetonitrile	30.35	31.49	32.80	34.29	36.02	38.05	40.48	43.44
Toluene	99.82	100.08	100.42	100.79	101.23	101.75	102.37	103.12
Benzene	82.16	82.94	83.83	84.81	85.93	87.21	88.68	90.39
Methanol	19.34	19.76	20.22	20.72	21.28	21.90	22.60	23.40
Ethanol	23.12	23.80	24.55	25.38	26.29	27.30	28.44	29.72
Cyclohexane	94.32	94.98	95.76	96.62	97.61	98.76	100.09	101.66

Table S1-b. Effect of the copolymer and temperature on the surface area (Å^2) of solvents.

n-Pentane					
T(K)	P1	P2	P3	P4	P5
313.15	86.62	86.96	91.02	92.52	98.27
323.15	90.06	90.41	94.49	96.15	100.89
333.15	94.04	94.40	98.51	100.35	103.94
343.15	98.70	99.05	103.21	105.25	107.55
353.15	104.22	104.58	108.78	111.08	111.89
363.15	110.90	111.27	115.54	118.12	117.19
373.15	119.16	119.55	123.91	126.84	123.84
383.15	129.71	130.11	134.60	137.98	132.43

n-Hexane					
T(K)	P1	P2	P3	P4	P5
313.15	92.43	92.76	95.51	97.65	125.25
323.15	95.27	95.60	98.40	100.67	126.92
333.15	98.47	98.79	101.66	104.07	128.83
343.15	102.10	102.41	105.36	107.93	131.05
353.15	106.25	106.54	109.58	112.34	133.62
363.15	111.05	111.32	114.47	117.43	136.66
373.15	116.66	116.92	120.18	123.39	140.29
383.15	123.32	123.56	126.97	130.46	144.69
n-Heptane					
T(K)	P1	P2	P3	P4	P5
313.15	99.47	99.98	101.63	104.39	148.25
323.15	102.09	102.59	104.36	107.22	149.22
333.15	105.00	105.49	107.39	110.36	150.35
343.15	108.24	108.73	110.77	113.87	151.65
353.15	111.90	112.37	114.58	117.82	153.17
363.15	116.05	116.51	118.90	122.30	154.95
373.15	120.79	121.24	123.84	127.42	157.06
383.15	126.28	126.72	129.55	133.34	159.58
n-Octane					
T(K)	P1	P2	P3	P4	P5
313.15	107.03	107.54	108.22	111.56	181.26
323.15	109.55	110.04	110.90	114.32	181.59
333.15	112.32	112.79	113.85	117.36	182.01
343.15	115.40	115.85	117.13	120.73	182.53
353.15	118.83	119.25	120.77	124.49	183.16
363.15	122.68	123.08	124.87	128.71	183.95
373.15	127.04	127.41	129.49	133.47	184.92
383.15	132.02	132.35	134.77	138.91	186.14
n-Nonane					
T(K)	P1	P2	P3	P4	P5
313.15	114.64	115.30	115.08	118.98	199.01
323.15	117.11	117.76	117.79	121.75	198.82
333.15	119.83	120.46	120.76	124.78	198.65
343.15	122.83	123.43	124.03	128.13	198.53
353.15	126.15	126.74	127.65	131.83	198.47
363.15	129.86	130.42	131.69	135.96	198.48
373.15	134.03	134.57	136.22	140.60	198.58
383.15	138.75	139.26	141.35	145.85	198.80
CCl_4					

T(K)	P1	P2	P3	P4	P5
313.15	73.45	73.74	76.26	77.84	96.06
323.15	75.05	75.33	77.85	79.53	96.56
333.15	76.79	77.07	79.62	81.40	97.14
343.15	78.72	79.01	81.56	83.45	97.79
353.15	80.89	81.15	83.74	85.75	98.54
363.15	83.29	83.56	86.18	88.31	99.41
373.15	86.02	86.26	88.91	91.21	100.43
383.15	89.10	89.33	92.04	94.48	101.63
Nitromethane					
T(K)	P1	P2	P3	P4	P5
313.15	52.36	52.48	56.74	57.02	36.18
323.15	54.09	54.20	58.38	58.78	37.48
333.15	56.03	56.15	60.25	60.78	38.98
343.15	58.27	58.40	62.39	63.07	40.67
353.15	60.87	60.99	64.89	65.74	42.65
363.15	63.91	64.05	67.82	68.86	44.96
373.15	67.54	67.67	71.32	72.60	47.72
383.15	71.94	72.08	75.59	77.13	51.07
Dichloromethane					
T(K)	P1	P2	P3	P4	P5
313.15	54.1	54.22	58.73	58.99	36.15
323.15	56.75	56.87	61.35	61.74	38.08
333.15	59.89	60.02	64.49	65.03	40.39
343.15	63.7	63.84	68.3	69.02	43.18
353.15	68.45	68.59	73.06	74	46.64
363.15	74.55	74.7	79.2	80.38	51.08
373.15	82.76	82.91	87.47	89.01	57.04
383.15	94.55	94.72	99.43	101.42	65.59
Trichloromethane					
T(K)	P1	P2	P3	P4	P5
313.15	68.83	69.05	72.95	73.91	69.93
323.15	74.64	74.87	78.91	80.05	75.07
333.15	82.33	82.57	86.86	88.24	81.99
343.15	93.20	93.48	98.09	99.79	91.81
353.15	110.20	110.49	115.69	117.87	107.32
363.15	142.30	142.69	149.04	152.04	136.95
Diethyl ether					
T(K)	P1	P2	P3	P4	P5
313.15	83.09	83.36	88.07	89.23	82.63

323.15	86.77	87.04	91.74	93.06	85.48
333.15	91.05	91.33	96.05	97.58	88.86
343.15	96.14	96.44	101.16	102.92	92.89
353.15	102.33	102.61	107.39	109.43	97.82
363.15	109.99	110.30	115.12	117.48	103.98
373.15	119.83	120.13	125.03	127.83	111.95
383.15	132.98	133.29	138.35	141.67	122.71
THF					
T(K)	P1	P2	P3	P4	P5
313.15	53.49	53.62	57.65	58.05	40.14
323.15	54.89	55.03	58.95	59.46	41.19
333.15	56.45	56.58	60.40	61.05	42.37
343.15	58.20	58.34	62.03	62.82	43.69
353.15	60.18	60.32	63.89	64.83	45.19
363.15	62.44	62.59	66.01	67.12	46.90
373.15	65.05	65.19	68.45	69.77	48.88
383.15	68.08	68.22	71.31	72.85	51.19
Ethyl Acetate					
T(K)	P1	P2	P3	P4	P5
313.15	66.53	66.74	70.62	71.50	65.16
323.15	67.72	67.92	71.69	72.69	65.74
333.15	69.00	69.20	72.88	74.01	66.39
343.15	70.42	70.63	74.18	75.44	67.11
353.15	71.99	72.19	75.64	77.04	67.92
363.15	73.73	73.93	77.25	78.80	68.82
373.15	75.69	75.87	79.04	80.79	69.86
383.15	77.87	78.05	81.09	83.01	71.05
Acetone					
T(K)	P1	P2	P3	P4	P5
313.15	49.54	49.59	55.08	54.84	29.62
323.15	50.55	50.61	55.87	55.78	30.40
333.15	51.65	51.72	56.76	56.82	31.26
343.15	52.88	52.96	57.75	57.97	32.21
353.15	54.26	54.33	58.86	59.27	33.27
363.15	55.79	55.88	60.12	60.71	34.46
373.15	57.54	57.62	61.53	62.36	35.80
383.15	59.52	59.61	63.17	64.24	37.34
Acetonitrile					
T(K)	P1	P2	P3	P4	P5
313.15	38.73	38.70	44.82	44.01	30.35
323.15	39.95	39.92	45.84	45.17	31.49

333.15	41.32	41.30	47.02	46.50	32.80
343.15	42.90	42.90	48.38	48.02	34.29
353.15	44.74	44.74	49.97	49.80	36.02
363.15	46.91	46.92	51.86	51.90	38.05
373.15	49.50	49.52	54.13	54.43	40.48
383.15	52.65	52.68	56.93	57.51	43.44
Toluene					
T(K)	P1	P2	P3	P4	P5
313.15	79.13	79.46	82.26	83.94	99.82
323.15	80.59	80.91	83.68	85.46	100.08
333.15	82.17	82.48	85.25	87.14	100.42
343.15	83.90	84.22	86.96	88.96	100.79
353.15	85.83	86.12	88.86	91.00	101.23
363.15	87.96	88.25	90.98	93.24	101.75
373.15	90.35	90.62	93.32	95.76	102.37
383.15	93.03	93.28	95.99	98.58	103.12
Benzene					
T(K)	P1	P2	P3	P4	P5
313.15	74.13	74.40	77.93	79.19	82.16
323.15	75.76	76.02	79.49	80.86	82.94
333.15	77.54	77.80	81.22	82.73	83.83
343.15	79.51	79.78	83.13	84.78	84.81
353.15	81.73	81.98	85.28	87.08	85.93
363.15	84.20	84.46	87.69	89.64	87.21
373.15	87.01	87.24	90.39	92.56	88.68
383.15	90.19	90.41	93.50	95.86	90.39
Methanol					
T(K)	P1	P2	P3	P4	P5
313.15	28.53	28.43	35.01	33.70	19.34
323.15	28.95	28.86	35.11	33.95	19.76
333.15	29.41	29.33	35.23	34.23	20.22
343.15	29.92	29.85	35.36	34.54	20.72
353.15	30.48	30.43	35.53	34.89	21.28
363.15	31.11	31.07	35.72	35.29	21.90
373.15	31.83	31.80	35.94	35.75	22.60
383.15	32.63	32.62	36.22	36.27	23.40
Ethanol					
T(K)	P1	P2	P3	P4	P5
313.15	37.85	37.83	43.67	42.92	23.12
323.15	38.50	38.48	44.05	43.45	23.80
333.15	39.20	39.20	44.49	44.03	24.55

343.15	39.98	39.99	44.97	44.68	25.38
353.15	40.86	40.86	45.51	45.40	26.29
363.15	41.82	41.84	46.13	46.21	27.30
373.15	42.92	42.94	46.83	47.13	28.44
383.15	44.15	44.18	47.64	48.16	29.72
Cyclohexane					
T(K)	P1	P2	P3	P4	P5
313.15	73.99	74.29	76.91	78.48	94.32
323.15	75.70	75.99	78.60	80.27	94.98
333.15	77.57	77.86	80.49	82.27	95.76
343.15	79.65	79.94	82.57	84.47	96.62
353.15	81.99	82.26	84.91	86.94	97.61
363.15	84.60	84.88	87.54	89.70	98.76
373.15	87.57	87.82	90.50	92.85	100.09
383.15	90.95	91.19	93.91	96.42	101.66

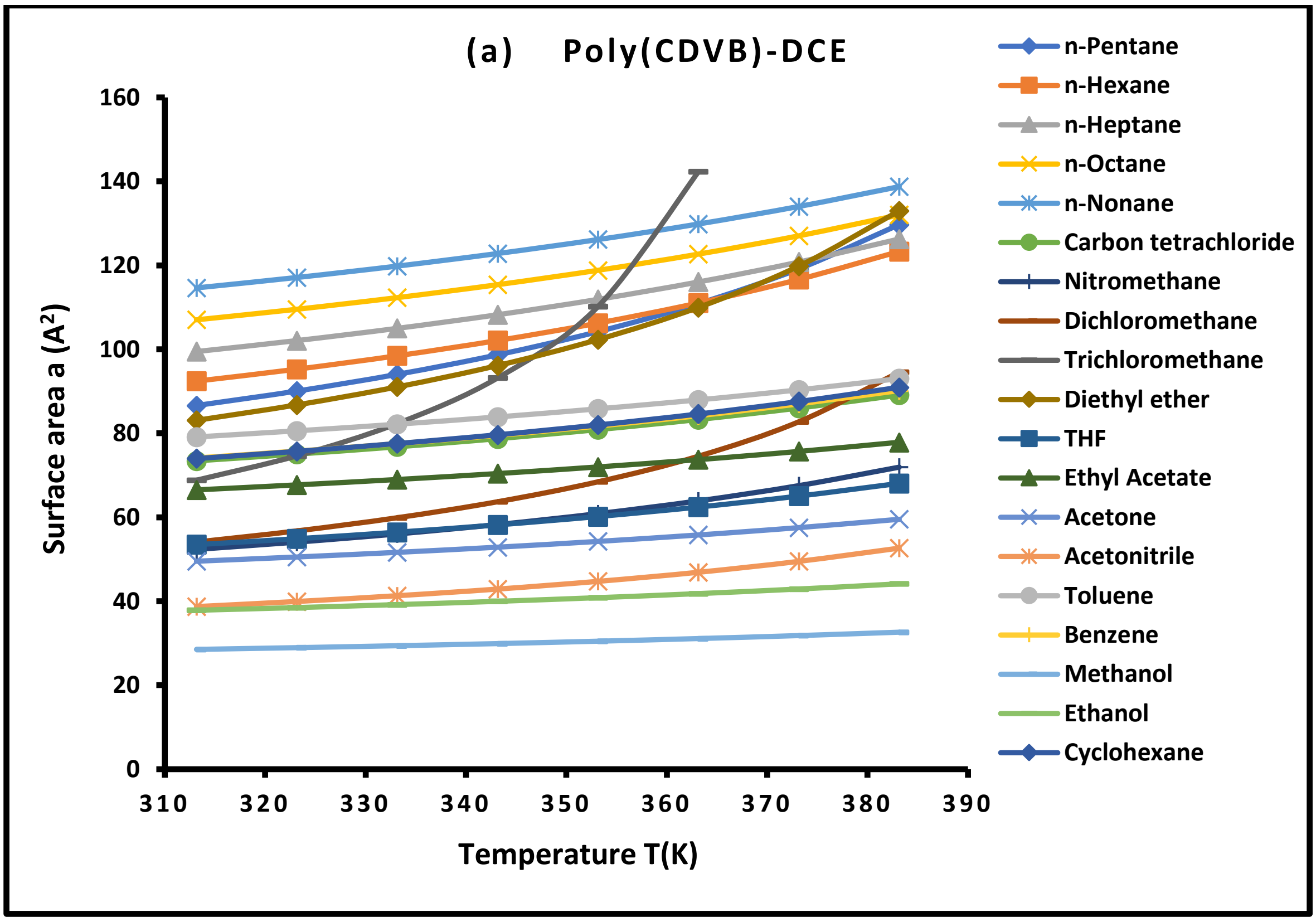

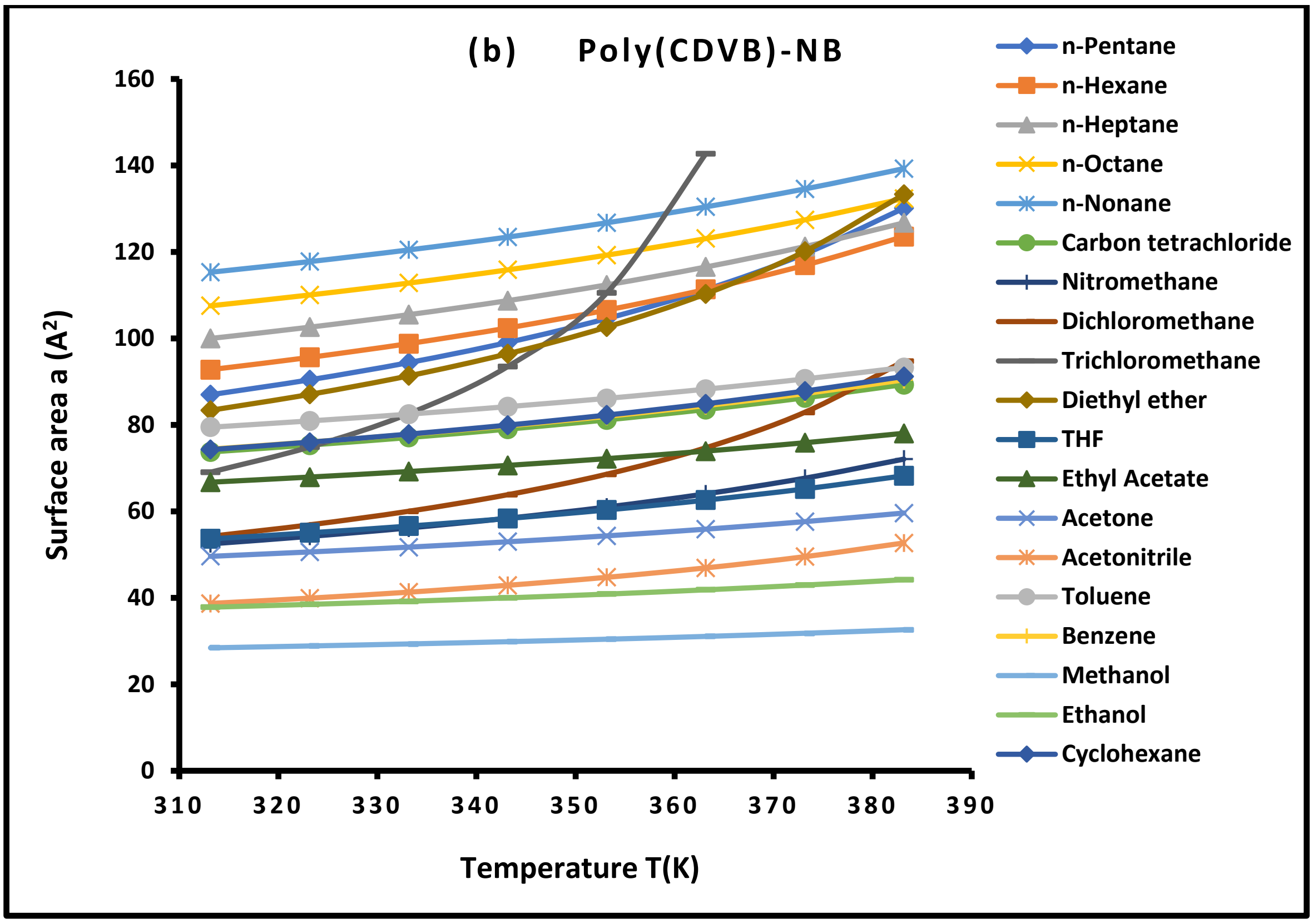
(b) Poly(CDVB)-NB
Surface area a (A^2)
Temperature T(K)
0
20
40
60
80
100
120
140
160
310
320
330
340
350
360
370
380
390
n-Pentane
n-Hexane
n-Heptane
n-Octane
n-Nonane
Carbon tetrachloride
Nitromethane
Dichloromethane
Trichloromethane
Diethyl ether
THF
Ethyl Acetate
Acetone
Acetonitrile
Toluene
Benzene
Methanol
Ethanol
Cyclohexane

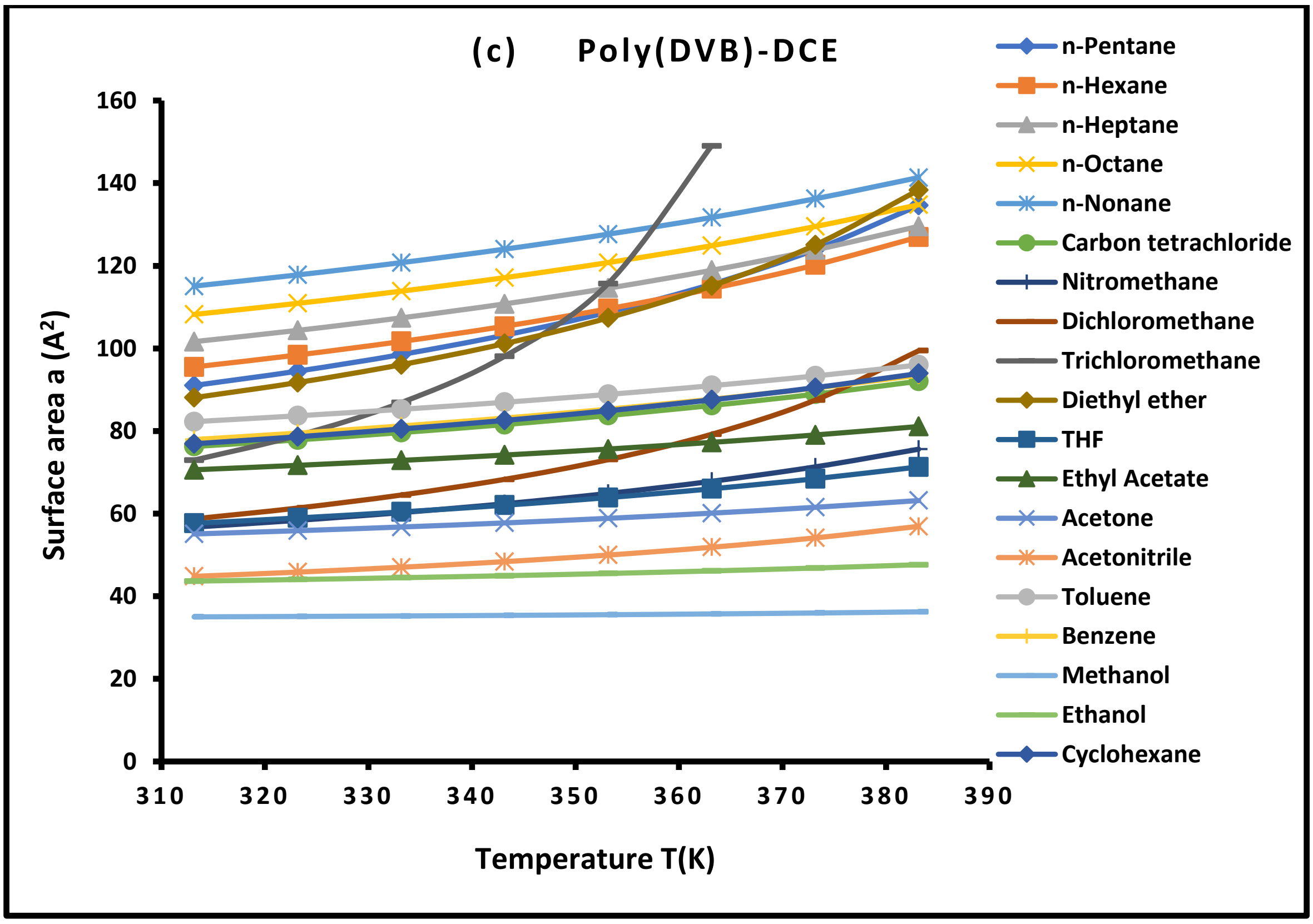
(c) Poly(DVB)-DCE
Surface area a (A^2)
Temperature T(K)
0
20
40
60
80
100
120
140
160
310
320
330
340
350
360
370
380
390
n-Pentane
n-Hexane
n-Heptane
n-Octane
n-Nonane
Carbon tetrachloride
Nitromethane
Dichloromethane
Trichloromethane
Diethyl ether
THF
Ethyl Acetate
Acetone
Acetonitrile
Toluene
Benzene
Methanol
Ethanol
Cyclohexane

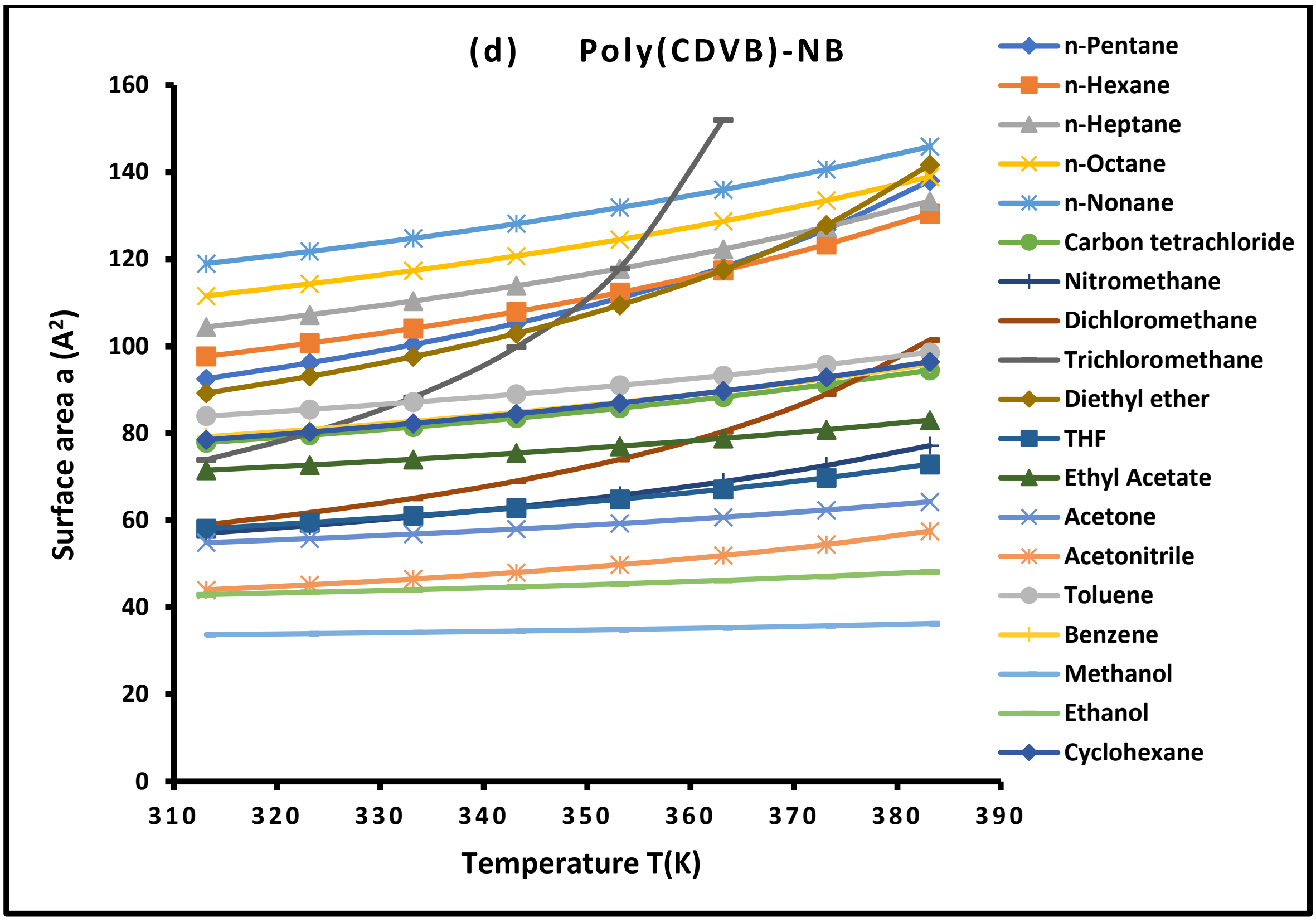

(d) Poly(CDVB)-NB
Surface area a (A²)
Temperature T(K)
0
20
40
60
80
100
120
140
160
310
320
330
340
350
360
370
380
390
n-Pentane
n-Hexane
n-Heptane
n-Octane
n-Nonane
Carbon tetrachloride
Nitromethane
Dichloromethane
Trichloromethane
Diethyl ether
THF
Ethyl Acetate
Acetone
Acetonitrile
Toluene
Benzene
Methanol
Ethanol
Cyclohexane

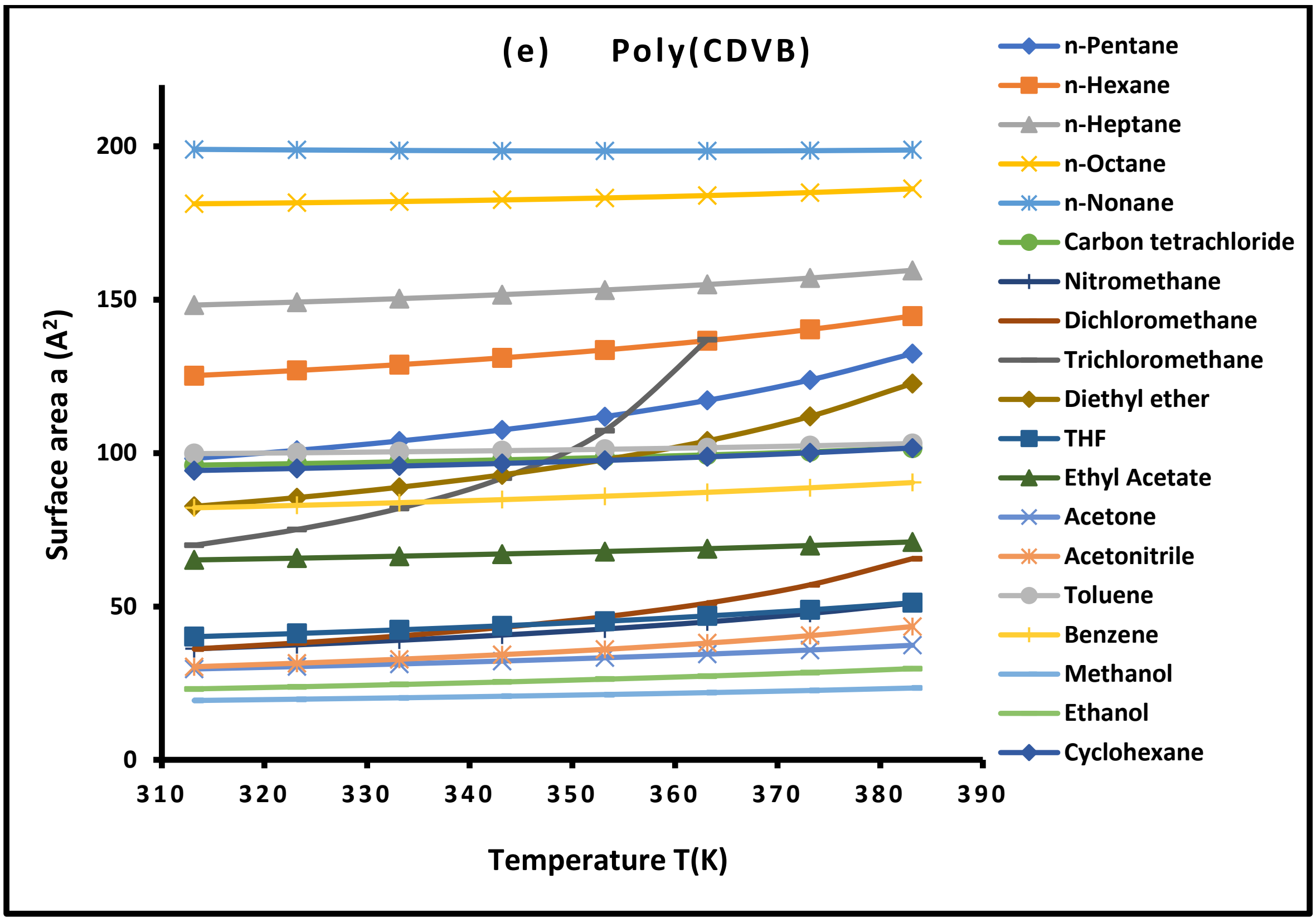

Figure S1. Variations of the surface area (Å²) of solvents adsorbed on the different copolymers as a function of temperature. (a) P1, (b) P2, (c) P3, (d) P4, and (e) P5.

Table S2. Equations $a_{X/S}$ (T), values of $a_{X/S}$ (298.15K), $\lambda_{X/S}$, and linear regression coefficients in linear interpolation.

T(K)	Poly(CDVB)-DCE (P1)			
Solvents	Equations $a_{X/S}$ (T)	$a_{X/S}$ (298.15K)	$\lambda_{X/S}$	R^2
C5	a(T) = 74.232 + 0.599 (T - 298.15)	74.232	0.599	0.9651
C6	a(T) = 83.966 + 0.435 (T - 298.15)	83.966	0.435	0.9799
C7	a(T) = 92.290 + 0.379 (T - 298.15)	92.290	0.379	0.9847
C8	a(T) = 100.443 + 0.353 (T - 298.15)	100.443	0.353	0.9871
C9	a(T) = 108.333 + 0.341 (T - 298.15)	108.333	0.341	0.9884
CCl4	a(T) = 69.336 + 0.222 (T - 298.15)	69.336	0.222	0.9877
Nitromethane	a(T) = 46.906 + 0.275 (T - 298.15)	46.906	0.275	0.9753
DCM	a(T) = 46.778 + 0.403 (T - 298.15)	46.778	0.403	0.9766
TCM	a(T) = 55.515 + 0.808 (T - 298.15)	55.515	0.808	0.9805
Diethyl ether	a(T) = 71.460 + 0.600 (T - 298.15)	71.460	0.600	0.9706
THF	a(T) = 49.566 + 0.2069 (T - 298.15)	49.566	0.206	0.9833
Ethyl Acetate	a(T) = 63567 + 0.1617 (T - 298.15)	63.567	0.161	0.9893

Acetone	a(T) = 46.913 + 0.141 (T - 298.15)	46.913	0.141	0.9871
Acetonitrile	a(T) = 34.834 + 0.195 (T - 298.15)	34.834	0.195	0.9744
Toluene	a(T) = 75.518 + 0.197 (T - 298.15)	75.518	0.197	0.9894
Benzene	a(T) = 69.908 + 0.227 (T - 298.15)	69.908	0.227	0.9873
Methanol	a(T) = 27.466 + 0.058 (T - 298.15)	27.466	0.058	0.988
Ethanol	a(T) = 36.192 + 0.089 (T - 298.15)	36.192	0.089	0.9882
Cyclohexane	a(T) = 69.495 + 0.240 (T - 298.15)	69.495	0.240	0.9868
T(K)	Poly(CDVB)-NB (P2)			
Solvents	Equations $a_{X/S}$ (T)	$a_{X/S}$ (298.15K)	$\lambda_{X/S}$	R^2
C5	a(T) = 0.600 (T - 298.15)	74.550	0.600	0.965
C6	a(T) = 0.433 (T - 298.15)	84.326	0.433	0.9798
C7	a(T) = 0.378 (T - 298.15)	92.838	0.378	0.9847
C8	a(T) = 0.351 (T - 298.15)	100.992	0.351	0.987
C9	a(T) = 0.339 (T - 298.15)	109.032	0.339	0.9884
CCl4	a(T) = 0.221 (T - 298.15)	69.653	0.221	0.9879
Nitromethane	a(T) = 0.275 (T - 298.15)	47.001	0.275	0.9755
DCM	a(T) = 0.404 (T - 298.15)	46.881	0.404	0.9766
TCM	a(T) = 1.015 (T - 298.15)	50.562	1.015	0.9549
Diethyl ether	a(T) = 0.688 (T - 298.15)	68.667	0.688	0.9544
THF	a(T) = 0.206 (T - 298.15)	49.703	0.206	0.9835
Ethyl Acetate	a(T) = 0.160 (T - 298.15)	63.794	0.160	0.9895
Acetone	a(T) = 0.142 (T - 298.15)	46.947	0.142	0.9873
Acetonitrile	a(T) = 0.196 (T - 298.15)	34.805	0.196	0.9746
Toluene	a(T) = 0.196 (T - 298.15)	75.880	0.196	0.9896
Benzene	a(T) = 0.227 (T - 298.15)	70.172	0.227	0.9875
Methanol	a(T) = 0.059 (T - 298.15)	27.326	0.059	0.9883
Ethanol	a(T) = 0.090 (T - 298.15)	36.169	0.090	0.9884
Cyclohexane	a(T) = 0.239 (T - 298.15)	69.807	0.239	0.987
T(K)	Poly(DVB)-DCE (P3)			
Solvents	Equations $a_{X/S}$ (T)	$a_{X/S}$ (298.15K)	$\lambda_{X/S}$	R^2
C5	a(T) = 78.479 + 0.6057 (T - 298.15)	78.479	0.6057	0.9647
C6	a(T) = 86.880 + 0.4426 (T - 298.15)	86.880	0.4426	0.9798
C7	a(T) = 94.155 + 0.3942 (T - 298.15)	94.155	0.3942	0.9848
C8	a(T) = 101.211 + 0.3756 (T - 298.15)	101.211	0.3756	0.9872
C9	a(T) = 108.219 + 0.372 (T - 298.15)	108.219	0.372	0.9886
CCl4	a(T) = 72.114 + 0.2234 (T - 298.15)	72.114	0.2234	0.9876
Nitromethane	a(T) = 51.469 + 0.2642 (T - 298.15)	51.469	0.2642	0.9746
DCM	a(T) = 51.388 + 0.4038 (T - 298.15)	51.388	0.4038	0.9759
TCM	a(T) = 53.854 + 1.0466 (T - 298.15)	53.854	1.0466	0.9544
Diethyl ether	a(T) = 76.354 + 0.6035 (T - 298.15)	76.354	0.6035	0.9702
THF	a(T) = 54.451 + 0.1786 (T - 298.15)	54.451	0.1786	0.988

Ethyl Acetate	a(T) = 68.165 + 0.1398 (T - 298.15)	68.165	0.1398	0.9921
Acetone	a(T) = 53.169 + 0.107 (T - 298.15)	53.169	0.107	0.9901
Acetonitrile	a(T) = 41.949 + 0.1533 (T - 298.15)	41.949	0.1533	0.9811
Toluene	a(T) =79.065 + 0.1835 (T - 298.15)	79.065	0.1835	0.9924
Benzene	a(T) = 74.297 + 0.2066 (T - 298.15)	74.297	0.2066	0.9909
Methanol	a(T) = 34.709 + 0.0154 (T - 298.15)	34.709	0.0154	0.9807
Ethanol	a(T) = 42.596 + 0.0561 (T - 298.15)	42.596	0.0561	0.9853
Cyclohexane	a(T) = 72.393 + 0.2404 (T - 298.15)	72.393	0.2404	0.9867
T(K)	Poly(DVB)-NB (P4)			
Solvents	Equations $a_{X/S}$ (T)	$a_{X/S}$ (298.15K)	$\lambda_{X/S}$	R²
C5	a(T) = 79.451 + 0.632 (T - 298.15)	79.451	0.632	0.965
C6	a(T) = 88.658 + 0.4616 (T - 298.15)	88.658	0.4616	0.9799
C7	a(T) = 96.636 + 0.4089 (T - 298.15)	96.636	0.4089	0.9848
C8	a(T) = 104.330 + 0.3869 (T - 298.15)	104.330	0.3869	0.9872
C9	a(T) = 111.975 + 0.3805 (T - 298.15)	111.975	0.3805	0.9886
CCl4	a(T) = 73.481 + 0.2356 (T - 298.15)	73.481	0.2356	0.9878
Nitromethane	a(T) = 51.396 + 0.2819 (T - 298.15)	51.396	0.2819	0.975
DCM	a(T) = 51.294 + 0.4221 (T - 298.15)	51.294	0.4221	0.9764
TCM	a(T) = 54.278 + 1.0765 (T - 298.15)	54.278	1.0765	0.9547
Diethyl ether	a(T) = 73.763 + 0.7228 (T - 298.15)	73.763	0.7228	0.954
THF	a(T) = 54.062 + 0.2088 (T - 298.15)	54.062	0.2088	0.9831
Ethyl Acetate	a(T) = 68.513 + 0.1631 (T - 298.15)	68.513	0.1631	0.9892
Acetone	a(T) = 52.352 + 0.1329 (T - 298.15)	52.352	0.1329	0.9866
Acetonitrile	a(T) = 40.217 + 0.1891 (T - 298.15)	40.217	0.1891	0.9734
Toluene	a(T) = 80.131 + 0.2076 (T - 298.15)	80.131	0.2076	0.9895
Benzene	a(T) = 74.775 + 0.236 (T - 298.15)	74.775	0.236	0.9873
Methanol	a(T) = 33.025 + 0.0363 (T - 298.15)	33.025	0.0363	0.9846
Ethanol	a(T) = 41.546 + 0.0742 (T - 298.15)	41.546	0.0742	0.9871
Cyclohexane	a(T) = 74.284 + 0.238 (T - 298.15)	74.284	0.238	0.9907
T(K)	Poly(DVB) (P5)			
Solvents	Equations $a_{X/S}$ (T)	$a_{X/S}$ (298.15K)	$\lambda_{X/S}$	R²
C5	a(T) = 90.230 + 0.4188 (T - 298.15)	90.230	0.4188	0.974
C6	a(T) = 120.638 + 0.2478 (T - 298.15)	120.638	0.2478	0.982
C7	a(T) = 145.541 + 0.1453 (T - 298.15)	145.541	0.1453	0.9824
C8	a(T) = 180.079 + 0.0602 (T - 298.15)	180.079	0.0602	0.9677
C9	198.67	198.668	198.67	
CCl4	a(T) = 94.516 + 0.0784 (T - 298.15)	94.516	0.0784	0.979
Nitromethane	a(T) = 32.039 + 0.2088 (T - 298.15)	32.039	0.2088	0.9754
DCM	a(T) = 30.792 + 0.2947 (T - 298.15)	30.792	0.2947	0.9771

TCM	a(T) = 53.197 + 0.9153 (T - 298.15)	53.197	0.9153	0.9528
Diethyl ether	a(T) = 75.041 + 0.4223 (T - 298.15)	75.041	0.4223	0.9804
THF	a(T) = 37.156 + 0.1558 (T - 298.15)	37.156	0.1558	0.9829
Ethyl Acetate	a(T) = 63.588 + 0.0832 (T - 298.15)	63.588	0.0832	0.986
Acetone	a(T) = 27.568 + 0.1092 (T - 298.15)	27.568	0.1092	0.9871
Acetonitrile	a(T) = 26.679 + 0.1834 (T - 298.15)	26.679	0.1834	0.9749
Toluene	a(T) = 98.876 + 0.0464 (T - 298.15)	98.876	0.0464	0.9735
Benzene	a(T) = 79.928 + 0.1161 (T - 298.15)	79.928	0.1161	0.9834
Methanol	a(T) = 18.279 + 0.0574 (T - 298.15)	18.279	0.0574	0.9881
Ethanol	a(T) = 21.398 + 0.0935 (T - 298.15)	21.398	0.0935	0.989
Cyclohexane	a(T) = 92.311 + 0.1035 (T - 298.15)	92.311	0.1035	0.9804

Table S3. Equations $\lambda_{X/S}(\mathcal{S})$, $a_{X/S}(\mathcal{S})$ (298.15K), and linear regression coefficients in linear and quadratic interpolations.

Solvents	Linear interpolation $\lambda_{X/S}(\mathcal{S})$	R²	Quadratic interpolation $\lambda_{X/S}(\mathcal{S})$	R²
C5	λ_(X/S) = 0.0003 S + 0.1678	0.8896	λ_(X/S) = -9E-07 S2 + 0.0022S - 0.7728	0.9772
C6	λ_(X/S) = 0.0003 S - 0.0126	0.9101	λ_(X/S) = -8E-07 S2 + 0.0021S - 0.8815	0.9817
C7	λ_(X/S) = 0.0004 S - 0.1842	0.9243	λ_(X/S) = -9E-07 S2 + 0.0024S - 1.1656	0.9828
C8	λ_(X/S) = 0.0006 S - 0.3556	0.9337	λ_(X/S) = -1E-06 S2 + 0.0029S - 1.4985	0.9842
C9	λ_(X/S) = 0.0006 S - 0.4879	0.9372	λ_(X/S) = -1E-06 S2 + 0.0032S - 1.7634	0.9833
CCl4	λ_(X/S) = 0.0003 S - 0.118	0.9197	λ_(X/S) = -6E-07 S2 + 0.0016S - 0.7682	0.9912
Nitromethane	λ_(X/S) = 0.0001 S + 0.1269	0.8231	λ_(X/S) = -4E-07 S2 + 0.001S - 0.3285	0.9954
DCM	λ_(X/S) = 0.0002 S + 0.1461	0.8811	λ_(X/S) = -6E-07 S2 + 0.0014S - 0.4559	0.9819
TCM	λ_(X/S) = 0.0015 S - 1.0505	0.831	λ_(X/S) = -5E-06 S2 + 0.012S - 6.2358	0.951
Diethyl ether	λ_(X/S) = 0.0004 S + 0.161	0.5976	λ_(X/S) = -2E-06 S2 + 0.0055S - 2.3649	0.9442
THF	λ_(X/S) = 7E-05 S + 0.1079	0.5207	λ_(X/S) = -5E-07 S2 + 0.0012S - 0.4582	0.9581
Ethyl Acetate	λ_(X/S) = 0.0001 S - 0.0067	0.7769	λ_(X/S) = -6E-07 S2 + 0.0013S - 0.5832	0.9899
Acetone	λ_(X/S) = 0.0001 S - 0.027	0.6833	λ_(X/S) = -6E-07 S2 + 0.0014S - 0.6804	0.9402
Acetonitrile	λ_(X/S) = 0.0001 S + 0.0593	0.9718	λ_(X/S) = 1E-07 S2 - 1E-04S + 0.1613	0.983
Toluene	λ_(X/S) = 0.0003 S - 0.1485	0.8842	λ_(X/S) = -8E-07 S2 + 0.0019S - 0.9839	0.9971
Benzene	λ_(X/S) = 0.0002 S - 0.0198	0.8202	λ_(X/S) = -7E-07 S2 + 0.0017S - 0.7842	0.9957

Methanol	λ_(X/S) = 0.0001 S - 0.0626	0.5824	λ_(X/S) = 7E-07 S2 - 0.0014S + 0.6879	0.9573
Ethanol	λ_(X/S) = 0.0002 S - 0.1151	0.962	λ_(X/S) = 2E-07 S2 - 0.0003S + 0.0987	0.9838
Cyclohexane	λ_(X/S) = 0.0002 S - 0.0825	0.9557	λ_(X/S) = -5E-07 S2 + 0.0012S - 0.5529	0.9999

Solvents	Linear interpolation $a_{X/S}(\mathcal{S})$ (298.15K)	R^2	Quadratic interpolation $a_{X/S}(\mathcal{S})$ (298.15K)	R^2
C5	a_(X/S) = 0.0003 S + 0.1678	0.8896	a_(X/S) = -9E-07 S2 + 0.0022 S - 0.7728	0.9772
C6	a_(X/S) = 0.0003 S - 0.0126	0.9101	a_(X/S) = -8E-07 S2 + 0.0021 S - 0.8815	0.9817
C7	a_(X/S) = 0.0004 S - 0.1842	0.9243	a_(X/S) = -9E-07 S2 + 0.0024 S - 1.1656	0.9828
C8	a_(X/S) = 0.0006 S - 0.3556	0.9337	a_(X/S) = -1E-06 S2 + 0.0029 S - 1.4985	0.9842
C9	a_(X/S) = 0.0006 S - 0.4879	0.9372	a_(X/S) = -1E-06 S2 + 0.0032 S - 1.7634	0.9833
CCl4	a_(X/S) = 0.0003 S - 0.118	0.9197	a_(X/S) = -6E-07 S2 + 0.0016 S - 0.7682	0.9912
Nitromethane	a_(X/S) = 0.0001 S + 0.1269	0.8231	a_(X/S) = -4E-07 S2 + 0.001 S - 0.3285	0.9954
DCM	a_(X/S) = 0.0002 S + 0.1461	0.8811	a_(X/S) = -6E-07 S2 + 0.0014 S - 0.4559	0.9819
TCM	a_(X/S) = 0.0015 S - 1.0505	0.831	a_(X/S) = -5E-06 S2 + 0.012 S - 6.2358	0.951
Diethyl ether	a_(X/S) = 0.0004 S + 0.161	0.5976	a_(X/S) = -2E-06 S2 + 0.0055 S - 2.3649	0.9442
THF	a_(X/S) = 7E-05 S + 0.1079	0.5207	a_(X/S) = -5E-07 S2 + 0.0012 S - 0.4582	0.9581
Ethyl Acetate	a_(X/S) = 0.0001 S - 0.0067	0.7769	a_(X/S) = -6E-07 S2 + 0.0013 S - 0.5832	0.9899
Acetone	a_(X/S) = 0.0001 S - 0.027	0.6833	a_(X/S) = -6E-07 S2 + 0.0014 S - 0.6804	0.9402
Acetonitrile	a_(X/S) = 0.0001 S + 0.0593	0.9718	a_(X/S) = 1E-07 S2 - 1E-04 S + 0.1613	0.983
Toluene	a_(X/S) = 0.0003 S - 0.1485	0.8842	a_(X/S) = -8E-07 S2 + 0.0019 S - 0.9839	0.9971
Benzene	a_(X/S) = 0.0002 S - 0.0198	0.8202	a_(X/S) = -7E-07 S2 + 0.0017 S - 0.7842	0.9957
Methanol	a_(X/S) = 0.0001 S - 0.0626	0.5824	a_(X/S) = 7E-07 S2 - 0.0014 S + 0.6879	0.9573
Ethanol	a_(X/S) = 0.0002 S - 0.1151	0.962	a_(X/S) = 2E-07 S2 - 0.0003 S + 0.0987	0.9838
Cyclohexane	a_(X/S) = 0.0002 S - 0.0825	0.9557	a_(X/S) = -5E-07 S2 + 0.0012 S - 0.5529	0.9999

Table S4. Equations of $a_2(\mathcal{S})$, $a_1(\mathcal{S})$, $a_0(\mathcal{S})$, $a_{X/S}(\mathcal{S}, 298.15K)$, and linear regression coefficients in linear interpolation.

Linear interpolation				
Solvents	a_2 ($\mathcal{S}$)	R^2	a_1 ($\mathcal{S}$)	R^2
n-Pentane	a2(S) = 4E-06S + 0.0011	0.9000	a1(S) = 0.001S + 2.0765	0.8153
n-Hexane	a2(S) = 1.680E-06S + 9.472E-04	0.8881	a1(S) = 0.0009S + 0.6122	0.8801
n-Heptane	a2(S) = 2.056E-06S - 2.800E-04	0.9129	a1(S) = 0.001S - 0.0475	0.9105
n-Octane	a2(S) = 2.467E-06S - 1.143E-03	0.9259	a1(S) = 0.0012S - 0.4552	0.9249
n-Nonane	a2(S) = 2.862E-06S - 1.815E-03	0.9349	a1(S) = 0.0013S - 0.765	0.9315
Carbon tetrachloride	a2(S) = 1E-06S - 0.0003	0.9176	a1(S) = 0.0006S - 0.1173	0.9173
Nitromethane	a2(S) = 9E-07S + 0.001	0.8516	a1(S) = 0.0005S + 0.5461	0.8556
Dichloromethane	a2(S) = 4E-06S + 0.0023	0.8914	a1(S) = 0.0023S + 1.392	0.8927
Trichloromethane	a2(S) = 2E-05S + 0.0005	0.9199	a1(S) = 0.0117S + 3.9914	0.8724
Diethyl ether	a2(S) = 2E-06S + 0.0043	0.8402	a1(S) = 0.0014S + 2.6549	0.8338
THF	a2(S) = 5E-07S + 0.0006	0.8057	a1(S) = 0.0003S + 0.3515	0.8148
Ethyl Acetate	a2(S) = 6E-07S + 7E-05	0.8580	a1(S) = 0.0003S + 0.0602	0.8718
Acetone	a2(S) = 3E-07S + 0.0004	0.9910	a1(S) = 0.0003S + 0.09	0.9925
Acetonitrile	a2(S) = 2E-07S + 0.0013	0.9352	a1(S) = 0.0001S + 0.7178	0.9273
Toluene	a2(S) = 1.147E-06S - 4.769E-04	0.9165	a1(S) = 0.0006S - 0.226	0.956
Benzene	a2(S) = 9E-07S + 4E-05	0.9009	a1(S) = 0.0005S + 0.0101	0.9512
Methanol	a2(S) = 4E-07S - 0.0002	0.9077	a1(S) = 0.0002S - 0.0688	0.8855
Ethanol	a2(S) = 5E-07S - 0.0002	0.9003	a1(S) = 0.0002S + 0.0079	0.9639
CycloHexane	a2(S) = 1.194E-06S - 1.618E-04	0.9100	a1(S) = 0.0006S - 0.0356	0.9112
Linear interpolation				
Solvents	a_0 (S)	R^2	$a_{X/S}$ (298.15K) (S)	R^2
n-Pentane	a0(S) = 0.2202S + 292.68	0.8311	a(T0) = -0.0566S + 161.08	0.952
n-Hexane	a0(S) = 0.139S + 150.79	0.8579	a(T0) = -0.0583S + 168.34	0.9388
n-Heptane	a0(S) = 0.1352S + 89.522	0.8562	a(T0) = -0.0893S + 215.3	0.9604
n-Octane	a0(S) = 0.1164S + 89.447	0.8541	a(T0) = -0.1368S + 284.45	0.9627
n-Nonane	a0(S) = 0.1243S + 72.031	0.8918	a(T0) = -0.1571S + 318.27	0.9599
Carbon tetrachloride	a0(S) = 0.0798S + 50.195	0.8941	a(T0) = -0.0403S + 126.21	0.9458
Nitromethane	a0(S) = 0.1081S + 87.114	0.8838	a(T0) = 0.0329S + 10.53	0.8949

Dichloromethane	a0(S) = 0.3863S + 220.88	0.8956	a(T0) = 0.0385S + 7.6139	0.8999
Trichloromethane	a0(S) = 1.5901S + 1068.6	0.8934	0.89615	
Diethyl ether	a0(S) = 0.2869S + 390.67	0.8603	a(T0) = 0.0253S + 51.816	0.9589
THF	a0(S) = 0.0649S + 65.656	0.8754	a(T0) = 0.0425S - 0.9335	0.9158
Ethyl Acetate	a0(S) = 0.0528S + 48.766	0.8842	a(T0) = 0.0417S + 13.037	0.898
Acetone	a0(S) = 0.0576S + 27.058	0.894	a(T0) = 0.0409S - 2.2207	0.8976
Acetonitrile	a0(S) = 0.0299S + 125.99	0.6977	a(T0) = 0.0324S - 2.8594	0.8566
Toluene	a0(S) = 0.055S + 69.735	0.8675	a(T0) = -0.0368S + 127.56	0.918
Benzene	a0(S) = 0.0596S + 81.458	0.8616	a(T0) = -0.0225S + 102.97	0.9315
Methanol	a0(S) = 0.0159S + 27.34	0.7926	a(T0) = 0.0374S - 18.427	0.8735
Ethanol	a0(S) = 0.0275S + 32.296	0.8592	a(T0) = 0.0453S - 19.033	0.9266
Cyclohexane	a0(S) = 0.0684S + 78.007	0.8704	a(T0) = -0.0361S + 121.48	0.9292

S1. Temperature and surface morphology jointly determine the adsorbed molecular footprint

After extensive and rigorous thermodynamic processing of the complete dataset, it was demonstrated that the adsorption of all studied solvents on DVB-copolymer surfaces—across the full range of temperatures and specific surface area $\mathcal{S}$ —obeys a universal surface-area equation. For any solvent X adsorbed on a surface, the molecular interfacial area $a_{X/S}$ satisfies the general linear-in-temperature form ($R^2 > 0.95$):

$$a_{X/S}(T, \mathcal{S}) = a_{X/S}(T_0, \mathcal{S}) + \lambda_{X/S}(\mathcal{S})\,(T - T_0) \quad \text{(S1)}$$

$$\lambda_{X/S} = \left(\frac{\partial a_{X/S}}{\partial T}\right)_{\mathcal{S}} \quad \text{(S2)}$$

where T_0 is a reference temperature (chosen here equal to 298.15K), $a_{X/S}(T_0, \mathcal{S})$ is the baseline molecular footprint, and $\lambda_{X/S}(\mathcal{S})$ represents the thermal expansion coefficient of the 2D adsorbed state at a given polymer recovery fraction.

Equations $a_{X/S}$ (T), values of $a_{X/S}$ (298.15K), $\lambda_{X/S}$, and linear regression coefficients in linear interpolation are given in Table S2**.**

The observed variations of $a_{X/S}(T, \mathcal{S})$ reveal fundamental changes in the configurational freedom, adsorption geometry, lateral packing, and energetic fields imposed by the copolymer-solvent interface. These results provide direct insight into the two-dimensional thermodynamics and molecular mobility of adsorbed species on copolymer surfaces. Consequently equation 15 can be considered as a universal law of molecular surface area on copolymer-solvent interfaces. The parameters $a_{X/S}(T_0, \mathcal{S})$ and $T_{X/S}(\mathcal{S})$ given in Table S2 showed important variations as a function of the specific surface area. Unlike existing surface energy models, this relation 1 governs the two-dimensional adsorption footprint and its coupled dependence on temperature and surface composition.

The linear variations of $a_{X/S}(T_0, \mathcal{S})$ and $\lambda_{S/X}(\mathcal{S})$ as a function of SSA are presented in Table S3 with good linear regression coefficients (in general 0.9544 < R^2 < 0.9900). Whereas the improved quadratic equations were given in table S3. Therefore, a key result is that both $a_{X/S}(T_0, \mathcal{S})$ and $\lambda_{X/S}(\mathcal{S})$ follow simple linear analytical forms as functions of $\mathcal{S}$:

$$a_{X/S}(T_0, \mathcal{S}) = \mu_0 + \mu_1 \mathcal{S} \quad S(3)$$

$$\lambda_{X/S}(\mathcal{S}) = \lambda_0 + \lambda_1 \mathcal{S} \quad (S4)$$

$$a_{X/S}(T, \mathcal{S}) = (\mu_0 + \mu_1 \mathcal{S}) + (\lambda_0 + \lambda_1 \mathcal{S})(T - T_0) \quad (S5)$$

$$(\frac{\partial a_{X/S}}{\partial T})_{\mathcal{S}} = \lambda_0 + \lambda_1 \mathcal{S} \quad (S6)$$

$$(\frac{\partial a_{X/S}}{\partial \mathcal{S}})_T = \mu_1 + \lambda_1 (T - T_0) \quad (S7)$$

$$(\frac{\partial^2 a_{X/S}}{\partial \mathcal{S}\, \partial T})_{\mathcal{S},T} = \lambda_1 \quad (S8)$$

In quadratic interpolation, one obtained the following equations:

$$a_{X/S}(T_0, \mathcal{S}) = \mu_0 + \mu_1 \mathcal{S} + \mu_2 \mathcal{S}^2 \quad (S9)$$

$$\lambda_{X/S}(\mathcal{S}) = \lambda_0 + \lambda_1 \mathcal{S} + \lambda_2 \mathcal{S}^2 \quad (S10)$$

And

$$a_{X/S}(T, \mathcal{S}) = (\mu_0 + \mu_1 \mathcal{S} + \mu_2 \mathcal{S}^2) + (\lambda_0 + \lambda_1 \mathcal{S} + \lambda_2 \mathcal{S}^2)(T - T_0) \quad (S11)$$

$$(\frac{\partial a_{X/S}}{\partial T})_{\mathcal{S}} = \lambda_0 + \lambda_1 \mathcal{S} + \lambda_2 \mathcal{S}^2 \quad (S12)$$

$$(\frac{\partial a_{X/S}}{\partial \mathcal{S}})_T = \mu_1 + 2\mu_2 \mathcal{S} + (\lambda_1 + 2\lambda_2)\mathcal{S}(T - T_0) \quad (S13)$$

$$(\frac{\partial^2 a_{X/S}}{\partial \mathcal{S}\, \partial T})_{\mathcal{S},T} = \lambda_1 + 2\lambda_2 \mathcal{S} \quad (S14)$$

$$(\frac{\partial^3 a_{X/S}}{\partial^2 \mathcal{S}\, \partial T})_{\mathcal{S},T} = 2\lambda_2 \quad (S15)$$

S2. The adsorbed molecular footprint follows a common quadratic thermo-geometric relationship

However, the quadratic interpolation exhibits a highest regression with $R^2 \geq 0.99$ in all cases (Table S4). In such case:

$$a_{X/S}(T, \mathcal{S}) = a_2(\mathcal{S})\, T^2 + a_1(\mathcal{S})\, T + a_0(\mathcal{S}) \quad (S16)$$

$$\left(\frac{\partial a_{X/S}}{\partial T}\right)_{\mathcal{S}} = 2a_2(\mathcal{S})T + a_1(\mathcal{S}) \quad (S17)$$

This implies the thermal expansion relation of $\lambda_{X/S}(\mathcal{S}, T)$ function in $(\mathcal{S}, T)$:

$$\lambda_{X/S}(\mathcal{S}, T) = 2a_2(\mathcal{S})T + a_1(\mathcal{S}) \quad (S18)$$

$$\left(\frac{\partial^2 a_{X/S}}{\partial T^2}\right)_{\mathcal{S}} = 2a_2(\mathcal{S}) = (\frac{\partial \lambda_{X/S}}{\partial T})_{\mathcal{S}} \quad (S19)$$

With linear variations of the different coefficients in $\mathcal{S}$:

$$a_2(\mathcal{S}) = p_1\, \mathcal{S} + p_0 \quad (S20)$$

$$a_1(\mathcal{S}) = q_1\, \mathcal{S} + q_0 \quad (S21)$$

$$a_0(\mathcal{S}) = r_1\, \mathcal{S} + r_0 \quad (S22)$$

With quadratic variations of the different coefficients in $\mathcal{S}$

$$a_2(\mathcal{S}) = p_2\, \mathcal{S}^2 + p_1\, \mathcal{S} + p_0 \quad (S23)$$

$$a_1(\mathcal{S}) = q_2\, \mathcal{S}^2 + q_1\, \mathcal{S} + q_0 \quad (S24)$$

$$a_0(\mathcal{S}) = r_2\, \mathcal{S}^2 + r_1\, \mathcal{S} + r_0 \quad (S25)$$

Where the parameters p_i, q_i, and r_i are constant for $i \in \{1, 2, 3\}$, a_0, a_1, and a_2 represent the surface coefficients of $a_{X/S}(T, \mathcal{S})$ while $a_1(\mathcal{S})$ is the thermal expansion with q_0, q_1, and q_2 the thermal expansion terms of $a_1(\mathcal{S})$.

Thus, the full surface-area function may be expressed as a polynomial in $\mathcal{S}$ and in T:

$$a_{X/S}(T, \mathcal{S}) = (p_2\, \mathcal{S}^2 + p_1\, \mathcal{S} + p_0)\, T^2 + (q_2\, \mathcal{S}^2 + q_1\, \mathcal{S} + q_0)\, T + (r_2\, \mathcal{S}^2 + r_1\, \mathcal{S} + r_0) \quad \text{(S26)}$$

Or

$$a_{X/S}(T, \mathcal{S}) = p_2\, \mathcal{S}^2 T^2 + p_1\, \mathcal{S} T^2 + p_0 T^2 + q_2\, \mathcal{S}^2 T + q_1\, \mathcal{S} T + q_0\, T + r_2\, \mathcal{S}^2 + r_1\, \mathcal{S} + r_0 \quad \text{(S27)}$$

$$\left(\frac{\partial a_{X/S}}{\partial T}\right)_{\mathcal{S}} = 2(p_2\, \mathcal{S}^2 + p_1\, \mathcal{S} + p_0) T + (q_2\, \mathcal{S}^2 + q_1\, \mathcal{S} + q_0) \quad \text{(S28)}$$

$$\left(\frac{\partial a_{X/S}}{\partial \mathcal{S}}\right)_{T} = (2p_2\, \mathcal{S} + p_1)\, T^2 + (2q_2\, \mathcal{S} + q_1)\, T + (2r_2\, \mathcal{S} + r_1) \quad \text{(S29)}$$

$$\left(\frac{\partial^2 a_{X/S}}{\partial T^2}\right)_{\mathcal{S}} = 2(p_2\, \mathcal{S}^2 + p_1\, \mathcal{S} + p_0) \quad \text{(S30)}$$

$$\left(\frac{\partial^2 a_{X/S}}{\partial \mathcal{S}^2}\right)_{T} = 2p_2 T^2 + 2q_2 T + 2r_2 \quad \text{(S31)}$$

$$\left(\frac{\partial^3 a_{X/S}}{\partial T\, \partial \mathcal{S}^2}\right)_{T,\mathcal{S}} = 4p_2 T + 2q_2 \quad \text{(S32)}$$

$$\left(\frac{\partial^3 a_{X/S}}{\partial T^3}\right)_{\mathcal{S}} = 0; \left(\frac{\partial^3 a_{X/S}}{\partial \mathcal{S}^3}\right)_{T} = 0 \quad \text{(S33)}$$

$$\left(\frac{\partial^3 a_{X/S}}{\partial \mathcal{S}\, \partial T^2}\right)_{\mathcal{S},T} = 2(2p_2\, \mathcal{S} + p_1) \quad \text{(S34)}$$

$$\left(\frac{\partial^4 a_{X/S}}{\partial \mathcal{S}^2\, \partial T^2}\right)_{\mathcal{S},T} = 4p_2 \quad \text{(S35)}$$

Table S5. Quantitative correlations between thermo-geometric descriptors of the adsorbed molecular footprint and the Lewis acid–base parameters obtained from the five-parameter model for diethyl ether (Lewis basic probe) and dichloromethane (Lewis acidic probe). The parameters a_0, a_1, and $a_2$correspond to the coefficients of the universal quadratic relation $a_{X/S}(T, \mathcal{S})$, while λdenotes the effective thermal expansion coefficient of the adsorbed phase. The high coefficients of determination demonstrate a direct coupling between adsorption geometry and donor–acceptor energetics.

**Solvent**	**Diethyl ether**		**Dichloromethane**	
**Parameters y**	**Equations y =f(KA)**	**R²**	**Equations y =f(KA)**	**R²**
a2	a2 = -0.016KA² + 0.006KA + 0.0076	0.9658	a2 = -0.0186KA² + 0.0055KA + 0.0068	0.9851
a1	a1 = -9.714KA² + 3.456KA + 4.394	0.9637	a1 = -11.812KA² + 3.5827KA + 4.2159	0.9857
a0	a0 = -1690.7KA² + 642.1KA + 743.6	0.9575	a0 = -2128.8KA² + 670.83KA + 703.33	0.9866
a(298.15K)	y = -198.88KA² + 99.49KA + 82.29	0.7829	y = -259.73KA² + 93.105KA + 54.723	0.9803
a(323.15K)	y = -201.42KA² + 98.31KA + 85.78	0.816	y = -253.22KA² + 89.237KA + 55.625	0.9803
λ (298.15)	y = -19.134KA² + 6.727KA + 8.744	0.9648	y = -22.899KA² + 6.8728KA + 8.2969	0.9854

λ (323.15)	y = -19.924KA² + 7.002KA + 9.109	0.9648	y = -23.829KA² + 7.1487KA + 8.6391	0.9854
**Solvent**	**Diethyl ether**		**Dichloromethane**	
**Parameters y**	**Equations y =f(KD)**	**R^2**	**Equations y =f(KD)**	**R^2**
a2	a2 = -0.0019KD + 0.0079	0.9582	a2 = -0.0028KD + 0.0076	0.9791
a1	a1 = -1.1284KD + 4.7457	0.9551	a1 = -1.7521KD + 4.6949	0.9802
a0	a0 = -171.59KD + 801.65	0.9431	a0 = -300.45KD + 787.63	0.9819
a(298.15K)	y = 53.887KD² - 65.392KD + 96.966	0.7342	y = -29.805KD + 64.104	0.9752
a(323.15K)	y = 50.326KD² - 63.005KD + 100.07	0.7737	y = -29.989KD + 64.895	0.9757
λ (298.15)	y = -2.2714KD + 9.4444	0.9567	y = -3.4407KD + 9.2312	0.9797
λ (323.15)	y = -2.3673KD + 9.8384	0.9567	y = -3.5823KD + 9.6116	0.9796
**Solvent**	**Diethyl ether**		**Dichloromethane**	
**Parameters y**	**Equations y =f(K)**	**R^2**	**Equations y =f(K)**	**R^2**
a2	a2 = -0.1737K + 0.0076	0.9372	a2 = -0.2586K + 0.0072	0.9723
a1	a1 = -102.09K + 4.5676	0.9314	a1 = -159.85K + 4.4212	0.9721
a0	a0 = -15455K + 774.42	0.9115	a0 = -27385K + 740.64	0.9718
a(298.15K)	y = 892723K² - 8440.9K + 90.15	0.5604	y = -2708.6K + 59.425	0.9594
a(323.15K)	y = 847241K² - 8179.9K + 93.46	0.6308	y = -2728.2K + 60.193	0.962
λ (298.15)	y = -205.66K + 9.0862	0.9344	y = -314.03K + 8.694	0.9722
λ (323.15)	y = -214.35K + 9.4651	0.9345	y = -326.96K + 9.0522	0.9722
**Solvent**	**Diethyl ether**		**Dichloromethane**	
**Parameters y**	**Equations y =f(K2A)**	**R^2**	**Equations y =f(K2A)**	**R^2**
a2	a2 = -246.08K2A² + 0.6816K2A + 0.0071	0.9409	a2 = -430.35K2A² + 1.3483K2A + 0.0061	0.9631
a1	a1 = -138885K2A² + 370.79K2A + 4.315	0.9383	a1 = -261829K2A² + 811.56K2A + 3.7854	0.9638
a0	a0 = -2E+07K2A² + 40255K2A + 752.57	0.9294	a0 = -4E+07K2A² + 131097K2A + 639.86	0.9636
a(298.15K)	y = 2E+06K2A² - 9702.8K2A + 96.777	0.7846	y = -4E+06K2A² + 8989.6K2A + 53.5	0.9485
a(323.15K)	y = 1E+06K2A² - 8385K2A + 99.109	0.802	y = -4E+06K2A² + 9643.7K2A + 53.613	0.9482
λ (298.15)	y = -285621K2A² + 777.25K2A + 8.546	0.9396	y = -518449K2A² + 1615.6K2A + 7.4228	0.9635
λ (323.15)	y = -297925K2A² + 811.33K2A + 8.9007	0.9396	y = -539966K2A² + 1683K2A + 7.7278	0.9635
**Solvent**	**Diethyl ether**		**Dichloromethane**	
**Parameters y**	**Equations y =f(K2D)**	**R^2**	**Equations y =f(K2D)**	**R^2**
a2	a2 = 0.1131K2D + 0.0077	0.9546	a2 = 0.1676K2D + 0.0073	0.9813
a1	a1 = 66.543K2D + 4.6333	0.9503	a1 = 103.67K2D + 4.5219	0.9819
a0	a0 = 10102K2D + 784.49	0.9352	a0 = 17771K2D + 757.93	0.9827
a(298.15K)	y = 296796K2D² + 4959.8K2D + 93.769	0.805	y = 1761.2K2D + 61.151	0.9742

a(323.15K)	y = 282353K2D^2 + 4827.2K2D + 97.017	0.8302	y = 1772.9K2D + 61.926	0.9756
λ (298.15)	y = 133.99K2D + 9.2184	0.9525	y = 203.61K2D + 8.8915	0.9816
λ (323.15)	y = 139.65K2D + 9.6028	0.9526	y = 211.99K2D + 9.2579	0.9816
